# Molecular Evolution of Human Adenovirus (HAdV) Species C

**DOI:** 10.1038/s41598-018-37249-4

**Published:** 2019-01-31

**Authors:** Akshay Dhingra, Elias Hage, Tina Ganzenmueller, Sindy Böttcher, Jörg Hofmann, Klaus Hamprecht, Patrick Obermeier, Barbara Rath, Fabian Hausmann, Thomas Dobner, Albert Heim

**Affiliations:** 1 Hannover Medical School, Institute of Virology, Hannover, Germany; 2 Robert Koch Institut, FG 15, Nationales Referenzzentrum für Poliomyelitis und Enteroviren, Berlin, Germany; 3 Institute of Medical Virology, Helmut-Ruska-Haus, Charité Medical School, Berlin, Germany; 4 Institut für Medizinische Virologie und Epidemiologie der Viruserkrankungen, Universitätsklinikum Tübingen, Tübingen, Germany; 5 Heinrich Pette Institute, Leibniz Institute for Experimental Virology, Hamburg, Germany; 6 German Centre for Infection Research (DZIF), partner site Hannover-, Braunschweig, Germany; 7 Vienna Vaccine Safety Initiative, Berlin, Germany; 8 Laboratoire Chrono-environnement, Université Bourgogne Franche-Comté, Besançon, France

## Abstract

Currently, 88 different Human Adenovirus (HAdV) types are grouped into seven HAdV species A to G. Most types (57) belong to species HAdV-D. Recombination between capsid genes (hexon, penton and fiber) is the main factor contributing to the diversity in species HAdV-D. Noteworthy, species HAdV-C contains so far only five types, although species HAdV-C is highly prevalent and clinically significant in immunosuppressed patients. Therefore, the evolution of species HAdV-C was studied by generating 51 complete genome sequences from circulating strains. Clustering of the whole genome HAdV-C sequences confirmed classical typing results (fifteen HAdV-C1, thirty HAdV-C2, four HAdV-C5, two HAdV-C6). However, two HAdV-C2 strains had a novel penton base sequence and thus were re-labeled as the novel type HAdV-C89. Fiber and early gene region 3 (E3) sequences clustered always with the corresponding prototype sequence but clustering of the E4 region indicated recombination events in 26 out of the 51 sequenced specimens. Recombination of the E1 gene region was detected in 16 circulating strains. As early gene region sequences are not considered in the type definition of HAdVs, evolution of HAdV-C remains on the subtype level. Nonetheless, recombination of the E1 and E4 gene regions may influence the virulence of HAdV-C strains.

## Introduction

Human adenoviruses (HAdVs) are double-stranded, non-enveloped, linear DNA viruses of 34–36 kbp length^1,2^. Currently, there are 88 different HAdV types known, which have been classified into seven species A to G and new adenovirus types continue to emerge^1,3^. Types were exclusively defined as serotypes (in cross-neutralization) up to type 51, for newer types a genotype definition was mostly used which requires either novel sequences or recombinant phylogeny in genes coding for major capsid proteins^4^.

The majority of HAdV types belong to species HAdV-D (57 types) followed by species HAdV-B (16 types) (http://hadvwg.gmu.edu/). Homologous recombination among capsid genes (hexon, penton and fibre) is imperative in contributing to the high diversity of species HAdV-D types. In contrast, rapid selection of novel capsid gene sequences is the major factor for diversity in species HAdV-B^1,3,5,6^, although a few recombinant HAdV types of species B have also been described^7^. Recombination of capsid protein genes may diversify the tissue tropism of novel HAdV types and thus enhance the pathogenicity and virulence of the new viruses^3,8,9^.

Although infections with species HAdV-C types are highly prevalent, evolution of species HAdV-C did not results in multiple novel types, neither by recombination between the main capsid genes nor by rapid selection of e.g. immune escape mutations in the highly variable immunogenic loops of hexon. Despite species HAdV-C comprises only 5 types so far (1, 2, 5, 6 and 57), these are clinically more significant than species HAdV-B and -D in causing severe manifestations in immunocompromised patients, in particular in allogeneic hematopoietic stem cell transplant (HSCT) recipients^10–12^. More than half of adenovirus infections in immunocompromised hosts are associated with species C type 1 and 2^11–14^. HAdV also accounts for 15% of upper respiratory tract infections and 5% of lower respiratory tract infections in adult and pediatric immunocompetent patients^2,15^. Species HAdV-C has been detected in the majority of respiratory tract infections followed by species B^16,17^. After primary infection, HAdV-C DNA can persist in a latent state in lymphoid cells and asymptomatic, intermittent shedding of infectious virus in feces can be observed for many years^10,18,19^. Immunosuppression frequently leads to HAdV reactivation and severe clinical manifestations, such as disseminated adenoviral disease after HSCT^2,8,19–21^.

The high prevalence of infections with HAdV-C types in combination with the long-term latency of HAdV-C DNA should increase the probability of superinfection with another HADV-C type and thus promote the evolution of novel HAdV-C types by intertypic recombination. Still, only 5 types of HAdV-C have been described so far and little is known about the factors affecting pathogenicity and evolution of HAdV-C types and circulating HAdV-C strains. A recent study applying whole genomic sequencing to adenovirus specimens from immunocompromised pediatric patients confirmed the predominance of species HAdV-C strains in this population and detected an infection chain (transmission of HAdV-A31 between the included patients)^22^.

In the study presented here, 51 whole genome HAdV-C sequences were generated by next-generation sequencing of clinical isolates and diagnostic specimens and analyzed for recombinant phylogeny of genomes and diversity of immunogenic capsid proteins. Surprisingly, multiple recombination of early gene regions was found to be predominant in the evolution of these clinically relevant HAdV-C strains, whereas recombination of genes for major capsid proteins was almost absent and therefore only one novel type of species HAdV-C was found.

## Results

### Molecular typing by imputed serology

All specimens were initially typed by sequencing of loops 1 and 2 of the main neutralization determinant ε. This molecular typing procedure gives the same results as the classical serological typing technique, neutralization testing, and thus is called “imputed serology”. 15 (out of the 51) specimens identified as type 1, 30 specimens as type 2, 4 specimens as type 5, 2 specimens as type 6 but none as type 57 (Table 1).

**Table 1:** Origin and characteristics of species HAdV-C strains analyzed in this study (URT: upper respiratory tract, LRT: lower respiratory tract).

Specimen (strain #/species/type)	Year	Imputed serology (partial hexon sequencing)	Source	Derived from immunosuppressed patient (+/−)	Clustering of complete genomic sequence with prototype	Relabelled as novel type	Accession Number
1C2	2000	2	Feces	+	2	−	MH121072
2C2	2000	2	Blood	+	2	−	MH121070
3C2	2002	2	Blood	+	2	−	MH121071
4C1	2002	1	Urine	+	1	−	MH121073
5C1	2002	1	Blood	+	1	−	MH121074
6C1	2008	1	Blood	+	1	−	MH121075
7C1	2008	1	Feces	+	1	−	MH121076
8C2	2009	2	URT	−	2	−	MH121077
9C2	2009	2	URT	−	2	−	MH121078
10C2	2010	2	URT	−	2	−	MH121079
11C2	2012	2	LRT	−	2	−	MH121080
12C1	2012	1	URT	−	1	−	MH121081
13C1	2012	1	Feces	+	1	−	MH121082
14C2	2012	2	Feces	+	2	−	MH121083
15C2	2012	2	Blood	+	2	−	MH121084
16C2	2012	2	Feces	−	2	−	MH121085
17C2	2013	2	LRT	−	2	−	MH121086
18C1	2013	1	Feces	+	1	−	MH121087
19C2	2013	2	Blood	+	2	−	MH121088
20C1	2013	1	Blood	+	1	−	MH121089
21C2	2014	2	Blood	+	2	−	MH121090
22C1	2014	1	URT	−	1	−	MH121091
23C2	2014	2	Blood	+	2	−	MH121092
24C2	2014	2	Blood	+	2	−	MH121093
25C5	2014	5	Feces	+	5	−	MH121094
26C2	2014	2	Feces	+	2	−	MH121095
27C2	2015	2	URT	−	2	−	MH121096
28C5	2015	5	Feces	+	5	−	MF681662
29C2	2015	2	Feces	+	2	89	MH121097
30C1	2015	1	Feces	+	1	−	MH121098
31C2	2015	2	URT	−	2	−	MH121099
32C1	2015	1	URT	−	1	−	MH121100
33C2	2015	2	Feces	+	2	−	MH121101
34C2	2015	2	Feces	+	2	−	MH121102
35C2	2015	2	Blood	+	2	−	MH121103
36C2	2015	2	Feces	+	2	−	MH121104
37C2	2015	2	Feces	+	2	−	MH121105
38C2	2016	2	Feces	+	2	−	MH121106
39C2	2016	2	Blood	+	2	−	MH121107
40C5	2015	5	Feces	+	5	−	MH121118
41C1	2016	1	Feces	+	1	−	MH121108
42C2	2017	2	Feces	+	2	−	MH121109
43C1	2017	1	Feces	+	1	−	MH121110
44C2	2017	2	Feces	+	2	−	MH121111
45C6	2017	6	Feces	+	6	−	MH121112
46C6	2017	6	Feces	+	6	−	MH121113
47C2	2017	2	Feces	+	2	89	MH121114
48C2	2017	2	Feces	+	2	−	MH121115
49C5	2017	5	Feces	+	5	−	MH121119
50C1	2017	1	Feces	+	1	−	MH121116
51C1	2017	1	URT	−	1	−	MH121117

**Figure 1:**
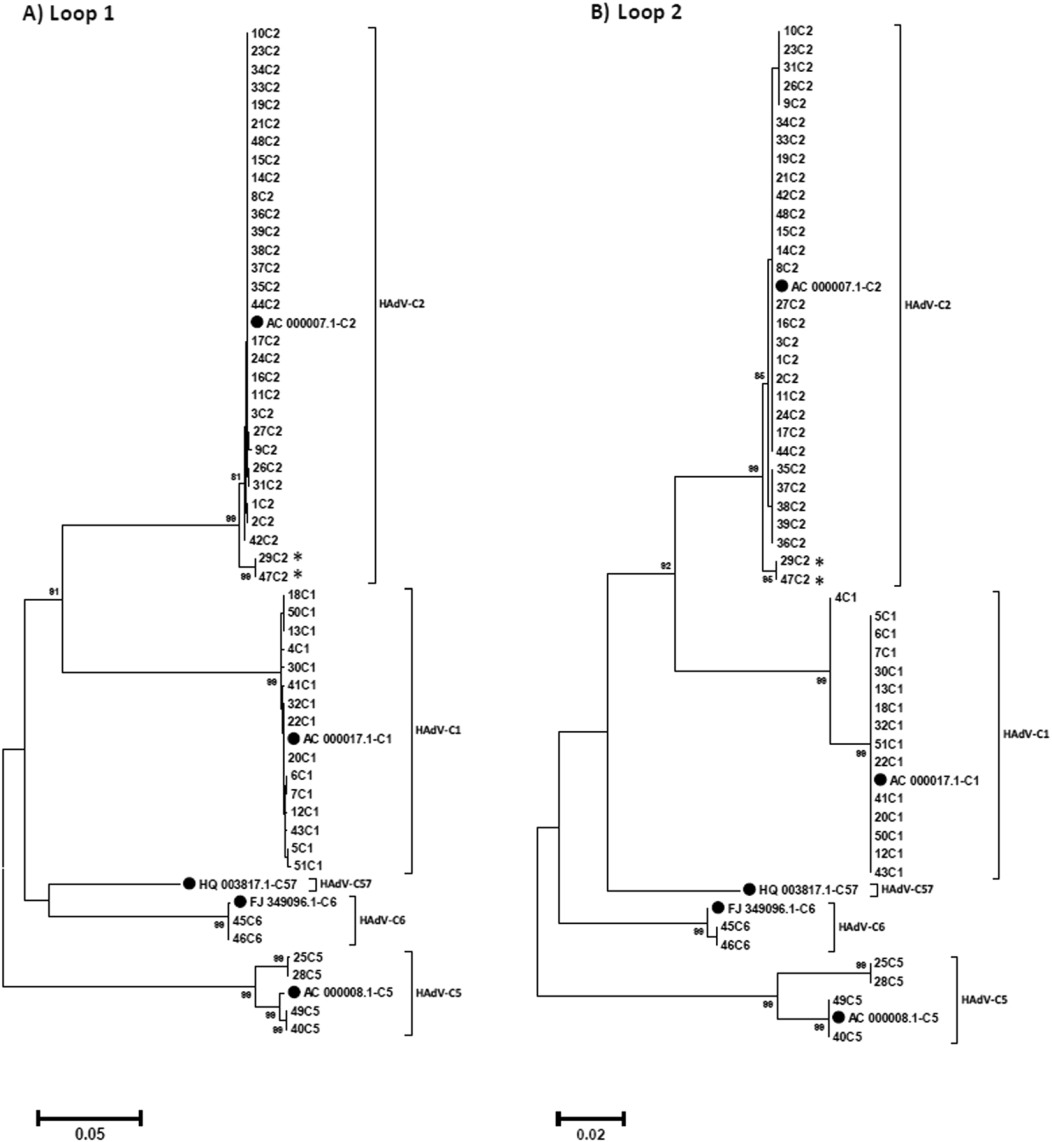
Phylogenetic analysis of the nucleic acid sequences of the neutralization ε determinant loop 1 (**A**) and loop 2 (**B**) of the hexon. Genbank sequences of the five HAdV-C prototypes are highlighted by a black dot (labelling indicates accession number-species and type). The neighbor-joining tree was generated based on the Kimura two-parameter model with MEGA7. Bootstrap values < 80% are not robust and therefore not depicted. *Strains 29C2 and 47C2 were renamed as the novel type HAdV-C89.

Nucleic acid sequences coding for the neutralization determinant of types HAdV-C1, -C2 and -C6 were highly conserved (identity 97.85–100% in loop1 and 97.89–100% in loop2, respectively), whereas these sequences were found to be more diverse in HAdV-C5 strains. In the latter, the loop 1 sequence identity was 96.20–99.12% and the loop 2 identity was 97.16–100% at the nucleotide level, at the amino acid level 97.38% – 98.44% and 97.22% to 98.76%, respectively (Fig. 1).

### Molecular phylogeny of complete genomic sequences and major capsid proteins

**Figure 2:**
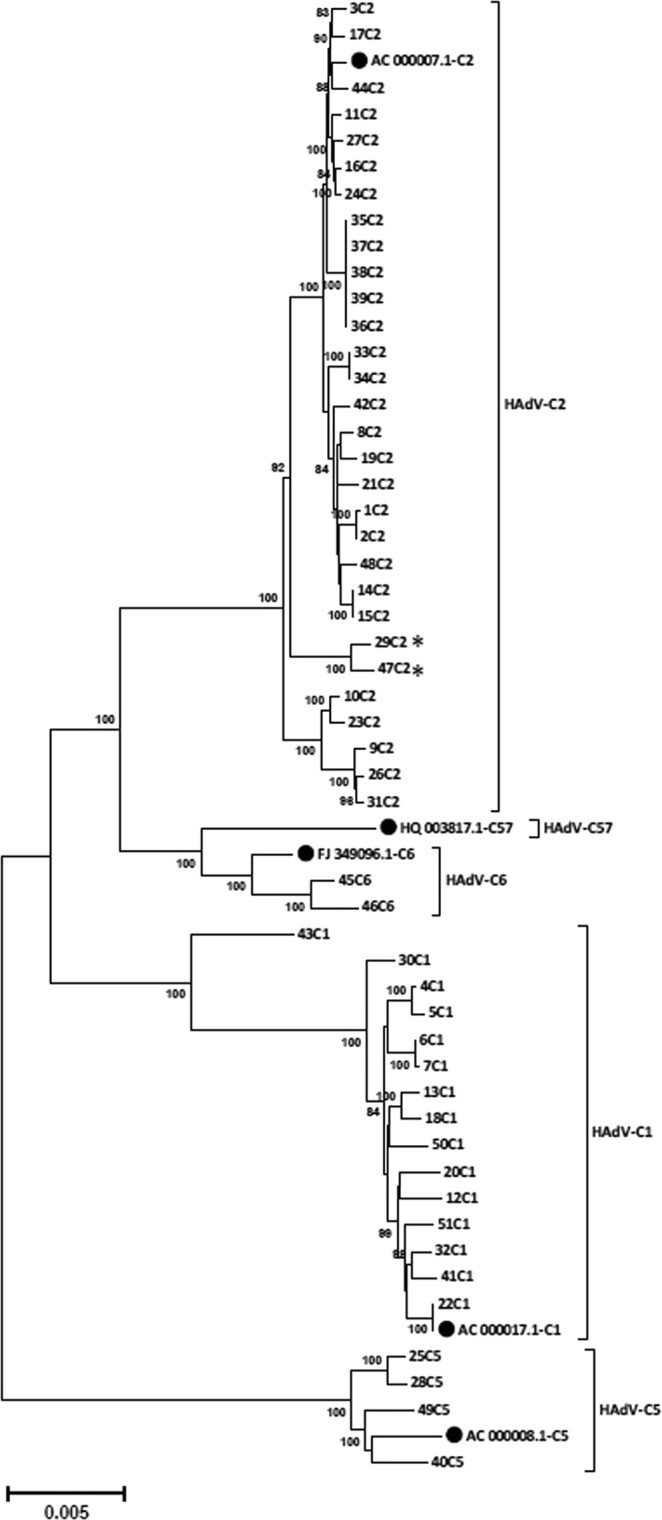
Phylogenetic analysis of whole-genome sequences. Clustering of nucleic acid sequences of circulating strains with Genbank sequences of the five species HAdV-C prototypes (highlighted by a black dot, labelling indicates accession number-species and type). The neighbor-joining tree was generated based on the Kimura two-parameter model with MEGA7. Bootstrap values < 80% are not robust and therefore not depicted. *Strains 29C2 and 47C2 were renamed as the novel type HAdV-C89.

Clustering of complete genomic sequences confirmed initial typing by imputed serology (Fig. 2).

**Figure 3:**
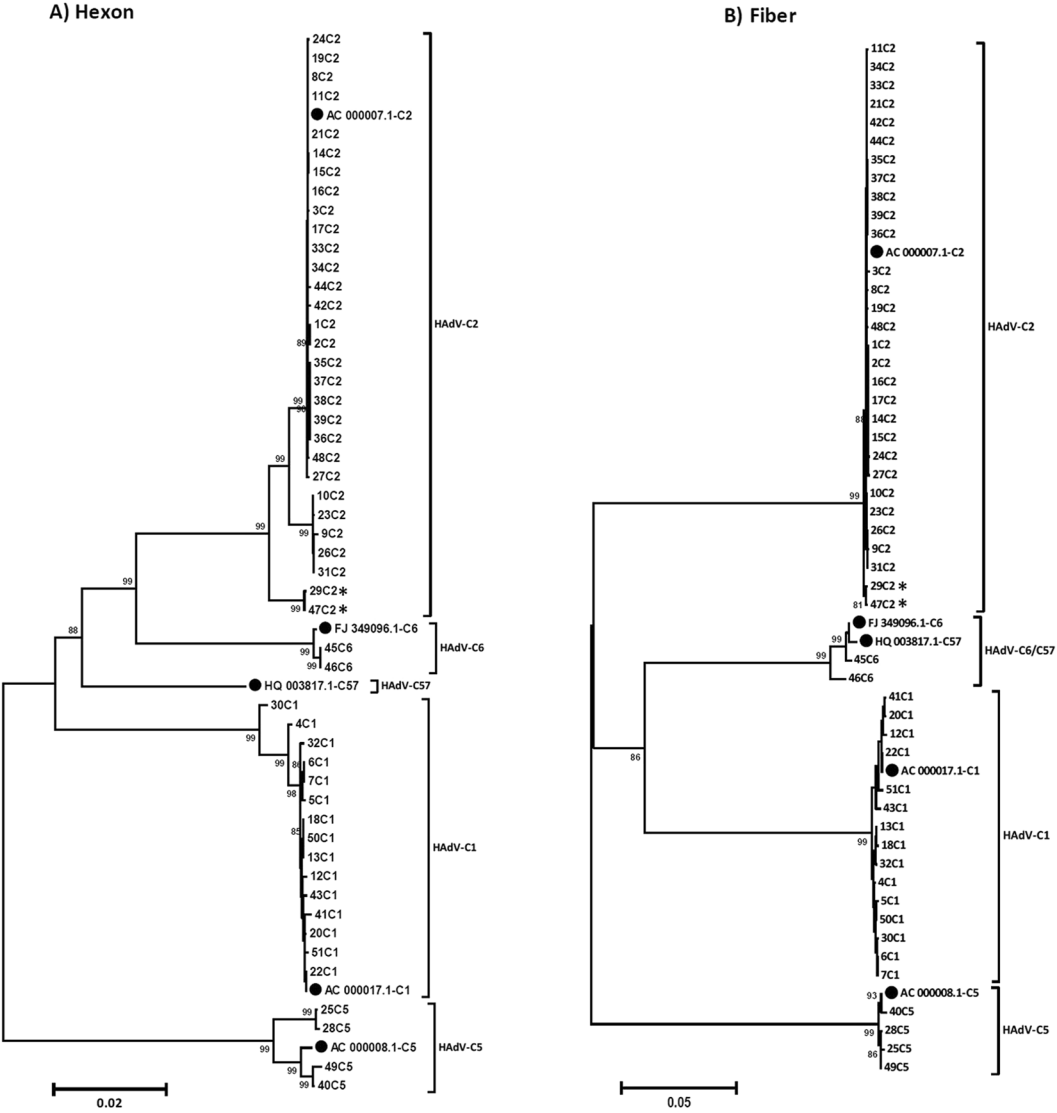
Phylogenetic analysis of the major capsid genes hexon (**A**) and fiber (**B**). Clustering of circulating strains and Genbank sequences of HAdV-C prototypes (highlighted by a black dot, labelling indicates accession number-species and type). The neighbor-joining tree was generated based on the Kimura two-parameter model with MEGA7. Bootstrap values < 80% are not robust and therefore not depicted. *Strains 29C2 and 47C2 were renamed as the novel type HAdV-C89.

In general, clustering of hexon and fiber gene sequences constantly followed clustering of complete genomic sequences, excluding intertypic recombination affecting hexon and fiber gene in the phylogeny of HAdV-C (Fig. 3).

**Figure 4:**
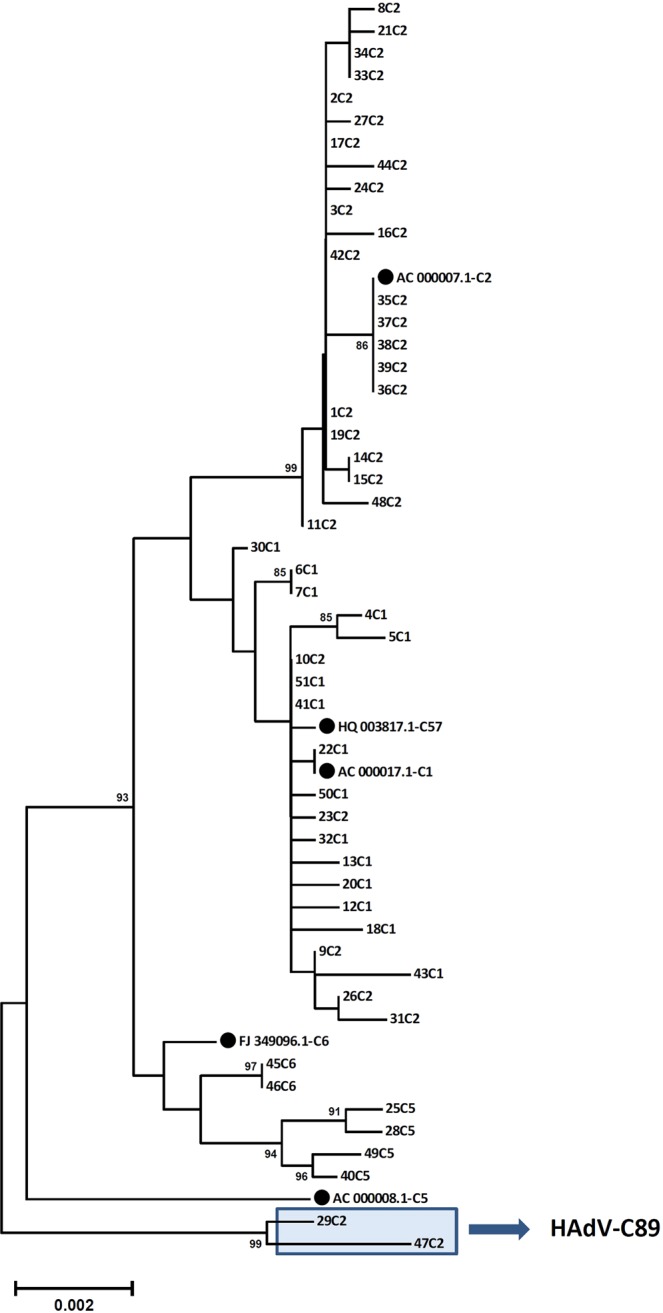
Phylogenetic analysis of the major capsid gene penton base. Clustering of circulating strains and prototype sequences (highlighted by a black dot, labelling indicates accession number-species and type). Strains 29C2 and 47C2 were highlighted with a blue box and renamed as novel type HAdV-C89. The neighbor-joining tree was generated based on the Kimura two-parameter model with MEGA7. Bootstrap values < 80% are not robust and therefore not depicted.

**Figure 5:**
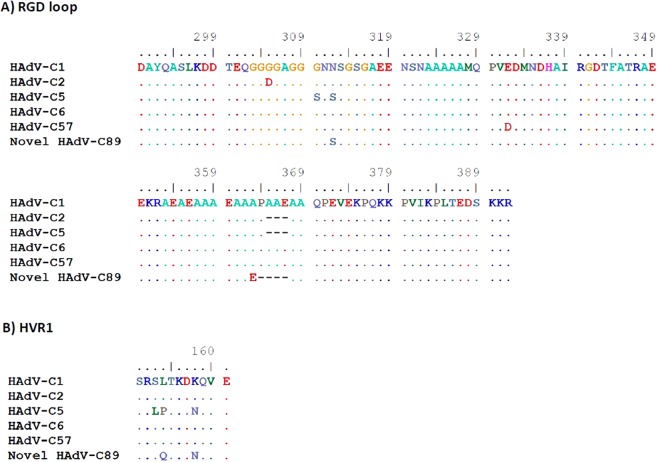
Multiple amino acid alignments of the hypervariable loops of the penton base. (**A**) RGD loop sequences and (**B**) hypervariable region 1 sequences of all HAdV-C prototypes including the novel HAdV-C89.

Two strains (29C2 and 47C2) with a genomic backbone of type HAdV-C2 had a unique penton base sequence and presented as a novel cluster in the phylogenetic tree with a bootstrap value of 99% (Fig. 4). Compared to all other HAdV-C prototype sequences strains 29C2 and 47C2 had a unique substitution (A363E) and a unique deletion of P364 in the hypervariable RGD loop of the penton base. This loop binds with its RGD amino acid motif to cellular integrins. Additionally, strains 29C2 and 47C2 had a L154Q substitution in the second hypervariable loop of the penton base compared to the species HAdV-C consensus sequence. Due to their unique penton base sequence, strains 29C2 and 47C2 were re-labelled as a novel HAdV-C type 89, with strain 29C2 as the prototype, designated as Adenovirus C human/DEU/Hannover/2015/89[P89H2F2]. Several amino acid substitutions were observed in the hypervariable region 1 (HVR1) and the hypervariable RGD loop which binds to the secondary cellular receptor in comparison to the other prototype sequences of HAdV-C (Fig. 5, for nucleotide sequences please see Supplementary Fig. S1).

Clustering of penton gene sequences suggested recombination of the penton base gene region in 9 genomes generated from other strains (9C2, 10C2, 23C2, 25C5, 26C2, 28C5, 31C2, 40C5, 49C5). Actually none of the analyzed 51 HAdV-C genomes had a penton sequence clustering with prototype 5. The only four type 5 strains (25C5, 28C5, 40C5, 49C5) found in this study were more closely related to type 6 regarding their penton sequence although this was not confirmed by high bootstrap values. Five HAdV type 2 strains (9C2, 10C2, 23C2, 26C2, 31C2) had penton sequences clustering with type 1, but also without a significant bootstrap value. As a recombinant phylogeny of the penton gene could not be confirmed by bootscanning, these 9 strains were not labelled as new genotypes. However, deduced amino acid sequences of the strains 9C2, 10C2, 23C2 and 26C2 were identical to type 1 and the deduced amino sequence of strain 31C2 had only a single amino acid substitution (Q377E) compared to the HAdV-C1 prototype sequence.

### Molecular phylogeny of early gene regions

Multiple recombination events and novel sequence stretches were observed in the E1, E2 and E4 gene regions, whereas (with the single exception of strain 43C1) clustering of the E3 sequences was consistent with typing results in imputed serology and clustering of complete genomic sequences. The E3 sequence derived from strain 43C1 clustered with type 57 in the phylogenetic tree of the E3 region (Fig. S2). Although this clustering was not supported by a significant bootstrap value, a recombinant origin of strain 43C1 was indicated by bootscanning for the 5′ part of the E3 region (about nt. position 27,500–29,000) (Fig. S3, 43C1). Bootscanning for the remaining part of the E3 gene region was not conclusive due to the closely related type 2 and type 57 prototype sequences, but multiple alignments of the deduced amino acid sequences of E3 gene products confirmed that strain 43C1 was closely related to these (or a common ancestor) but not to type HAdV-C1.

**Figure 6:**
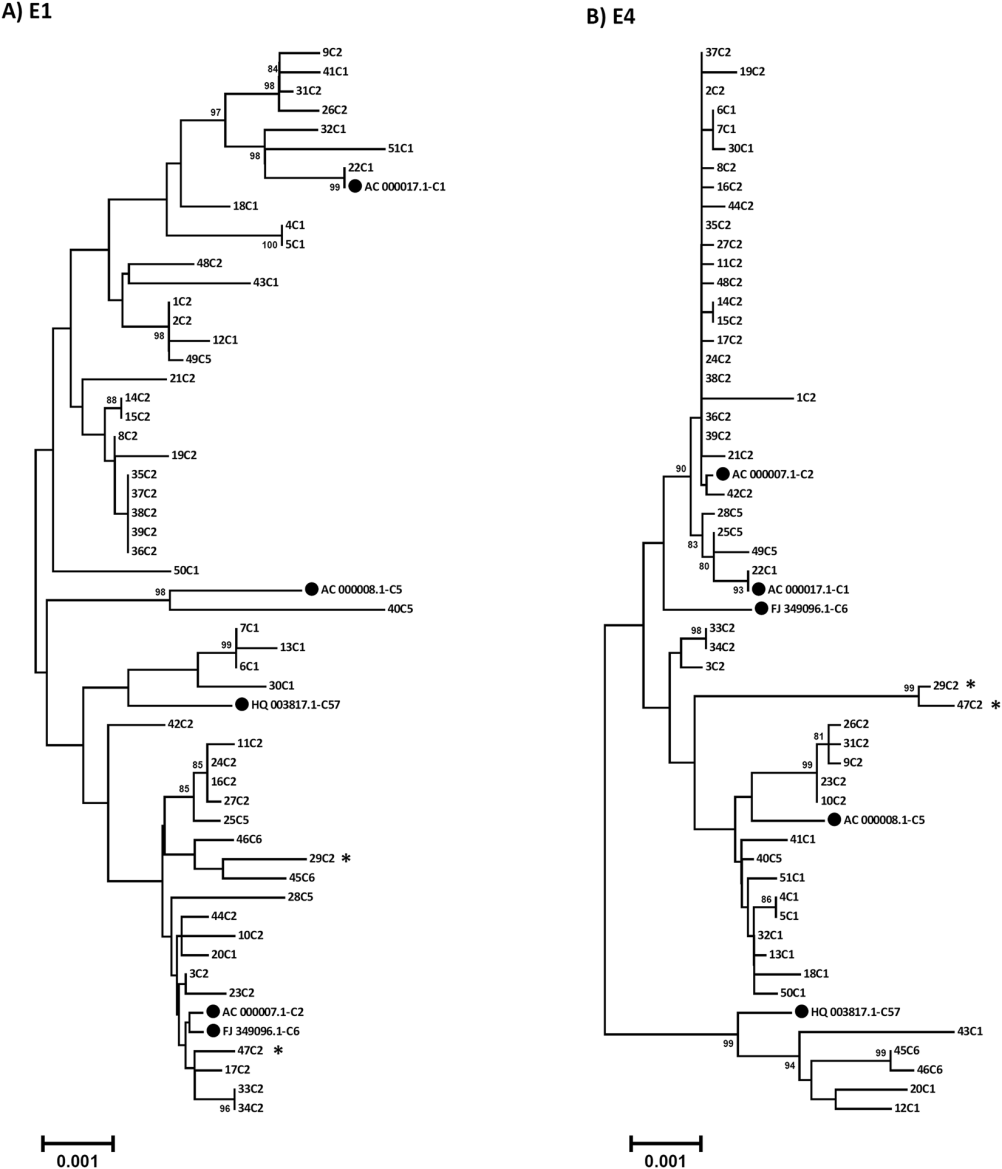
Phylogenetic analysis of the early gene regions E1 (**A**) and E4 (**B**). Clustering of circulating strains and prototype sequences (highlighted by a black dot, labelling indicates accession number-species and type). The neighbor-joining tree was generated based on the Kimura two-parameter model with MEGA7. Bootstrap values < 80% are not robust and therefore not depicted. *Strains 29C2 and 47C2 were renamed as the novel type HAdV-C89.

Surprisingly, five circulating strains (12C1, 20C1, 43C1, 45C6 and 46C6) clustered with type 57 in the E4 gene region (supported by a 100% bootstrap value and confirmed by bootscanning), although circulation of HAdV-C57 was not observed in this study. E4 sequences of multiple type 1 and type 2 strains (4C1, 5C1, 9C2, 10C2, 13C1, 18C1, 23C2, 26C2, 31C2, 32C1, 41C1, 50C1 and 51C1) clustered with type 5 (Fig. 6). This finding was not supported by a significant bootstrap value, but bootscanning of these 13 sequences confirmed a recombinant origin of the E4 sequence. Moreover, these isolates had deduced amino acid sequences identical to the sequence of prototype HAdV-C5 in E4ORF3, E4ORF4 and E4ORF6/7. Five other type 2 strains (3C2, 29C2, 33C2, 34C2, 47C2) were more distantly related to the HAdV-C5 prototype sequence, but the recombinant origin of these E4 sequences was also confirmed by bootscanning only. E4ORF3 and E4ORF4 deduced amino sequences of 3C2, 29C2, 33C2, 34C2, 47C2 were also identical to the HAdV-C5 prototype sequence but E4OR6/7 was not. Vice versa, three type 5 strains (25C5, 28C5 and 49C5) clustered with type 1 in the E4 region. Although the bootstrap value of this cluster was not significant, probably due to the very low sequence diversity between prototype 1 and prototype 2 E4 sequences, recombination was confirmed by bootscanning. Finally, three type 1 strains (6C1, 7C1 and 30C1) clustered with type 2 in the E4 region with a 92% bootstrap probability but recombination of the E4 region was not supported by bootscanning.

As gene products of E4 and E1 interact with each other, similar clustering of strains in the E1 phylogenetic tree as observed in the E4 phylogenetic tree was anticipated. Surprisingly, phylogenetic analyses of the E1 region showed a completely different clustering (Fig. 6).

Only a single sequence (strain 40C5) clustered with prototype HAdV-C5 in the E4 region and in E1 region. Three type 2 strains (9C2, 26C2, 31C2) clustered with prototype HAdV-C1 in the E1 region (supported by a 100% bootstrap value and confirmed by bootscanning) indicating an E1 sequence of recombinant origin. There were 13 other type 2 strains (1C2, 2C2, 8C2, 14C2, 15C2, 19C2, 21C2, 35C2, 36C2, 37C2, 38C2, 39C2 and 48C2) and one type 5 strain (49C5) that also clustered with type HAdV-C1 prototype sequence in the E1 gene region. Recombination was confirmed by bootscanning, but clustering in the tree was non-significant (low bootstrap value). Surprisingly, one strain (40C5) had an intra-E1 recombination event in its phylogeny indicated by bootscan analysis with the E1A region closely related to prototype HAdV-C1 and the E1B region (and most of its genomic backbone) closely related to HAdV-C5 (Fig. S3).

In the E2A region five type 2 isolates (9C2, 10C2, 23C2, 26C2, 31C2) and one type 6 strain (45C6) were found to have a recombinant phylogeny and clustering with the prototype HAdV-C1 (supported by significant bootstrap values) (Fig. S4). Moreover, the two isolates representing the novel prototype HAdV-C89 (29C2 and 47C2) had a unique E2A sequence compared to all other isolates and prototypes.

Genes of the E2B region (encoding e.g. the DNA polymerase) are highly conserved between all HAdV-C prototypes and circulating strains analyzed in this study with the exception of strains 43C1 and 50C1. These clustered on their own and had unique amino acid substitutions in comparison with all other isolates in the deduced amino acid sequence (Fig. S4).

### Molecular phylogeny of minor capsid proteins

Sequence diversity of all six minor capsid proteins (IIIa, V, VI, VII, VIII, and IX) was very low and a recombinant phylogeny could not be detected. Interestingly, two circulating type 2 strains (33C2 and 34C2, derived from the same patient) had a deletion of three amino acids (position 447 to 449) as compared to the prototype HAdV-C2 sequence in the IIIa protein (data not shown). In the minor capsid protein V a deletion of two amino acids (serine and threonine at 228–229 aa position) was detected in two other circulating strains (1C2 and 2C2).

Minor capsid proteins VII and VIII were highly conserved at the nucleotide and amino acid level. In the amino acid alignment of minor capsid protein IX, 11 out of the 30 type 2 isolates (1C2, 2C2, 8C2, 9C2, 14C2, 15C2, 19C2, 21C2, 26C2, 31C2, 42C2) were found to be more similar to prototype HAdV-C1 as they contain two amino acid substitutions (A3T and D123E) as compared to prototype HAdV-C2 protein IX amino acid sequence.

## Discussion

In the present study, analysis of the complete genomes of 51 circulating species HAdV-C strains revealed multiple recombination events of early gene regions (e.g. E1 and E4) that may contribute to the evolution of species HAdV-C. To our knowledge this has never been described before.

The E4 region encodes for six proteins (ORF1, ORF2, ORF3, ORF4, ORF6 34 K and ORF6/7), which are known to have one or more function required for lytic growth of virus, transcription of viral genes and replication^23–25^. E4 sequences of the HAdV-C1 and -C2 prototypes and many of the analyzed type 1 and 2 circulating strains were highly conserved; therefore conclusions on recombination between these two types were not feasible. In spite of this, 21 out of 45 analyzed type 1 and 2 strains displayed a recombinant E4 region. Five of these 21 strains clustered with HAdV-C57 in the E4 region and 14 of these strains had HAdV-C5 like E4 sequences, although this was confirmed by bootscanning only. Therefore, HAdV-C5 like and HAdV-C57 like E4 sequences were highly “overrepresented” in the analyzed circulating virus strains compared to their typing results in imputed serology and their genomic backbone. This indicated a positive selection for these recombinant type 1 and 2 strains that have an E4 region sequence of type 5 or 57. This positive selection was observed both in the 39 analyzed strains originating from immunosuppressed patients, where these can cause severe disseminated infections with high virus loads in peripheral blood (as e.g. strain 40C5^26^) and in the 12 other analyzed strains originating from usually mild respiratory infections of immunocompetent patients.

Although the sequence diversity between type 5 and types 1 and 2 is low in the E4 gene region, type 5 has substantial amino acid substitutions in its E4 ORFs compared to types 1 and 2. Prototypes of HAdV-C1, -C2, -C6 and many of the analyzed circulating strains shared an identical amino acid E4ORF4 sequence but prototype HAdV-C5 had a distinctive substitution of serine at position 67 that was also present in seventeen circulating strains clustering with prototype 5 in the E4 gene region. The novel prototype HAdV-C89 strains (29C2 and 47C2) also cluster with the HAdV-C5 sequence, but have three other amino acid substitutions which may affect the binding of E4ORF4 to PP2A, a key function for this E4 protein^27–30^.

The deduced amino acid sequences of the E4ORF6/7 region of prototypes HAdV-C2, -C6, -C57 and many circulating HAdV-C strains were identical. However, prototype HAdV-C5 and 13 circulating strains (4C1, 5C1, 9C2, 10C2, 13C1, 18C1, 23C2, 26C2, 31C2, 32C1, 41C1, 50C1 and 51C1) have a distinctive K at position 68 which might serve as a SUMO2/3 acceptor site in the E4orf6/7 protein, thus affecting promoter activation by E4orf6/7 or its stability (Melling, personal communication).

Moreover, the deduced amino acid sequence of the E4ORF3^31,32^ is identical in prototypes HAdV-C1, -C2, -C6 and -C57, but the prototype of HAdV-C5 has one substitution L33R that was conserved in all circulating recombinant HAdV-C strains clustering with this prototype’s E4 region sequence.

The E4ORF1 gene product binds via its C-terminal PDZ binding motif (S/T-X-¥) to proteins containing PDZ domains and as expected this motif was highly conserved in all our E4ORF1 sequences^33,34^. With exception of the C-terminal region, multiple amino acid substitutions and deletions were observed in circulating strains like 41C1 and 1C2, but will probably not affect the function of this motif. Interestingly, these mutations were only observed in the circulating virus strains whereas in all species HAdV-C prototypes the complete E4ORF1 amino acid sequence is conserved.

The overall genetic diversity of the E1 region was higher than in the E4 region and also many (17 out of 51) circulating HAdV-C strains were found to be recombinant in the E1 region (confirmed by bootscan analysis although only three of these strains clustered with significant bootstrap value). As E1 and E4 proteins interact with each other, a similar clustering of strains in the E1 region as in the E4 region could be expected, but surprisingly almost all circulating strains except 49C5 clustered differently in these phylogenetic trees. The E1 region encodes only three functional proteins (E1A, E1B-19K and E1B-55K). E1A has a conserved region 4 (CR4) at amino acid position 202–210 which mediates interactions with host cell proteins such as the corepressor CtBP^35^. Three circulating strains (43C1, 45C6, 46C6) had few substitutions in the CR4 region that might affect the interaction with host cell proteins^36^. Four strains (9C2, 26C2, 31C2 and 41C1) had a P140S substitution in the E1B-19K sequence which may affect the Bcl-2- homology region 2 (BH2) known to inhibit apoptosis by the E1B protein^37^. Apart from this single substitution the E1B-19K amino acid sequence was observed to be highly conserved. The N-terminal region of large E1B-55k protein represents an intrinsically disordered protein domain^38^ and correspondingly the sequence of this region was less conserved than the C-terminal region in the circulating strains.

The E3 region contains seven genes affecting the host immunity after virus infection but not essential for the replication of HAdV-C^39^. In our study, the E3 gene region sequences of circulating strains were highly conserved and clustered with their respective prototype sequence, with the exception of strain 43C1. This recombination is clearly evident because the E3 sequences of different HAdV-C prototypes are highly divergent. The type 1 strain 43C1 clustered with the prototype HAdV-C57 E3 region sequence (Fig. S2) and thus is the only candidate to study further the effects of a recombinant E3 region in species HAdV-C. With this single exception, clustering suggested coevolution of the E3 region with the major capsid proteins hexon and fiber in species HAdV-C, whereas in species HAdV-D, homologous recombination in the E3 region (specifically CR1 genes) has been confirmed previously^40^.

Recombination events between the genes encoding major capsid proteins (hexon, fiber and penton) are an important mechanism in the evolution of species HAdV-D types, but almost absent in the evolution of species HAdV-C strains as confirmed by our sequence data^1,5,40^. Conserved sequence segments (called “universal conserved segments”) are present in HAdV-D genomes, e.g. in the penton base gene, the hexon gene and multiple ones in the E3, fiber and E4 genes, that drive the recombination of these regions^41,42^. Obviously, this mechanism is absent in species HAdV-C. Therefore, recombination of genes encoding major capsid proteins seems to be almost impossible in species HAdV-C, as also observed in the present study. The single exception to this rule of non-recombinant evolution of major capsid proteins in species HAdV-C is type HAdV-C57. Its penton base sequence is closely related to type HAdV-C1, its fiber to type HAdV-C6 and it has a unique hexon sequence and thus a novel neutralization determinant sequence^14^. Surprisingly, all circulating strains had neutralization epitope sequences identical (or almost identical) to the prototype sequences (Fig. 1) although a rapid evolution of these loop 1 and 2 sequences could be driven by an immune escape mechanism. This was previously described in species HAdV-B^1,3,5,6^ but was obviously absent in species HAdV-C. Perhaps, E3 gene products interfering with the immune-response against HAdV-C type infected cells^39,43,44^ could be so effective that there was almost no positive selection of immune escape mutations.

In this study, another novel prototype of species HAdV-C has been described (type 89). It was typed initially as a HAdV-C2 strain by conventional methods, because both hexon and fiber sequences, as well as most of its genetic backbone are closely related to the HAdV-C2 prototype sequence. However, HAdV-C89 was found to have a novel penton base sequence. In general, penton base sequences of species HAdV-C types are far less diverse as in species HAdV-D, but interestingly the novel type HAdV-C89 penton base sequence is most diverse compared to the old prototypes in the functional RGD loop. Although amino acid residues adjacent to the RGD motif were conserved (Fig. 5), the conformation of the RGD site may be different and thus, binding properties of type 89 to the secondary cellular receptor compared to other HAdV-C types^45,46^. Moreover, HAdV-C89 has a recombinant E4 region (Fig. 6) closely related to HAdV-C5 suggesting a recombinant phylogeny of this region, which may also influence its virulence. On the other hand, prevalence of type 89 in the present study was low, representing only two out of 51 strains. These were isolated from different patients, strain 29C2 in 2015 and strain 47C2 in 2017, respectively. Prevalence of HAdV-C89 strains in this study was about equal to HAdV-C6 but, for comparison, not a single strain of HAdV-C57 was detected in the present study.

In conclusion, simple typing procedures, as merely a partial sequencing of the hexon gene of a diagnostic specimen, seem to be sufficient to describe the “core region” of the HAdV-C genome. This includes the hexon and the fibre gene and the E3 region, which was considered to be important for immune escape and probably long term persistence of HAdV-C strains after primary infection. On the other hand, these routine typing strategies will not detect novel penton base sequences and thus would have missed the novel type 89. Moreover, future studies employing complete genomic sequencing on circulating HAdV-C strains are needed to study the clinical significance of recombination in the E1 and E4 regions as e.g. the predominance of type 5 derived E4 regions in the present study.

## Methods

### Specimens and strains

A total of 51 species HAdV-C strains were obtained between the years 2000 and 2017 from clinical specimens originating from the upper respiratory tract (URT, n = 9), lower respiratory tract (LRT, n = 2), feces (n = 27), blood (n = 12) and urine (n = 1). For 11 of these specimens adenoviruses were isolated on A549 cell cultures prior to sequencing, direct sequencing was applied to the other specimens (Table 1). Specimens originated from 40 patients; strains 6C1, 7C1 and 30C1 originated from a single patient as did strains 35C2, 36 C2, 37C2, 38 C2 and 39C2 from another patient. Strains 1C2 and 2C2 also originated from a single patient, as did strains 4C1 and 5C1, 14C2 and 15 C2, 33 C2 and 34 C2, 45C6 and 46C6. Specimens originated from different regions of Germany. Table 1 shows an overview of specimens sequenced, year of isolation and Genbank Accession Number.

### Nucleic acid extraction and viral load quantitation

Extraction of DNA from 200 µl clinical material or cell culture supernatant was performed as described by QIAamp DNA Blood Mini Kit (Qiagen, Hilden, Germany). HAdV DNA was quantified by a generic, quantitative real-time PCR as described previously^47^.

### Imputed serology by partial sequencing

Initial typing was performed by Sanger sequencing of parts of the neutralization epitope (loop 1 and loop 2) of the hexon gene (imputed serology) and the fibre knob gene as previously described^48^.

### Enrichment of HAdV DNA and library preparation

Library preparation of 42 specimens (including all cell culture isolates) with sufficient HAdV DNA load (a ratio of HAdV/human genomic DNA >1,000) was performed using Nextera XT DNA library preparation kit (Illumina, San Diego, CA, USA) with 1 ng of DNA as input following manufacturers’ guideline. Purified DNA of the other nine specimens (6C1, 19C2, 20C1, 23C2, 24C2, 35C2, 47C2, 48C2 and 50C1) was sheared by sonication, and sequencing libraries were prepared directly (KAPA library preparation kit, KAPA Biosystems, Wilmington, MA, USA) as previously described^49^, with a few modifications. DNA fragments were end-repaired, A-tailed and ligated to NEBnext Illumina adapter (New England Biolabs, Ipswich, MA, USA). After PCR pre-amplification (6–14 cycles) with adapter specific primers, up to 750 ng of DNA was target enriched for HAdV fragments with RNA baits. These 120-mer RNA baits were designed to span the length of the positive strand of 24 Genbank HAdV-C1, -C2 and -C5 reference genomes (SureSelectXT kit, Agilent Technologies, CA, USA).

### Next generation sequencing and bioinformatics

After quality control, libraries were sequenced on an Illumina MiSeq platform (2*300 bp paired-ends run, v3 sequencing chemistry). The obtained sequence data was quality controlled and de novo assembled using CLC Genomics Workbench v9 (Qiagen, https://www.qiagenbioinformatics.com/products/clc-genomics-workbench/ and SPAdes v3.6^50^ with minimum average coverage of 22 and maximum average coverage 78111 per nucleotide. Genome annotations were transferred from the most closely related type and cross-checked by using an ORF finder followed by BLAST analysis using Geneious v10 (https://www.geneious.com/).

### Phylogenetic and recombination analysis

A multiple alignment of whole genomic sequences was constructed using fast Fourier transforms (MAFFT)^51^. This software (https://www.ebi.ac.uk/Tools/msa/mafft/) was applied using the default gap parameters. Pairwise alignment, comparisons and visualization of genomes were performed on BioEdit version 7.2.0 (http://www.mbio.ncsu.edu/BioEdit/page2.html). Bootstrapped, neighbor-joining (Kimura 2-parameter model) phylogenetic trees with 1000 replicates were constructed using MEGA v7 software. Similarity plots and recombination detection (bootscan approach) was performed using Simplot software (version 3.5.1) with a window size of 1000 bp and step size of 200bp^52^. The following complete genomic nucleotide sequences representing all prototypes of the HAdV-C species were used in the analysis (Genbank accession numbers in parentheses): HAdV-C1 (AC000017), HAdV-C2 (AC000007), HAdV-C5 (AC000008), HAdV-C6 (FJ349096) and HAdV-C57 (HQ003817). Furthermore, amino acid alignments for each gene were constructed with ClustalW program.

### Ethical statement

All methods were carried out in accordance with relevant guidelines and regulations. For scientific use of routine anonymized data, ethical approval is not required in Germany (confirmed by a letter of the local ethical committee “Ethikkommission der Medizinischen Hochschule Hannover”, 9th of April, 2018). This study analyzed molecular phylogeny of the species human adenovirus C and did not include experimentation with human tissue, therefore informed consent of patients is not required for this type of study.

## Supplementary information

Supplementary Dataset 1

## References

[1] Robinson CM (2013). Molecular evolution of human adenoviruses. Sci Rep.

[2] Lion T (2014). Adenovirus infections in immunocompetent and immunocompromised patients. Clinical microbiology reviews.

[3] Hashimoto S (2018). Recombinant type Human mastadenovirus D85 associated with epidemic keratoconjunctivitis since 2015 in Japan. Journal of medical virology.

[4] Seto D, Chodosh J, Brister JR, Jones MS (2011). & Members of the Adenovirus Research, C. Using the whole-genome sequence to characterize and name human adenoviruses. Journal of virology.

[5] Robinson CM, Seto D, Jones MS, Dyer DW, Chodosh J (2011). Molecular evolution of human species D adenoviruses. Infect Genet Evol.

[6] Ismail AM (2018). Genomic analysis of a large set of currently-and historically-important human adenovirus pathogens. Emerg Microbes Infect.

[7] Yoshitomi H, Sera N, Gonzalez G, Hanaoka N, Fujimoto T (2017). First isolation of a new type of human adenovirus (genotype 79), species Human mastadenovirus B (B2) from sewage water in Japan. Journal of medical virology.

[8] Walsh MP (2009). Evidence of molecular evolution driven by recombination events influencing tropism in a novel human adenovirus that causes epidemic keratoconjunctivitis. PLoS One.

[9] Dehghan S (2013). Computational analysis of four human adenovirus type 4 genomes reveals molecular evolution through two interspecies recombination events. Virology.

[10] Garnett CT, Erdman D, Xu W, Gooding LR (2002). Prevalence and quantitation of species C adenovirus DNA in human mucosal lymphocytes. Journal of virology.

[11] Lion T (2010). Monitoring of adenovirus load in stool by real-time PCR permits early detection of impending invasive infection in patients after allogeneic stem cell transplantation. Leukemia.

[12] Al Qurashi YM, Guiver M, Cooper RJ (2011). Sequence typing of adenovirus from samples from hematological stem cell transplant recipients. Journal of medical virology.

[13] Leen AM, Rooney CM (2005). Adenovirus as an emerging pathogen in immunocompromised patients. British journal of haematology.

[14] Walsh MP (2011). Computational analysis of two species C human adenoviruses provides evidence of a novel virus. J Clin Microbiol.

[15] Ison MG (2006). Adenovirus infections in transplant recipients. Clinical infectious diseases: an official publication of the Infectious Diseases Society of America.

[16] Hong JY (2001). Lower respiratory tract infections due to adenovirus in hospitalized Korean children: epidemiology, clinical features, and prognosis. Clinical infectious diseases: an official publication of the Infectious Diseases Society of America.

[17] Rocholl C, Gerber K, Daly J, Pavia AT, Byington CL (2004). Adenoviral infections in children: the impact of rapid diagnosis. Pediatrics.

[18] Garnett CT (2009). Latent species C adenoviruses in human tonsil tissues. Journal of virology.

[19] Kosulin K (2016). Persistence and reactivation of human adenoviruses in the gastrointestinal tract. Clin Microbiol Infect.

[20] Veltrop-Duits LA (2011). High titers of pre-existing adenovirus serotype-specific neutralizing antibodies in the host predict viral reactivation after allogeneic stem cell transplantation in children. Clinical infectious diseases: an official publication of the Infectious Diseases Society of America.

[21] Ganzenmueller T, Heim A (2012). Adenoviral load diagnostics by quantitative polymerase chain reaction: techniques and application. Rev Med Virol.

[22] Houldcroft, C. J. *et al*. Use of Whole-genome Sequencing of Adenovirus in Immunocompromised Paediatric Patients to Identify Nosocomial Transmission and Mixed-genotype Infection. *J Infect Dis*, 10.1093/infdis/jiy323 (2018). 10.1093/infdis/jiy323 29917114

[23] Tauber B, Dobner T (2001). Adenovirus early E4 genes in viral oncogenesis. Oncogene.

[24] Tauber B, Dobner T (2001). Molecular regulation and biological function of adenovirus early genes: the E4 ORFs. Gene.

[25] Leppard KN (1997). E4 gene function in adenovirus, adenovirus vector and adeno-associated virus infections. The Journal of general virology.

[26] Voigt S, Hofmann J, Edelmann A, Sauerbrei A, Kuhl JS (2016). Brincidofovir clearance of acyclovir-resistant herpes simplex virus-1 and adenovirus infection after stem cell transplantation. Transpl Infect Dis.

[27] Shtrichman R, Sharf R, Barr H, Dobner T, Kleinberger T (1999). Induction of apoptosis by adenovirus E4orf4 protein is specific to transformed cells and requires an interaction with protein phosphatase 2A. Proceedings of the National Academy of Sciences of the United States of America.

[28] Miron MJ (2009). Localization and importance of the adenovirus E4orf4 protein during lytic infection. Journal of virology.

[29] Mui MZ (2013). Identification of the adenovirus E4orf4 protein binding site on the B55alpha and Cdc55 regulatory subunits of PP2A: Implications for PP2A function, tumor cell killing and viral replication. PLoS pathogens.

[30] Kleinberger T, Shenk T (1993). Adenovirus E4orf4 protein binds to protein phosphatase 2A, and the complex down regulates E1A-enhanced junB transcription. Journal of virology.

[31] Forrester NA (2012). Adenovirus E4orf3 targets transcriptional intermediary factor 1gamma for proteasome-dependent degradation during infection. Journal of virology.

[32] Vink EI (2015). Impact of Adenovirus E4-ORF3 Oligomerization and Protein Localization on Cellular Gene Expression. Viruses.

[33] James, C. D. & Roberts, S. Viral Interactions with PDZ Domain-Containing Proteins-An Oncogenic Trait? *Pathogens* **5**, 10.3390/pathogens5010008 (2016). 10.3390/pathogens5010008 PMC4810129 26797638

[34] Chung SH, Frese KK, Weiss RS, Prasad BV, Javier RT (2007). A new crucial protein interaction element that targets the adenovirus E4-ORF1 oncoprotein to membrane vesicles. Journal of virology.

[35] Zhang Q, Yao H, Vo N, Goodman RH (2000). Acetylation of adenovirus E1A regulates binding of the transcriptional corepressor CtBP. Proceedings of the National Academy of Sciences of the United States of America.

[36] Cohen MJ (2013). Dissection of the C-terminal region of E1A redefines the roles of CtBP and other cellular targets in oncogenic transformation. Journal of virology.

[37] Han J, Sabbatini P, White E (1996). Induction of apoptosis by human Nbk/Bik, a BH3-containing protein that interacts with E1B 19K. Molecular and cellular biology.

[38] Sieber T (2011). Intrinsic disorder in the common N-terminus of human adenovirus 5 E1B-55K and its related E1BN proteins indicated by studies on E1B-93R. Virology.

[39] Lichtenstein DL, Toth K, Doronin K, Tollefson AE, Wold WS (2004). Functions and mechanisms of action of the adenovirus E3 proteins. International reviews of immunology.

[40] Singh G (2013). Homologous recombination in E3 genes of human adenovirus species D. Journal of virology.

[41] Gonzalez G (2014). Intertypic modular exchanges of genomic segments by homologous recombination at universally conserved segments in human adenovirus species D. Gene.

[42] Gonzalez G, Koyanagi KO, Aoki K, Watanabe H (2015). Interregional Coevolution Analysis Revealing Functional and Structural Interrelatedness between Different Genomic Regions in Human Mastadenovirus D. Journal of virology.

[43] Burgert HG (2002). Subversion of host defense mechanisms by adenoviruses. Curr Top Microbiol Immunol.

[44] Windheim M (2013). A unique secreted adenovirus E3 protein binds to the leukocyte common antigen CD45 and modulates leukocyte functions. Proceedings of the National Academy of Sciences of the United States of America.

[45] Ruoslahti E (1996). RGD and other recognition sequences for integrins. Annu Rev Cell Dev Biol.

[46] Madisch I (2007). Phylogenetic analysis and structural predictions of human adenovirus penton proteins as a basis for tissue-specific adenovirus vector design. Journal of virology.

[47] Heim A, Ebnet C, Harste G, Pring-Akerblom P (2003). Rapid and quantitative detection of human adenovirus DNA by real-time PCR. Journal of medical virology.

[48] Madisch I, Harste G, Pommer H, Heim A (2005). Phylogenetic analysis of the main neutralization and hemagglutination determinants of all human adenovirus prototypes as a basis for molecular classification and taxonomy. Journal of virology.

[49] Wilkie GS (2014). First fatality associated with elephant endotheliotropic herpesvirus 5 in an Asian elephant: pathological findings and complete viral genome sequence. Sci Rep.

[50] Nurk S (2013). Assembling single-cell genomes and mini-metagenomes from chimeric MDA products. Journal of computational biology: a journal of computational molecular cell biology.

[51] Katoh K, Toh H (2008). Recent developments in the MAFFT multiple sequence alignment program. Brief Bioinform.

[52] Lole KS (1999). Full-length human immunodeficiency virus type 1 genomes from subtype C-infected seroconverters in India, with evidence of intersubtype recombination. Journal of virology.

