# Supplementary material for: Molecular Evolution of Human Adenovirus (HAdV) Species C
Source: Sci Rep. 2019 Jan 31;9:1039. doi: 10.1038/s41598-018-37249-4 (PMC6355881; doi:10.1038/s41598-018-37249-4)

Molecular Evolution of Human Adenovirus (HAdV) Species C

Akshay Dhingra[1,6], Elias Hage[1,6], Tina Ganzenmueller[1,6], Sindy Böttcher[2]., Jörg Hofmann[3], Klaus Hamprecht[4], Patrick Obermeier[2,7,8], Barbara Rath[7,8], Fabian Hausmann[5], Thomas Dobner[5], Albert Heim[1,6*]

[1]Hannover Medical School, Institute of Virology, Hannover, Germany

[2]Robert Koch Institut, FG 15, Nationales Referenzzentrum für Poliomyelitis und Enteroviren, Berlin, Germany

[3]Institute of Medical Virology, Helmut-Ruska-Haus, Charité Medical School, Berlin, Germany

[4]Institut für Medizinische Virologie und Epidemiologie der Viruserkrankungen, Universitätsklinikum Tübingen, Tübingen, Germany

[5]Heinrich Pette Institute, Leibniz Institute for Experimental Virology, Hamburg, Germany

[6]German Centre for Infection Research (DZIF), partner site Hannover-Braunschweig, Germany

[7]Vienna Vaccine Safety Initiative, Berlin, Germany

[8]Laboratoire Chrono-environnement, Université Bourgogne Franche-Comté, Besançon, France

*Corresponding author: heim.albert@mh-hannover.de

A) RGD loop

```
                          10         20         30         40         50         60
                  ....|....| ....|....| ....|....| ....|....| ....|....| ....|....|
HAdV-C1           gatgtggacg cctaccaggc aagcttgaaa gatgacaccg aacagggcgg gggtggcgca
HAdV-C2           .......... .......... ......a... .......... .......... ..a.......
HAdV-C5           .......... .......... g......... .......... .......... ..........
HAdV-C6           .......... .......... .......... .......... .......... ..........
HAdV-C57          .......... .......... .......... .......... .......... ..........
Novel HAdV-C89    .......... .......... g......... .......... .......... ..........

                          70         80         90        100        110        120
                  ....|....| ....|....| ....|....| ....|....| ....|....| ....|....|
HAdV-C1           ggcggcggca acaacagtgg cagcggcgcg gaagagaact ccaacgcggc agccgcggca
HAdV-C2           .......... .......... .......... .......... .......... ..........
HAdV-C5           ......a... ...g...... .......... .......... .......... ..........
HAdV-C6           .......... .......... .......... .......... .......... ...t......
HAdV-C57          .......... .......... .......... .......... .......... ..........
Novel HAdV-C89    .......... ...g...... .......... .......... .......... ..........

                         130        140        150        160        170        180
                  ....|....| ....|....| ....|....| ....|....| ....|....| ....|....|
HAdV-C1           atgcagccgg tggaggacat gaacgatcat gccattcgcg gcgacacctt tgccacacgg
HAdV-C2           .......... .......... .......... .......... .......... ..........
HAdV-C5           .......... .......... .......... .......... .......... ..........
HAdV-C6           .......... .......... .......... .......... .......... ..........
HAdV-C57          .......... ....t..... .......... .......... .......... ..........
Novel HAdV-C89    .......... .......... .......... .......... .......... ..........

                         190        200        210        220        230        240
                  ....|....| ....|....| ....|....| ....|....| ....|....| ....|....|
HAdV-C1           gcggaggaga agcgcgctga ggccgaggca gcggccgaag ctgccgcccc cgctgcggag
HAdV-C2           .......... .......... .......... .....a.... ........-- -------ccc
HAdV-C5           ..t....... .......... ......a... .......... .......... .---------
HAdV-C6           .......... .......... .......... .......... .......... ..........
HAdV-C57          .......... .......... .......... .......... .......... ..........
Novel HAdV-C89    ..t....... .......... ......a... .......... ....g.---- --------..

                         250        260        270        280        290        300
                  ....|....| ....|....| ....|....| ....|....| ....|....| ....|....|
HAdV-C1           gctgcacaac ccgaggtcga gaagcctcag aagaaaccgg tgattaaacc cctgacagag
HAdV-C2           .....g.... .......... .......... .......... ....c..... ..........
HAdV-C5           .....g.... .......... .......... .......... ....c..... ..........
HAdV-C6           .......... .......... .......... .......... .......... ..........
HAdV-C57          .......... .......... .......... .......... .......... ..........
Novel HAdV-C89    .....g.... .......... .......... .......... ....c..... ..........

                         310
                  ....|....| ....|
HAdV-C1           gacagcaaga aacgc
HAdV-C2           .......... .....
HAdV-C5           .......... .....
HAdV-C6           .......... .....
HAdV-C57          .......... .....
Novel HAdV-C89    .......... .....
```

B) HVR1

```
                          10         20         30
                  ....|....| ....|....| ....|....| ...
HAdV-C1           tcgcgctcgc ttactaagga caaacaggtg gag
HAdV-C2           .......... .......... .......... ...
HAdV-C5           .......t.. c......... ...t...... ...
HAdV-C6           .......... .......... .......... ...
HAdV-C57          .......... .......... .......... ...
Novel HAdV-C89    .......... ag..c..a.. ...t...... ...
```

Figure S1: Multiple nucleic acid alignments A) RGD loop sequences and B) hypervariable region 1 sequences of all HAdV-C prototypes including the novel HAdV-C89.

Figure S2: Phylogenetic analysis of the E3 gene region. Clustering of circulating strains and prototype sequences (highlighted by a black dot, labelling indicates accession number-species and type). The neighbor-joining tree was generated based on the Kimura two-parameter model with MEGA7. Bootstrap value <80% are not robust and therefore not depicted. * Strains 29C2 and 47C2 were renamed as the novel type HAdV-C89.

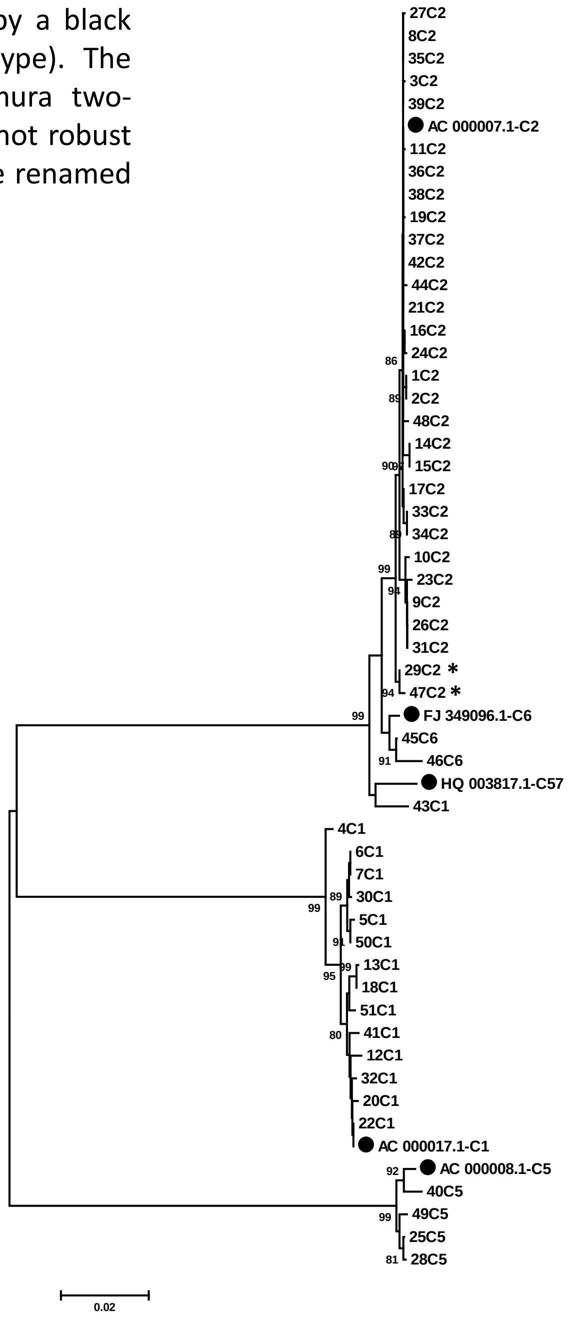

Figure S3: Bootscan analysis of fifty one circulating strains included in this study with the prototype HAdV-C sequences. Bootscan plots comparing each circulating strain with species HAdV-C prototype sequences. On top of each bootscan plot the gene organisation of HAdV-C is indicated. X-axis represent position of the genome and Y-axis show % of permuted trees. Bootscan Parameters are indicated in italics at the bottom of each plot.

1C2

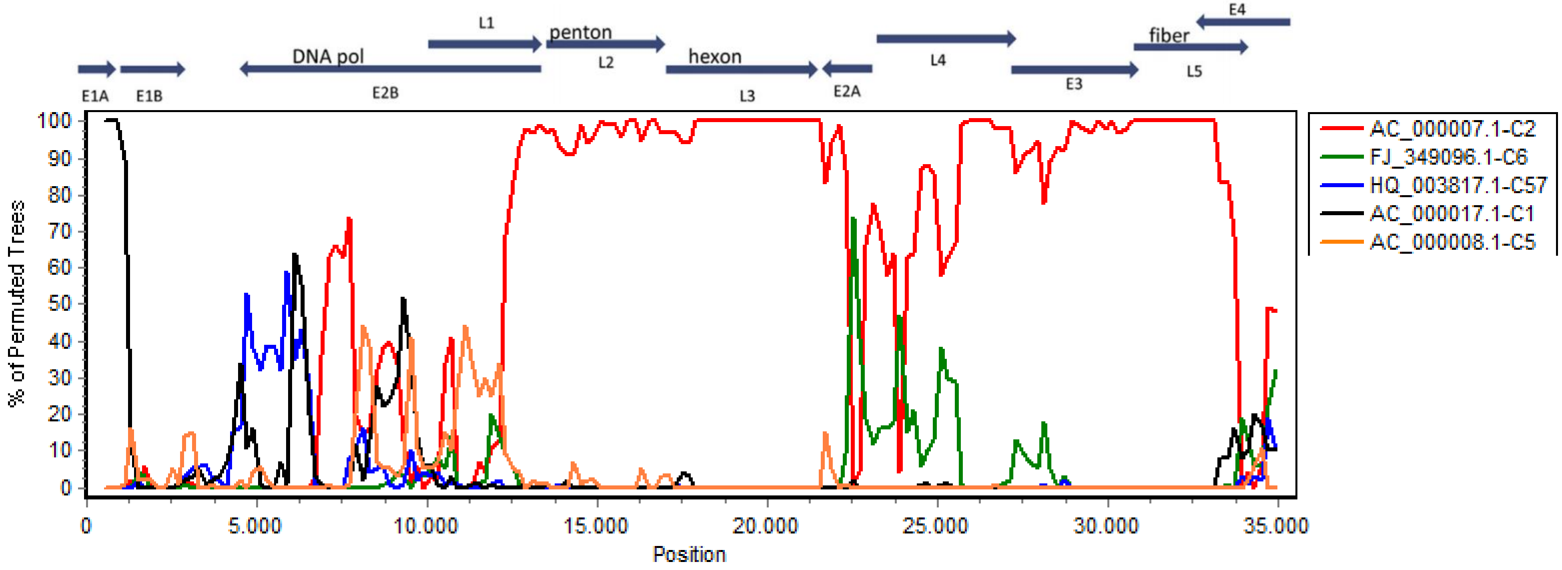

Window: 1000 bp, Step: 200 bp, GapStrip: On, Reps: 100, Kimura (2-parameter), T/t: 2,0, Neighbor-Joining

2C2

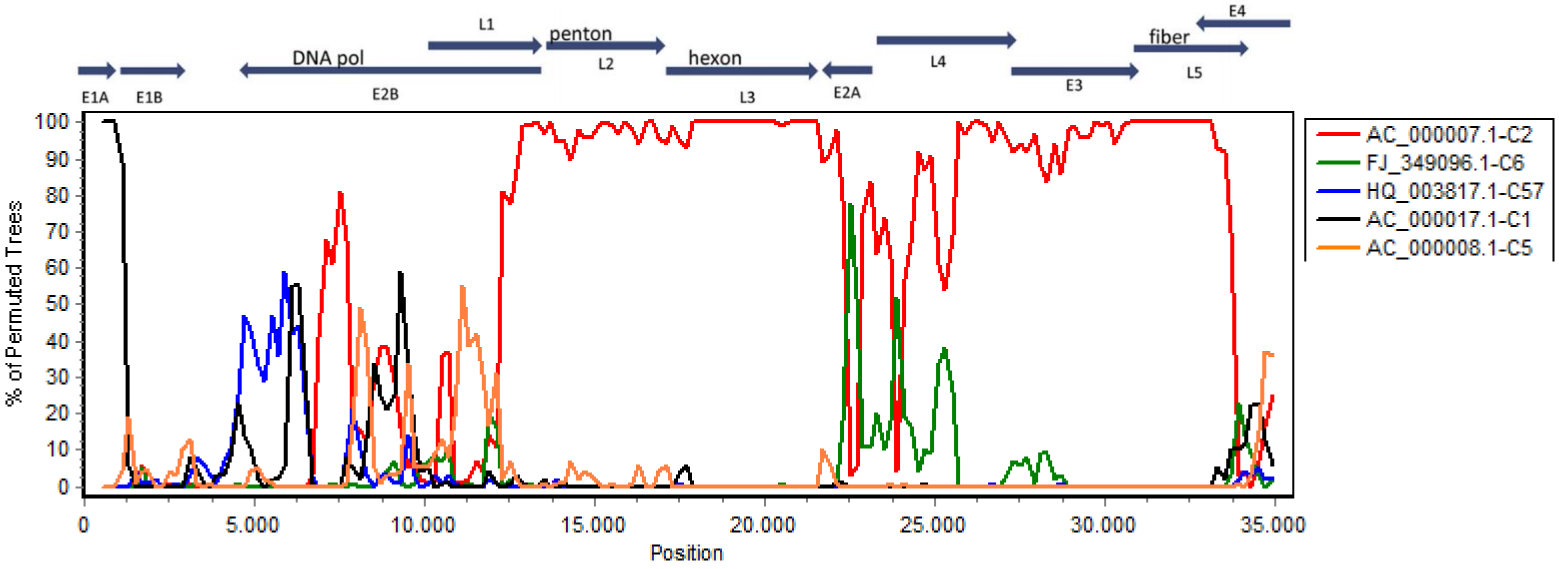

Window: 1000 bp, Step: 200 bp, GapStrip: On, Reps: 100, Kimura (2-parameter), T/t: 2,0, Neighbor-Joining

Figure S2

3C2

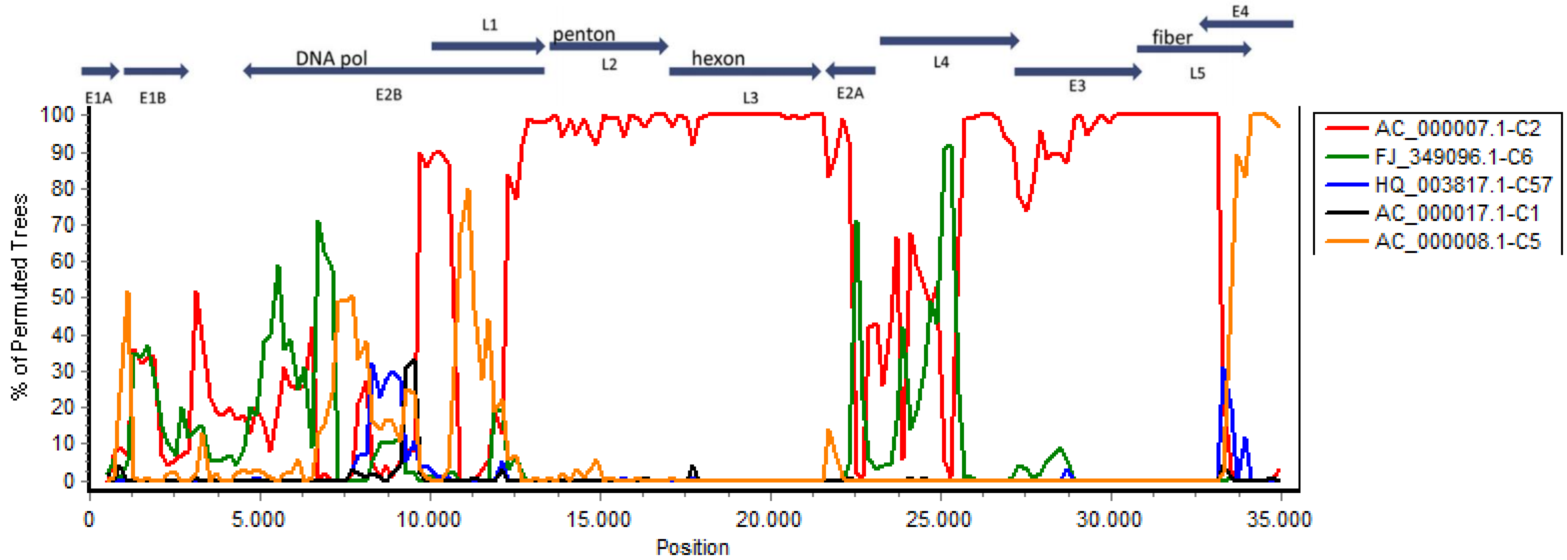

Window: 1000 bp, Step: 200 bp, GapStrip: On, Reps: 100, Kimura (2-parameter), T/t: 2,0, Neighbor-Joining

4C1

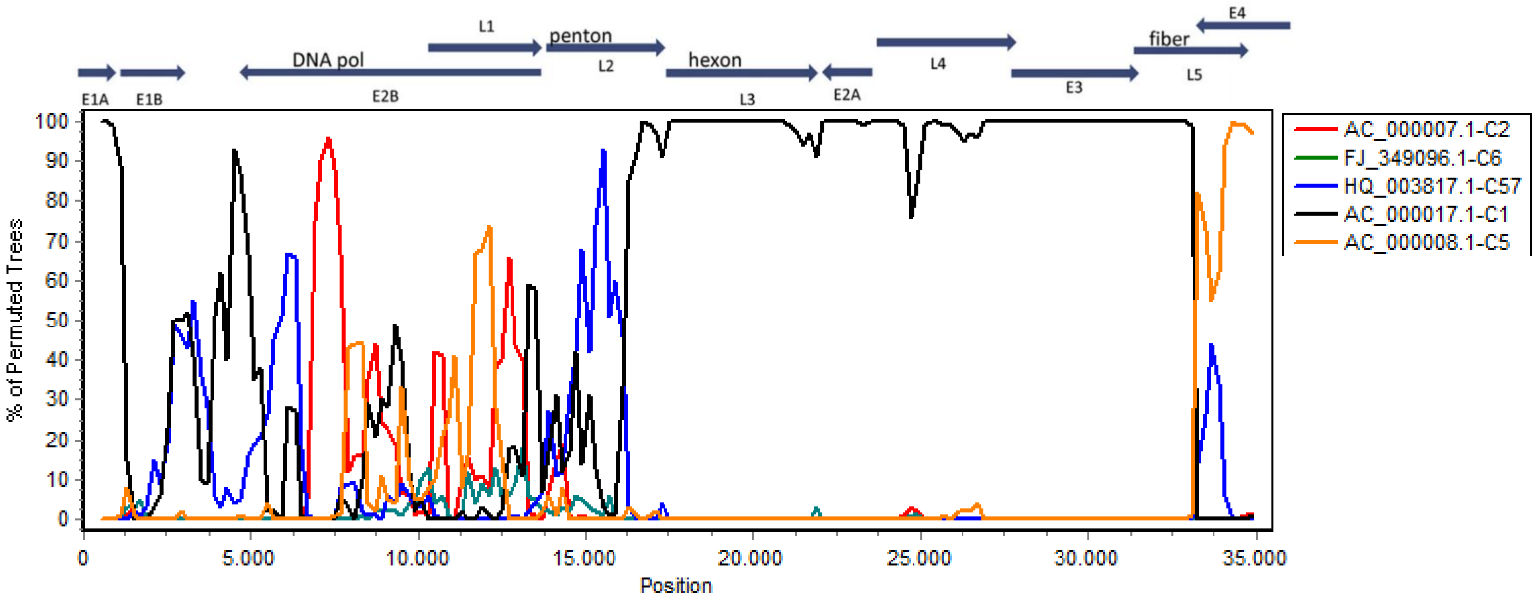

Window: 1000 bp, Step: 200 bp, GapStrip: On, Reps: 100, Kimura (2-parameter), T/t: 2,0, Neighbor-Joining

Figure S2

5C1

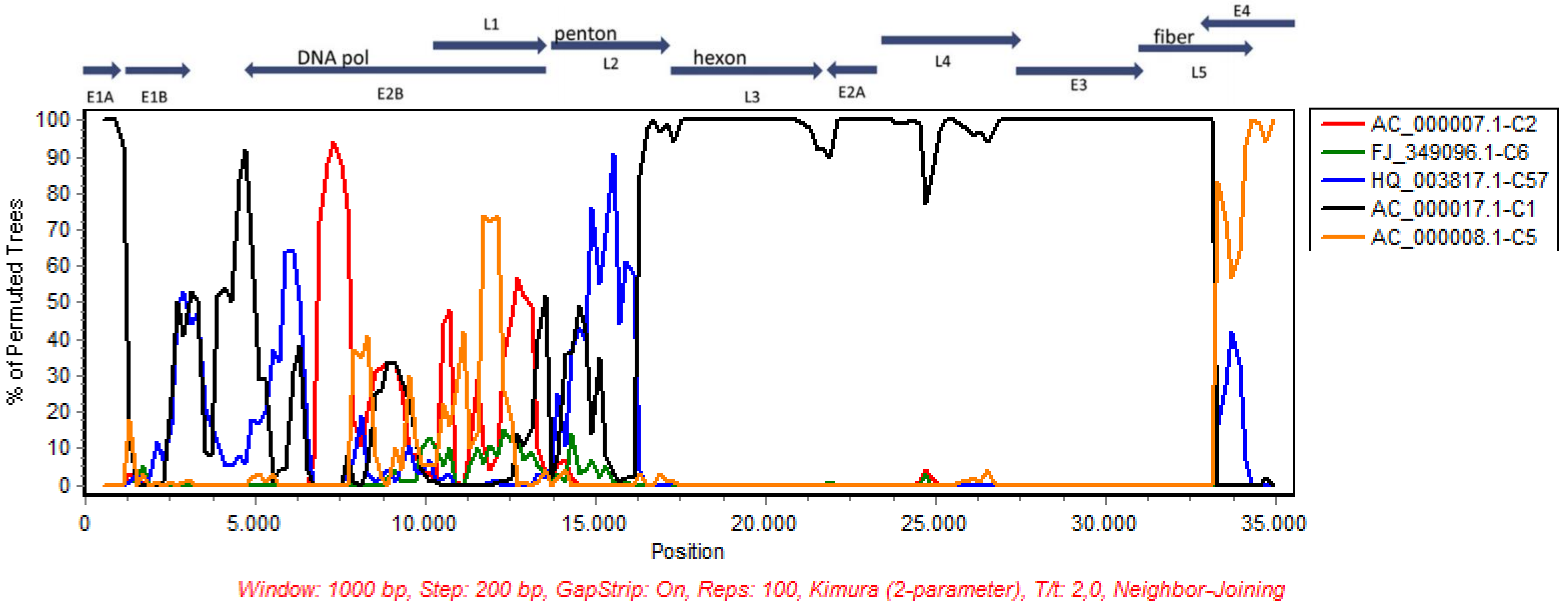

6C1

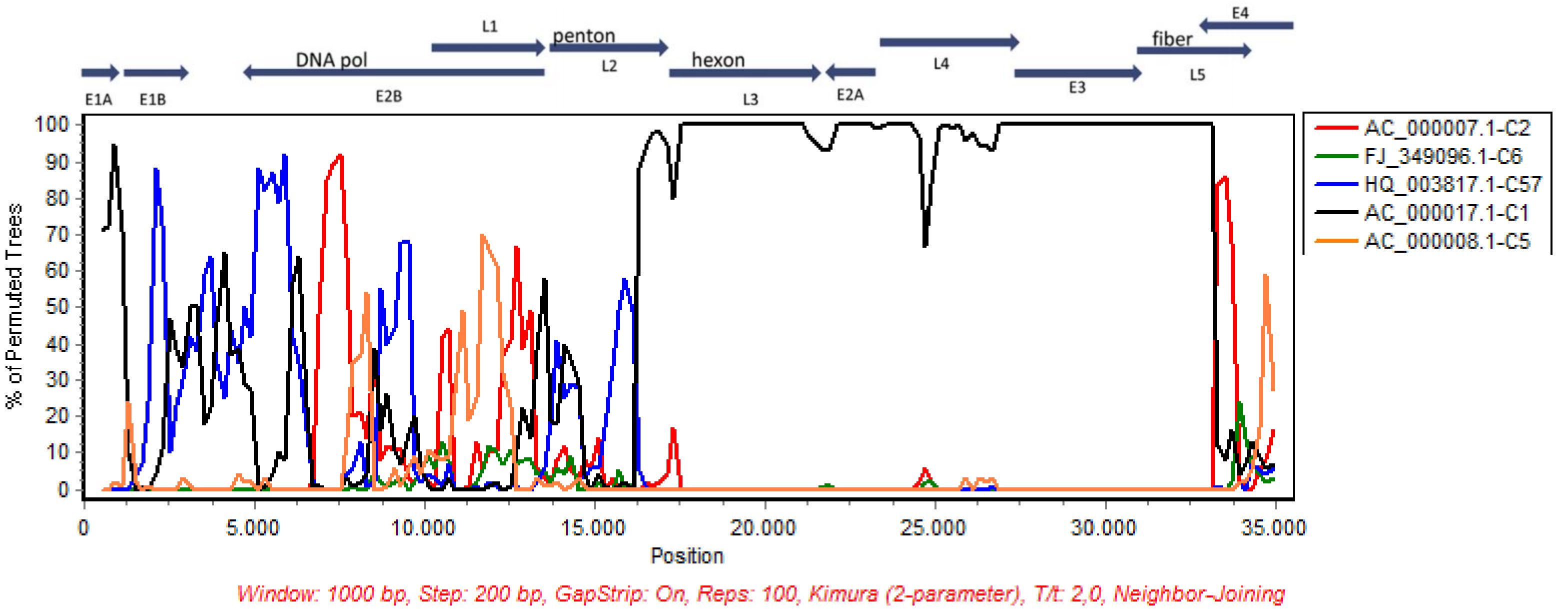

Figure S2

7C1

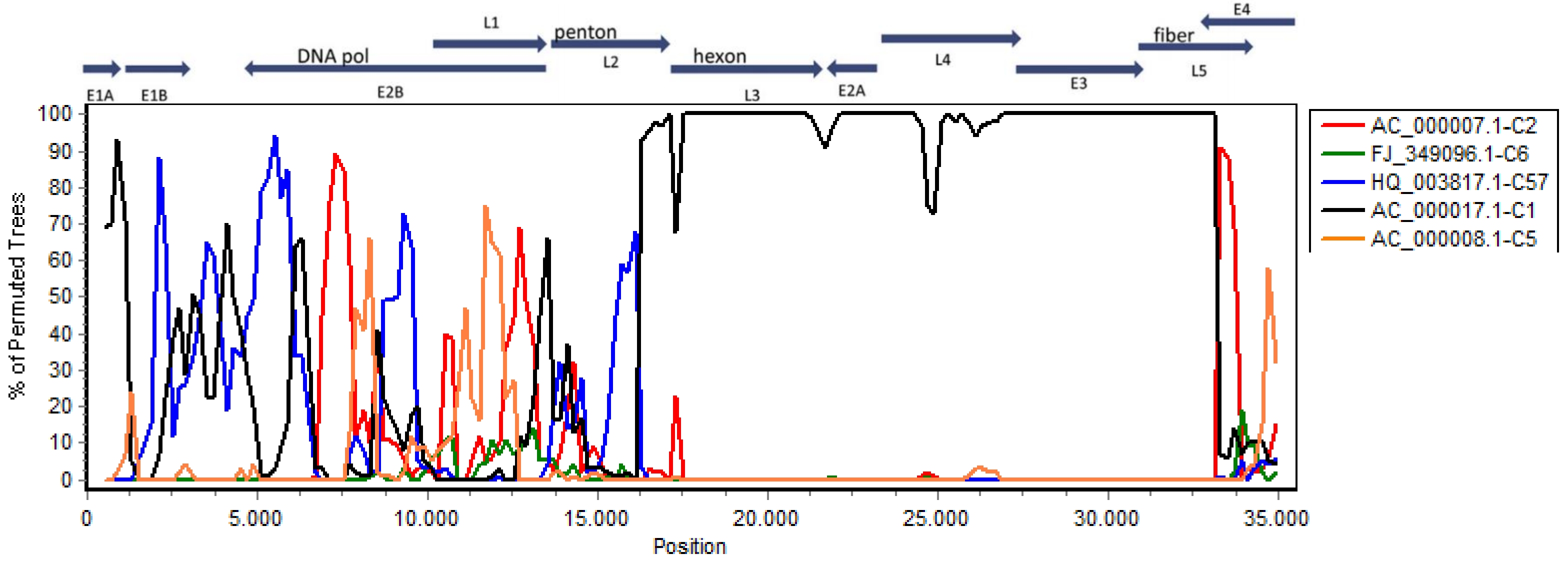

Window: 1000 bp, Step: 200 bp, GapStrip: On, Reps: 100, Kimura (2-parameter), T/t: 2,0, Neighbor-Joining

8C2

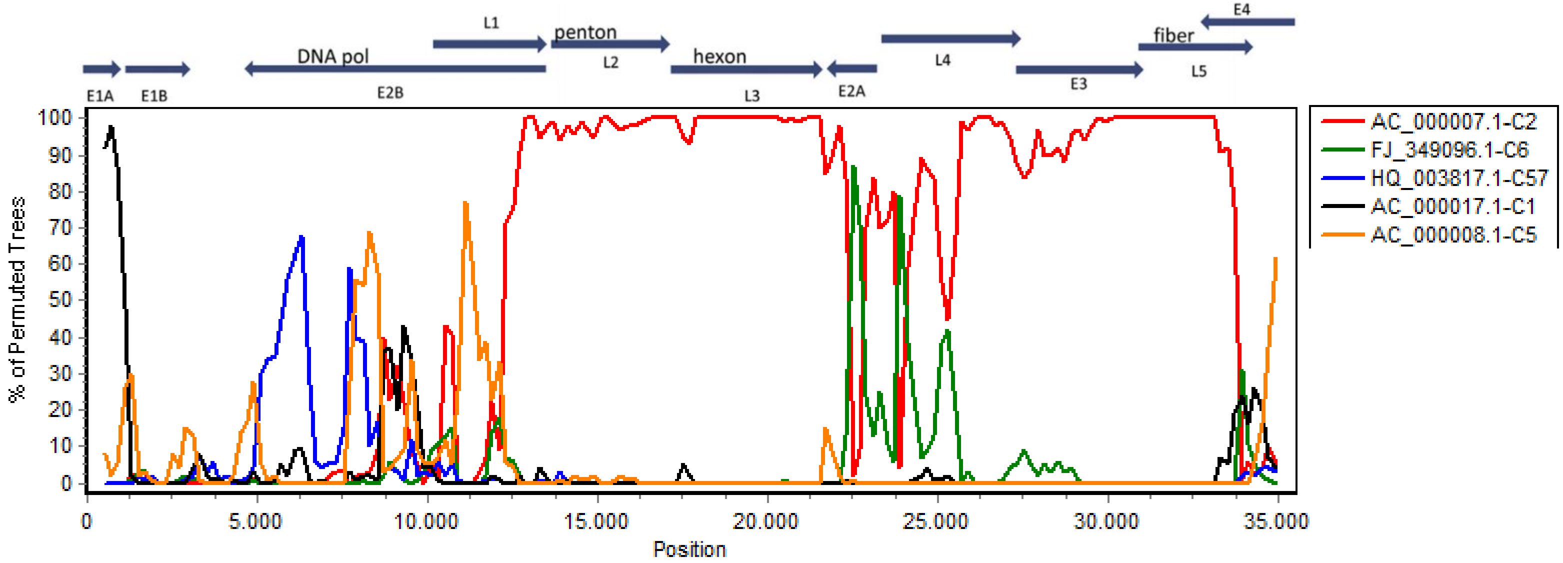

Window: 1000 bp, Step: 200 bp, GapStrip: On, Reps: 100, Kimura (2-parameter), T/t: 2,0, Neighbor-Joining

Figure S2

9C2

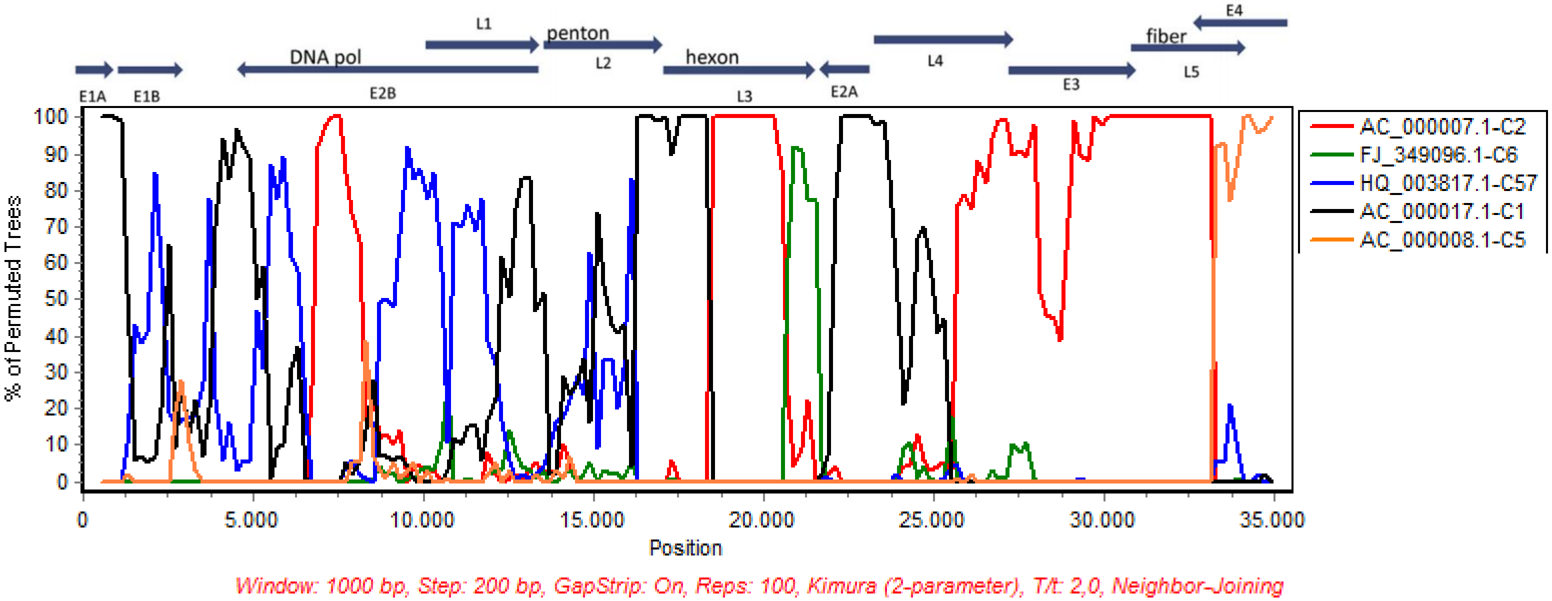

10C2

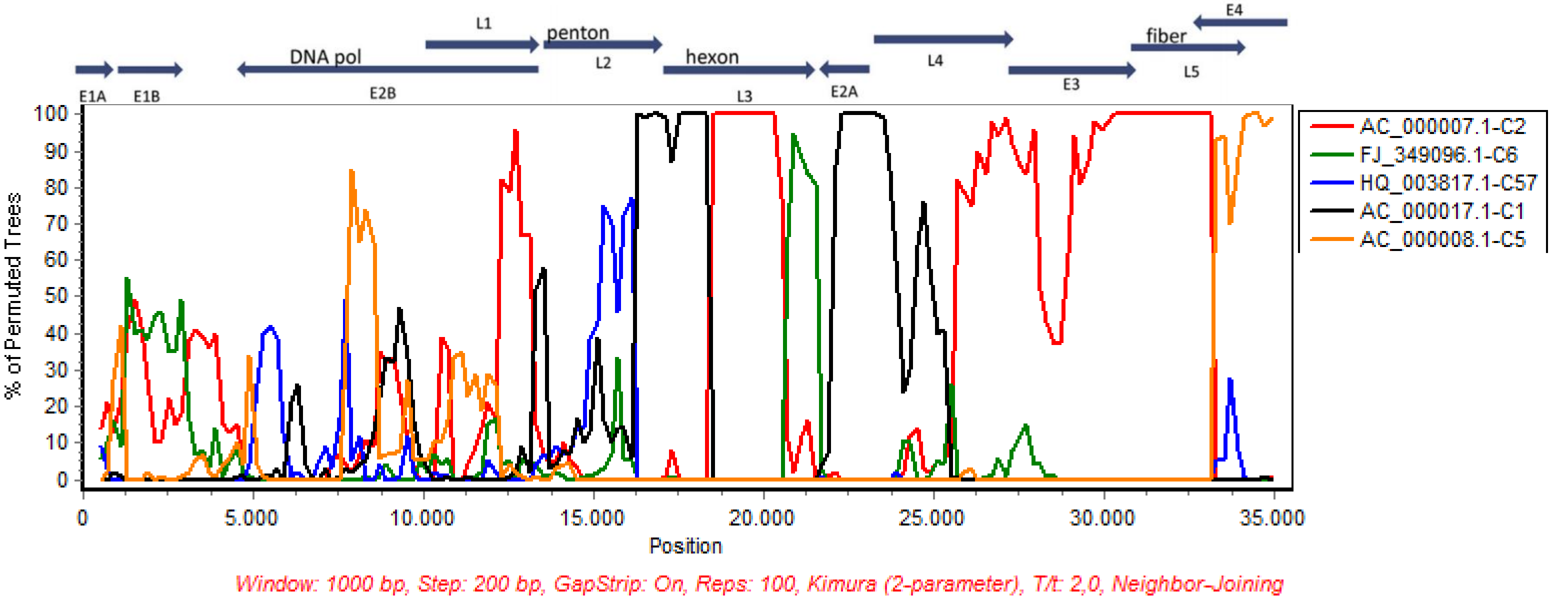

Figure S2

11C2

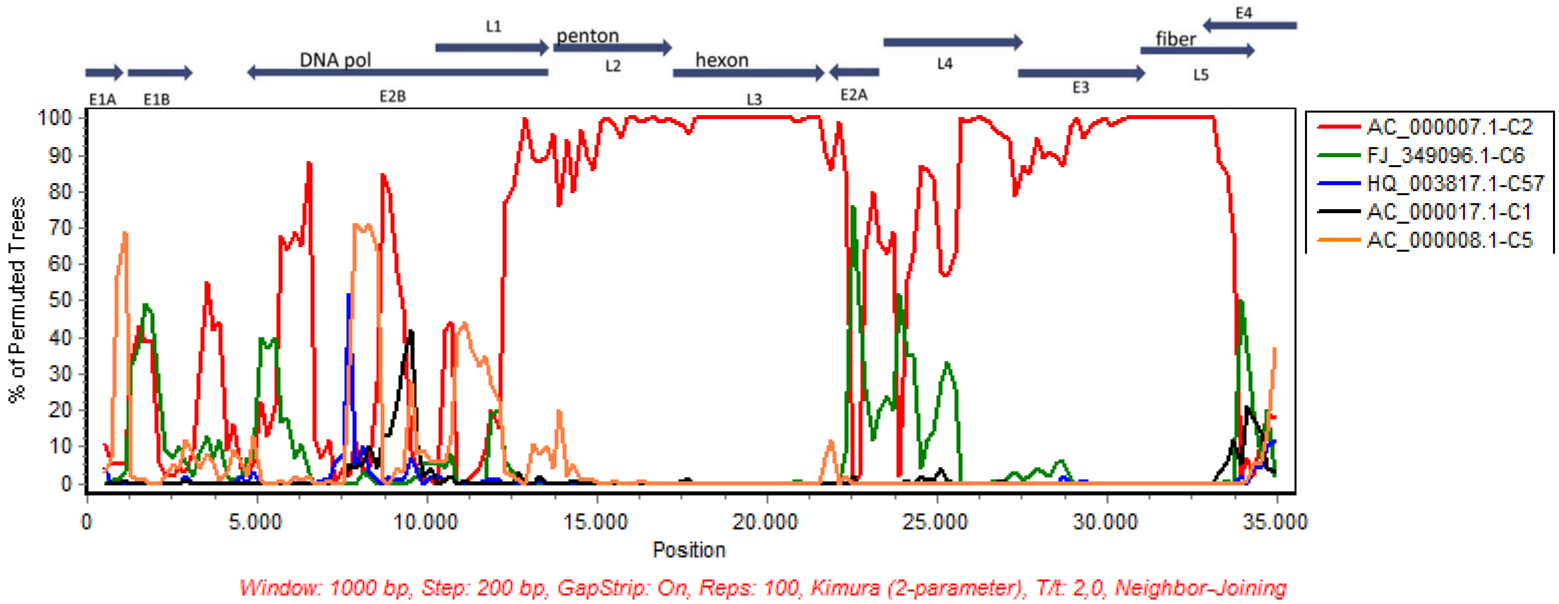

12C1

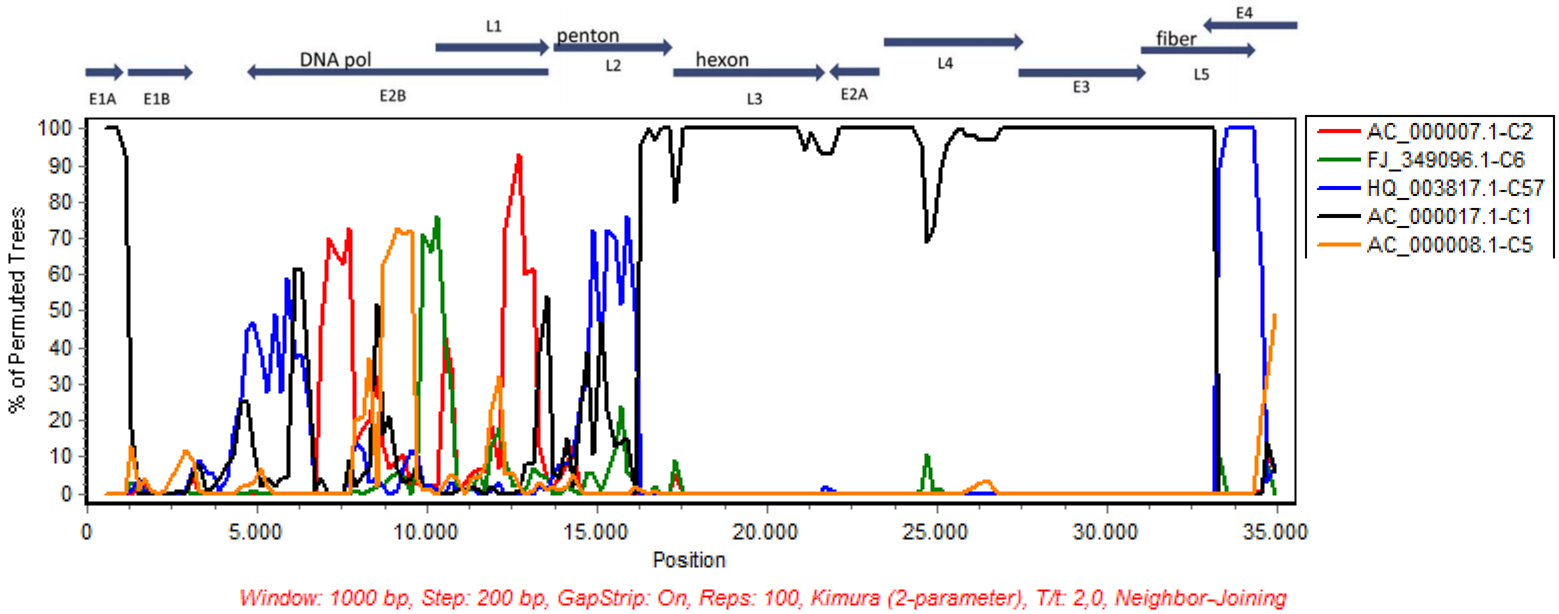

Figure S2

13C1

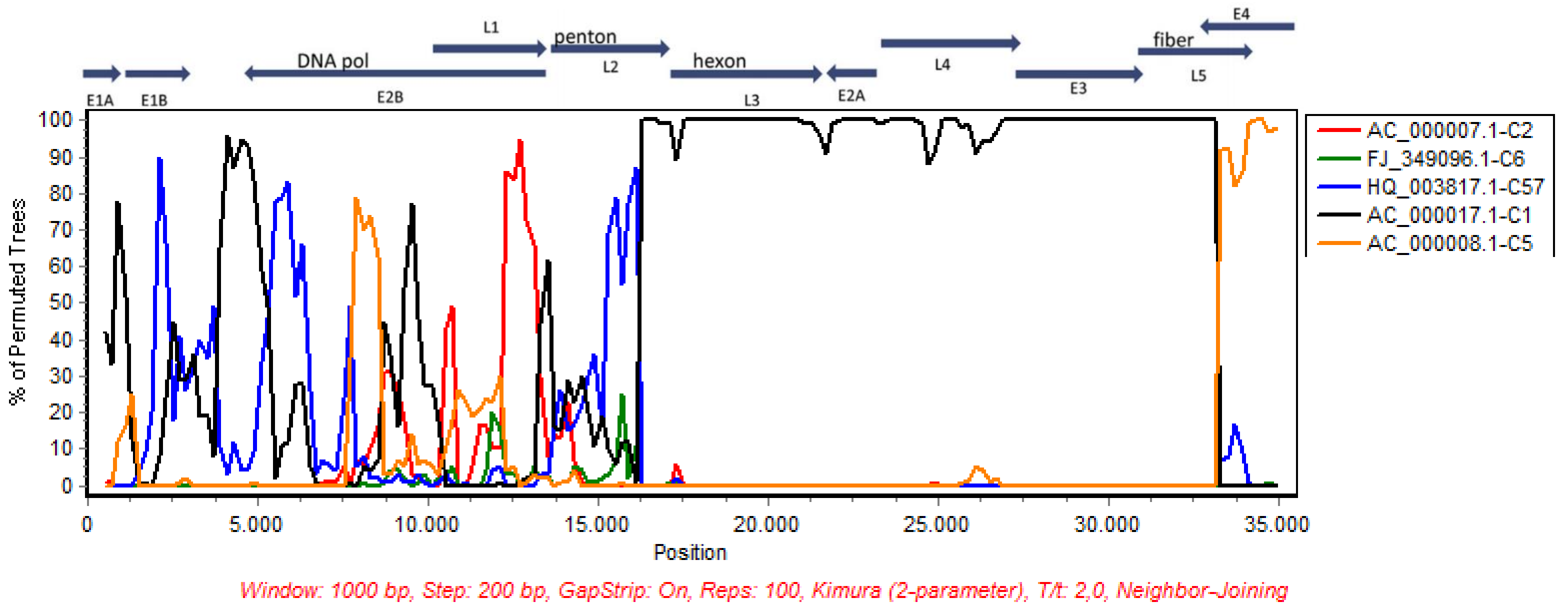

14C2

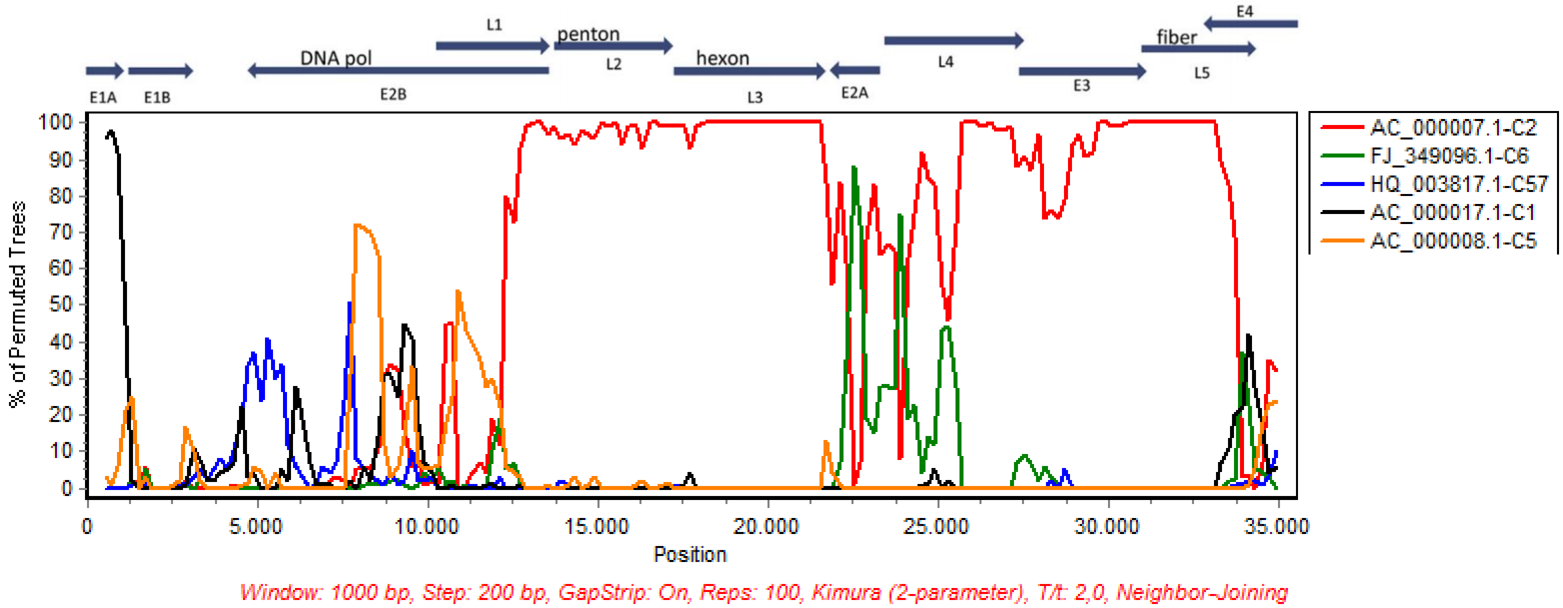

Figure S2

15C2

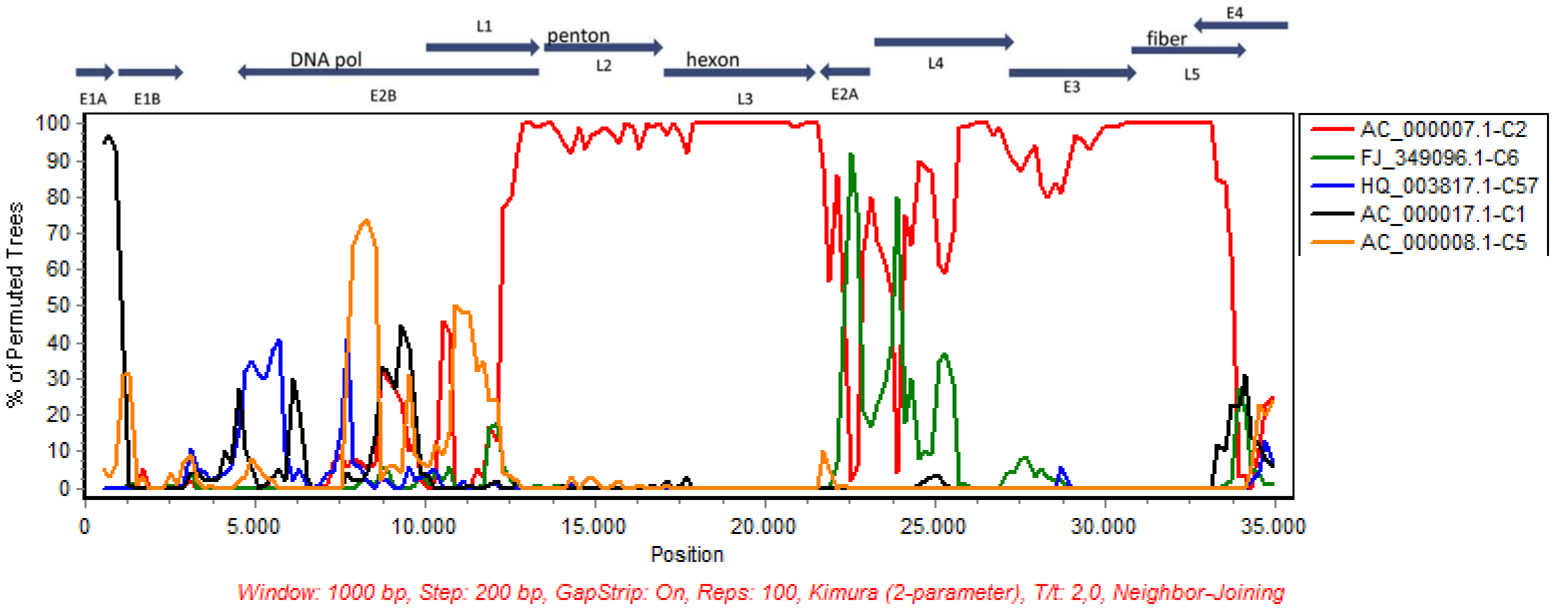

16C2

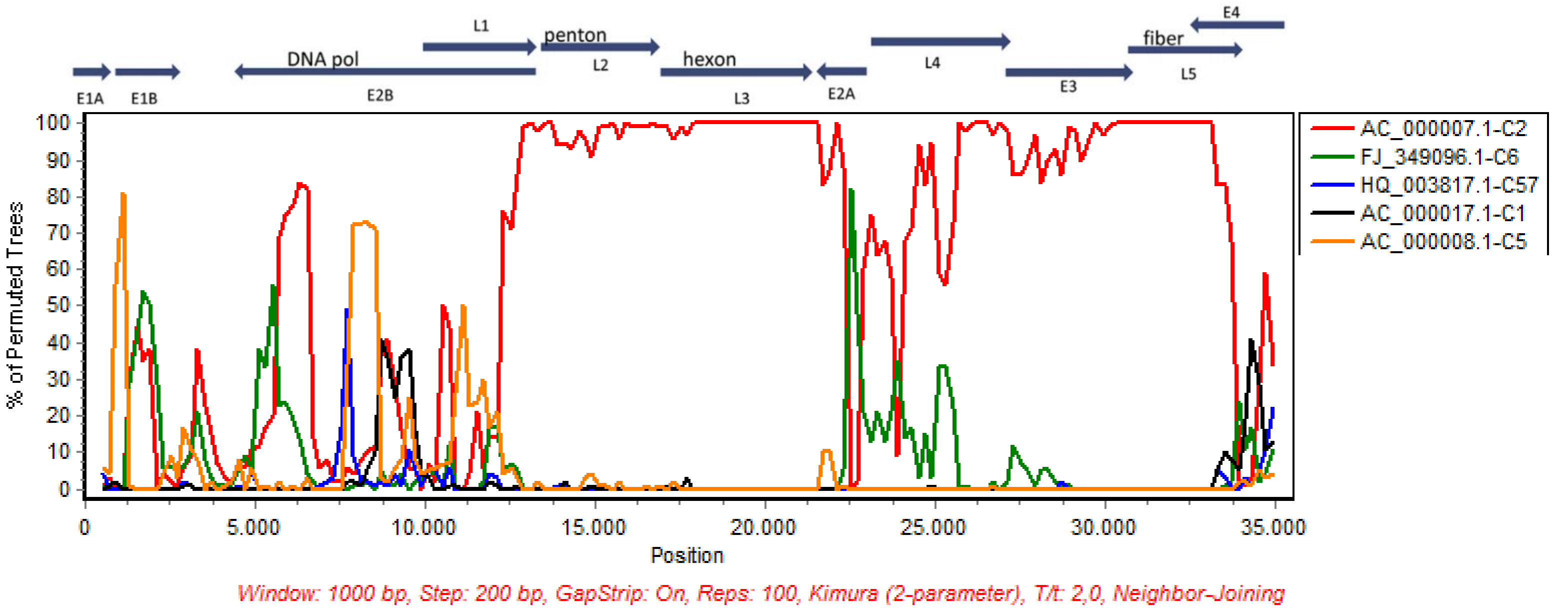

Figure S2

17C2

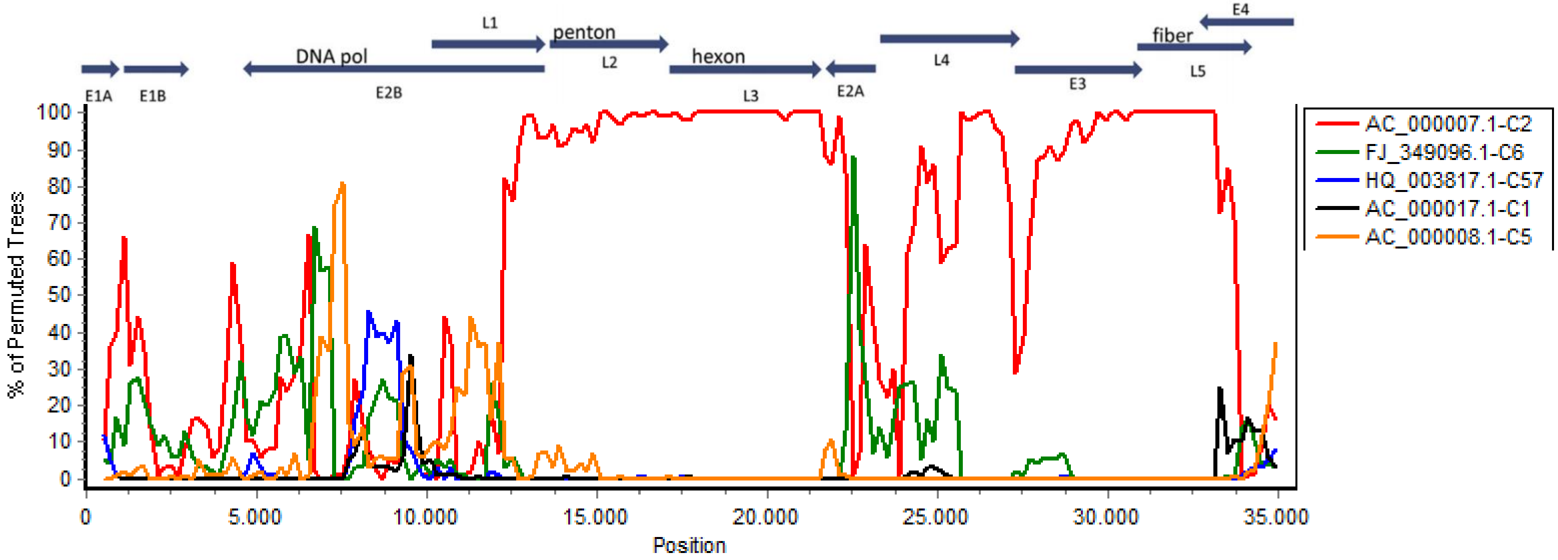

Window: 1000 bp, Step: 200 bp, GapStrip: On, Reps: 100, Kimura (2-parameter), T/t: 2,0, Neighbor-Joining

18C1

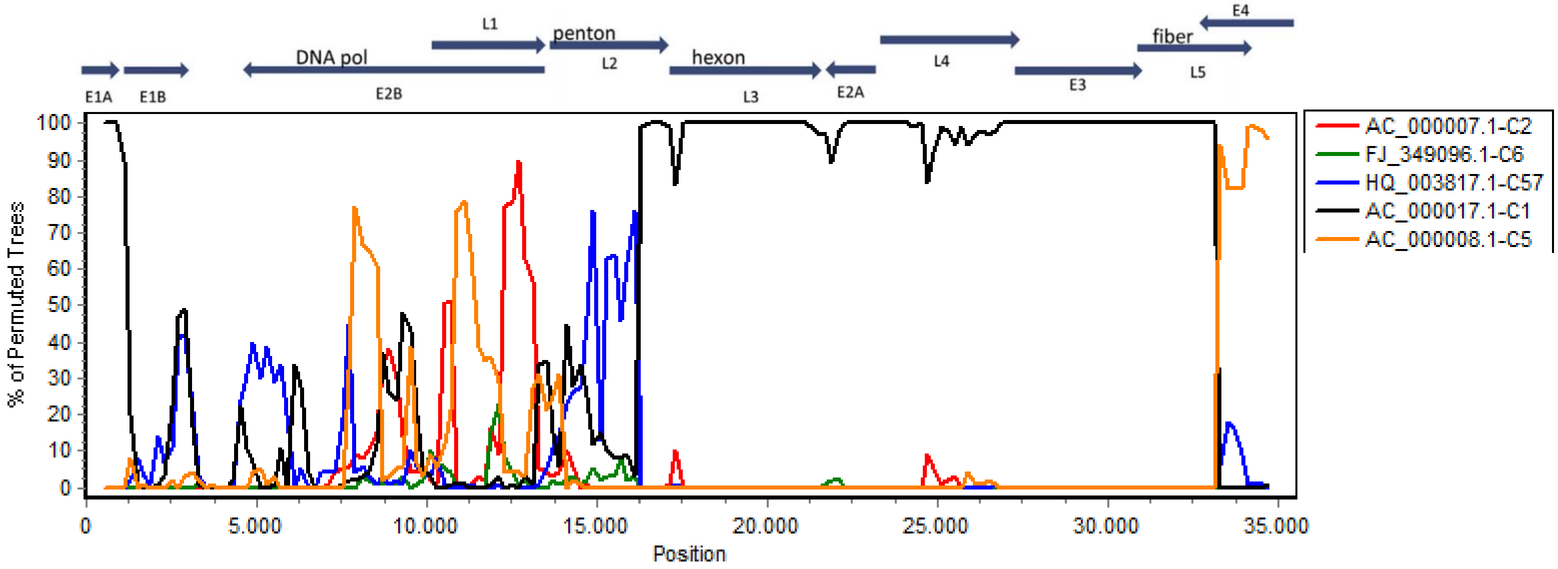

Window: 1000 bp, Step: 200 bp, GapStrip: On, Reps: 100, Kimura (2-parameter), T/t: 2,0, Neighbor-Joining

Figure S2

19C2

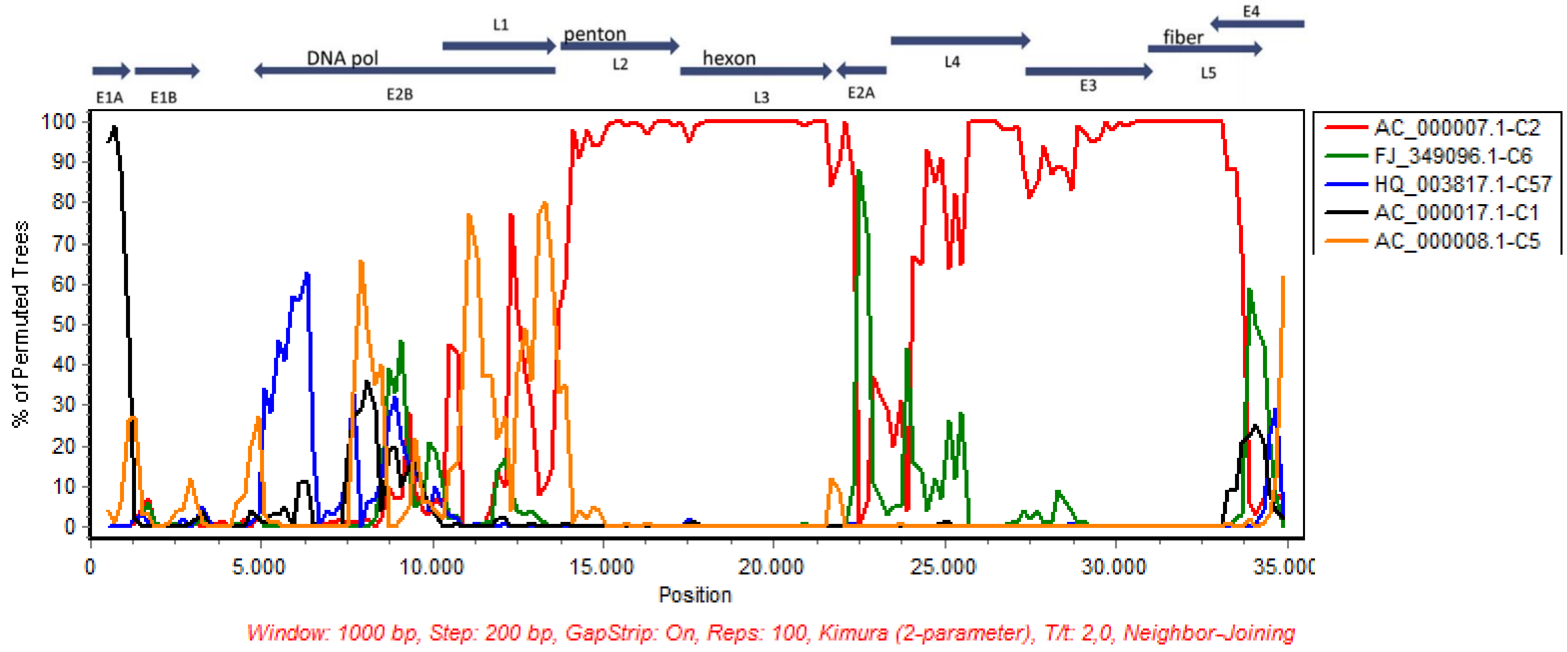

20C1

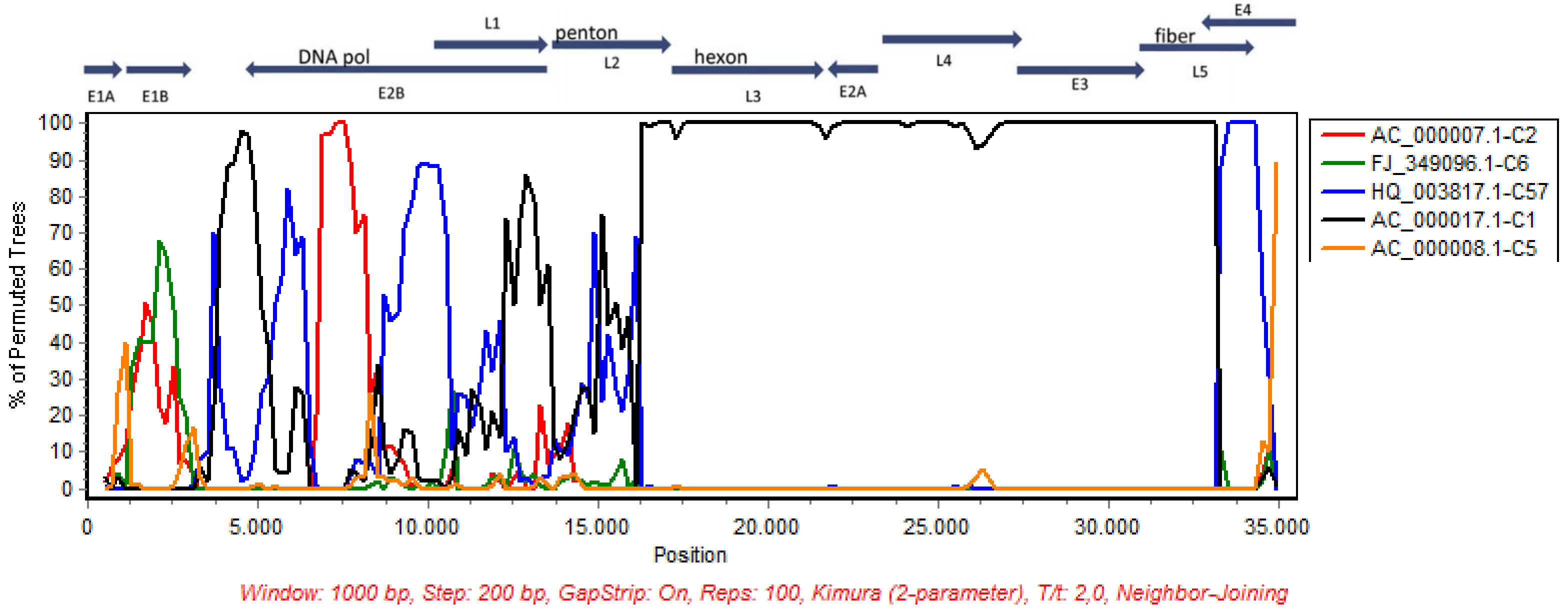

Figure S2

21C2

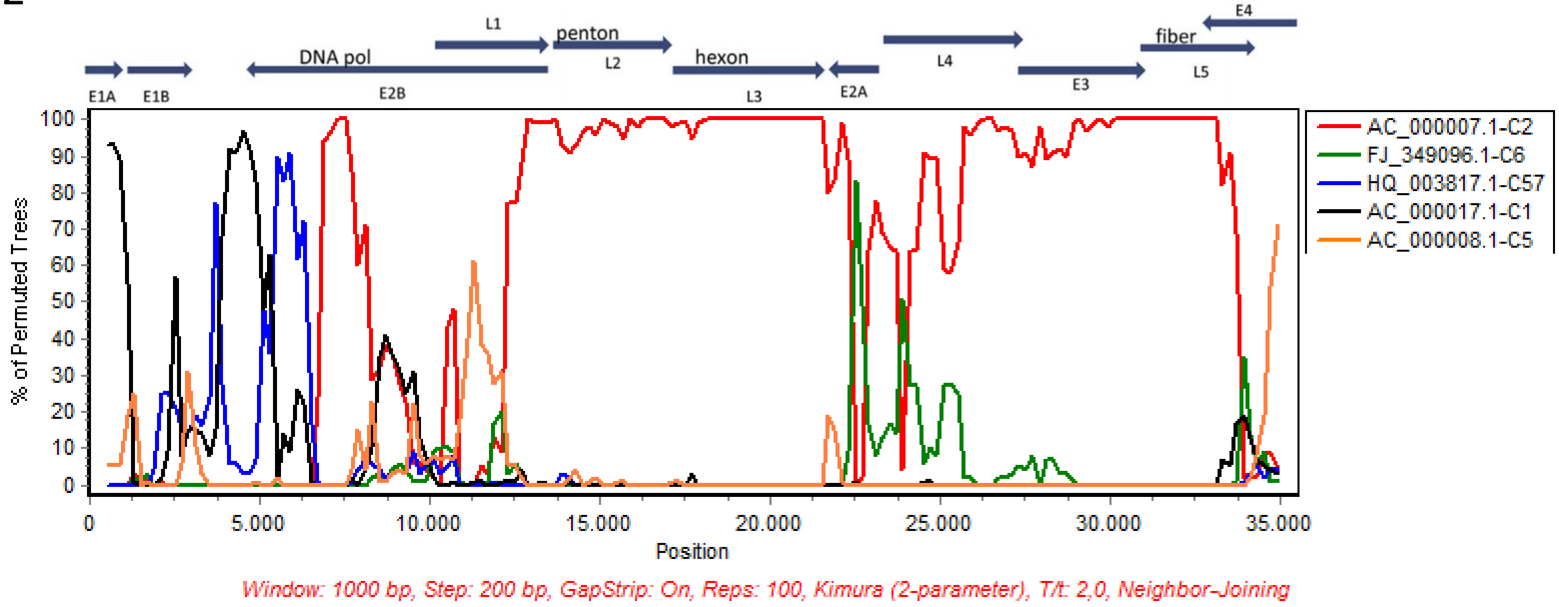

22C1

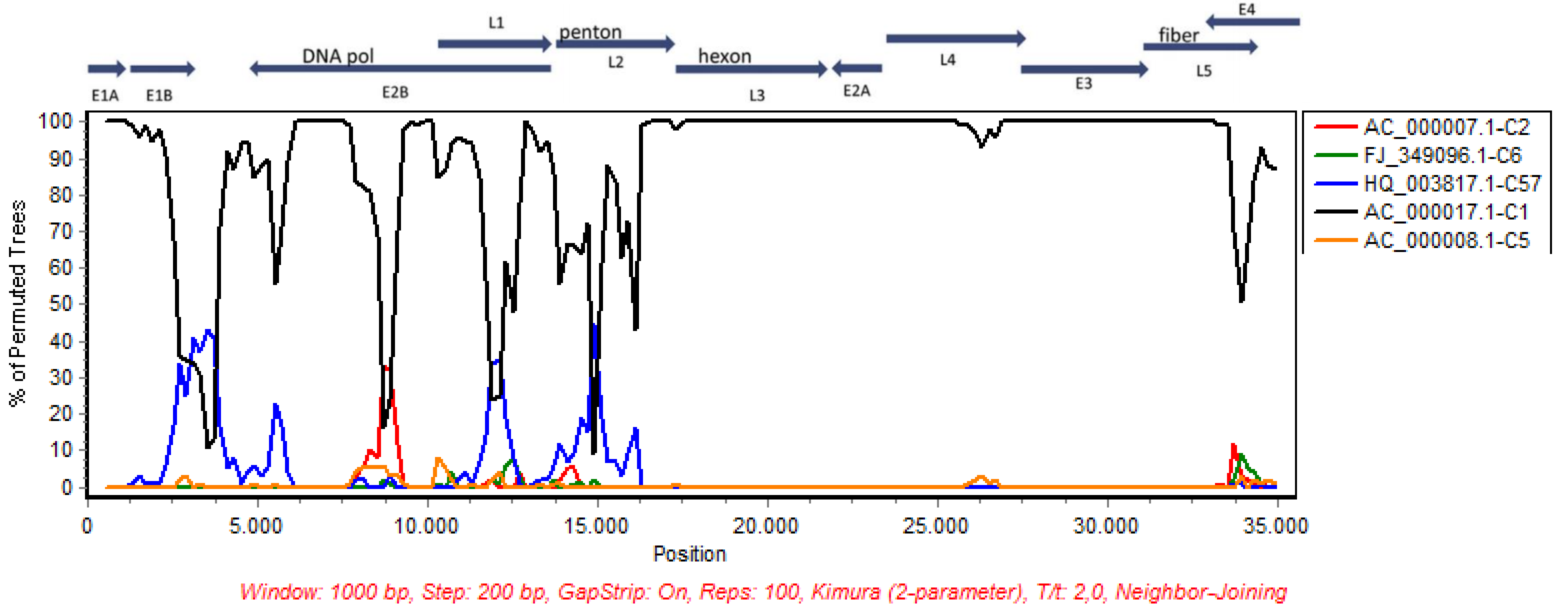

Figure S2

23C2

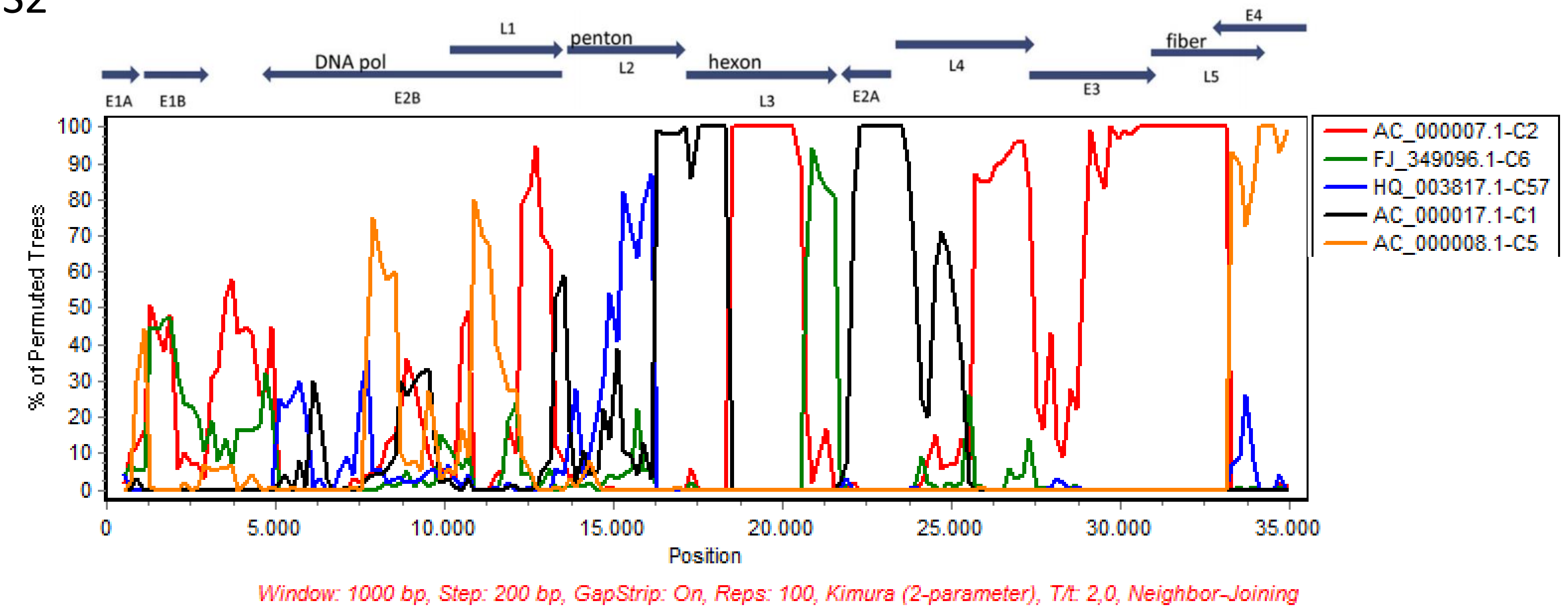

24C2

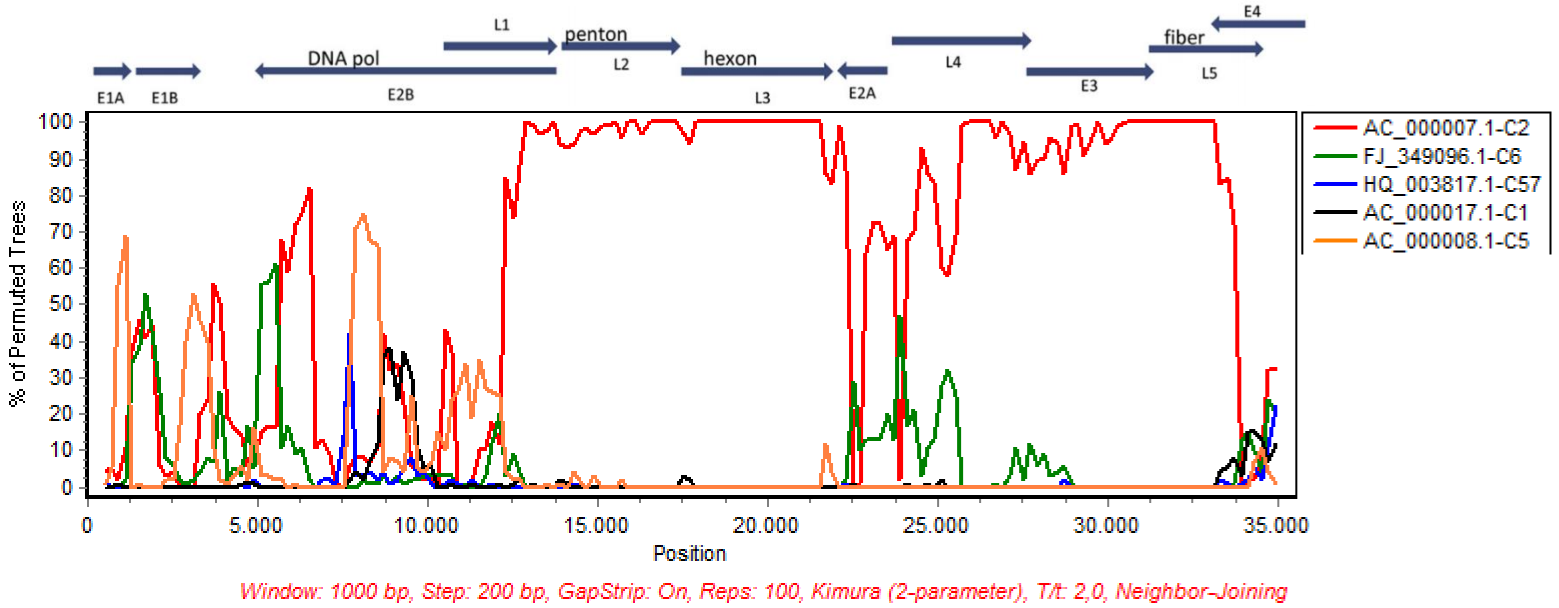

Figure S2

25C5

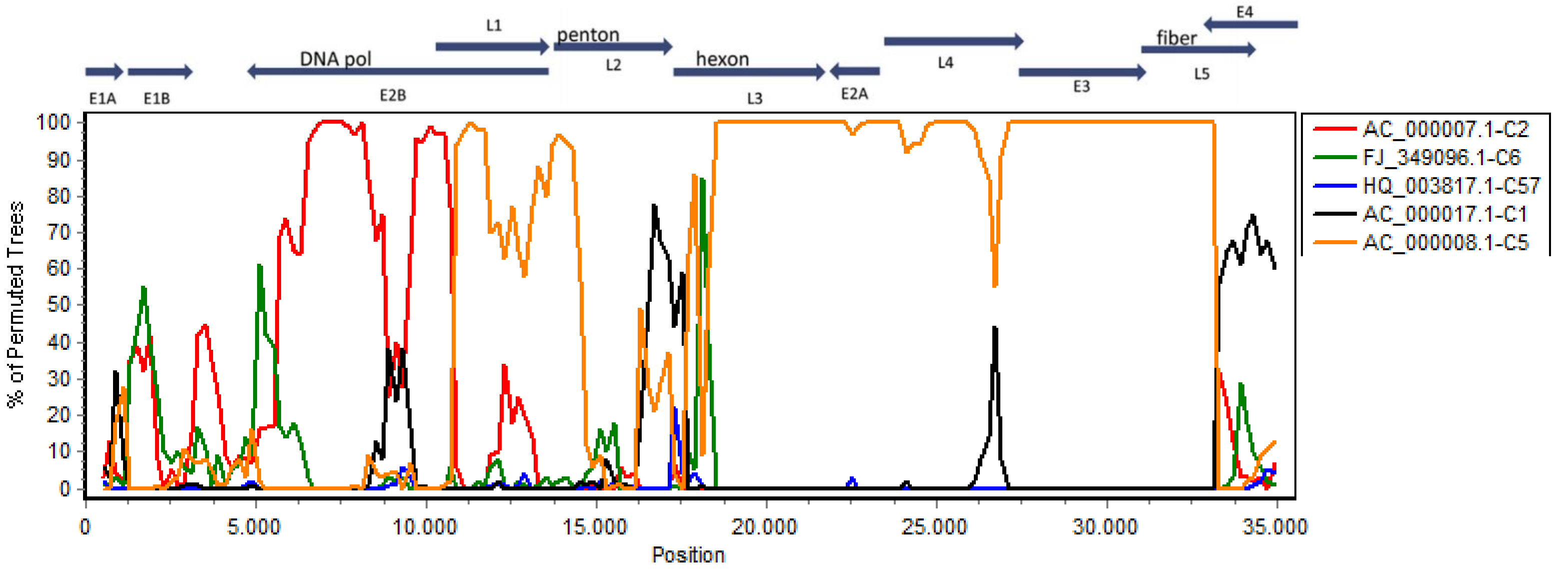

Window: 1000 bp, Step: 200 bp, GapStrip: On, Reps: 100, Kimura (2-parameter), T/t: 2,0, Neighbor-Joining

26C2

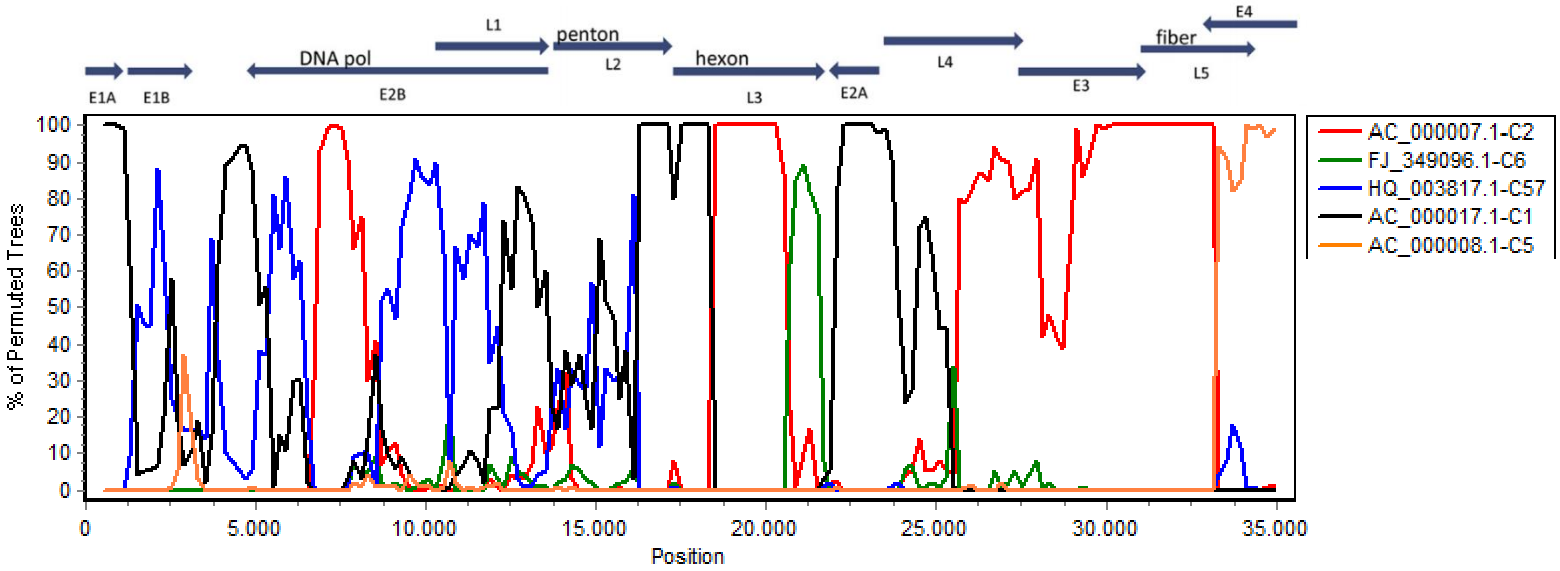

Window: 1000 bp, Step: 200 bp, GapStrip: On, Reps: 100, Kimura (2-parameter), T/t: 2,0, Neighbor-Joining

Figure S2

27C2

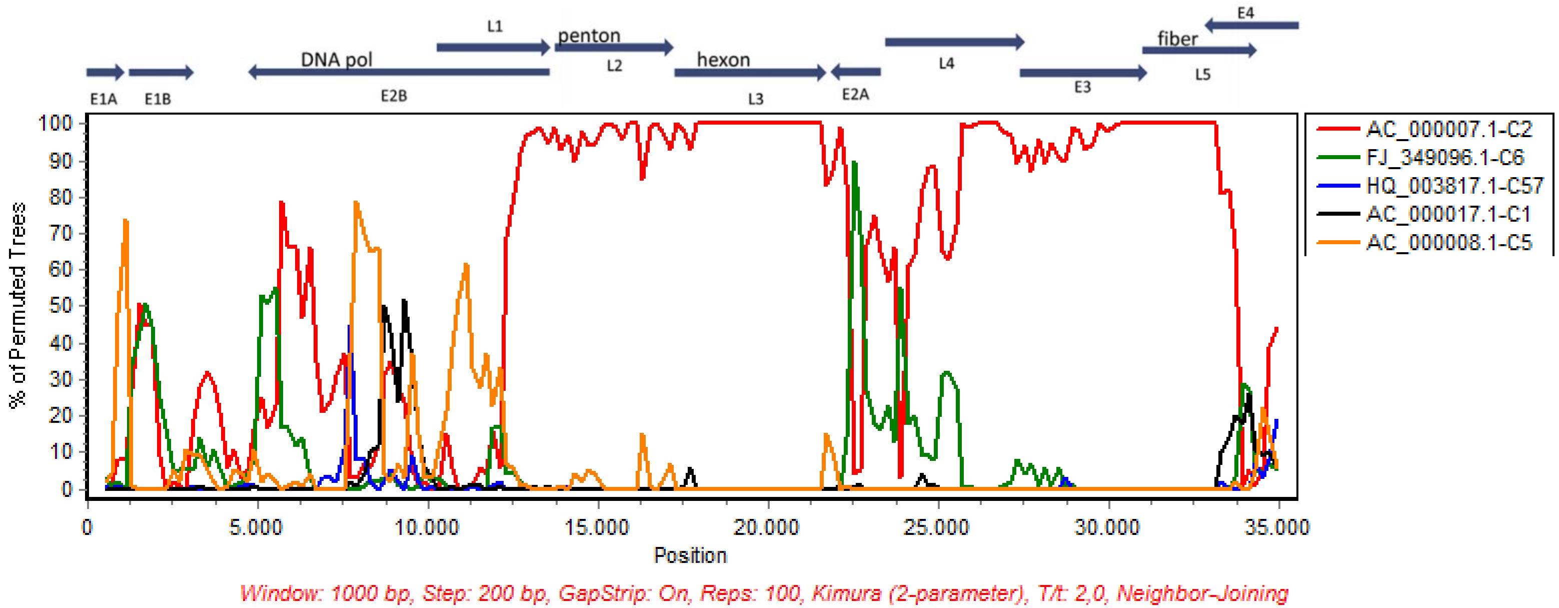

28C5

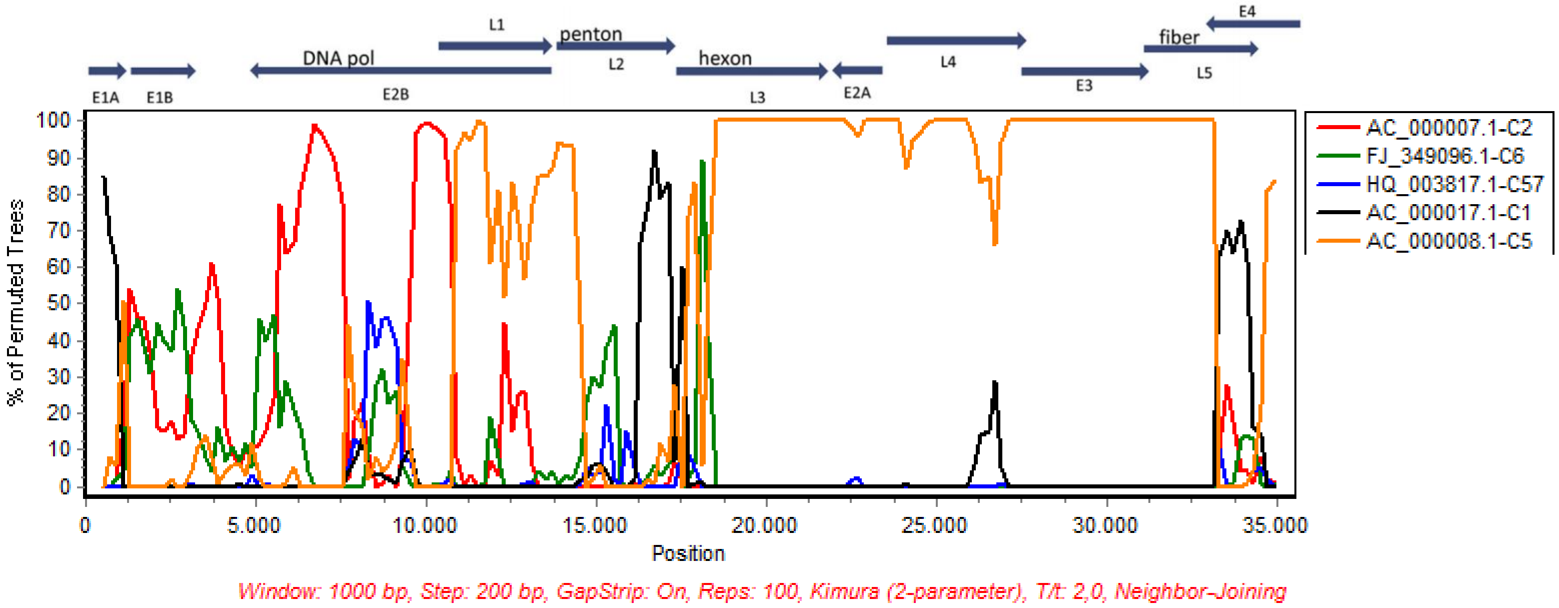

Figure S2

29C2

30C1

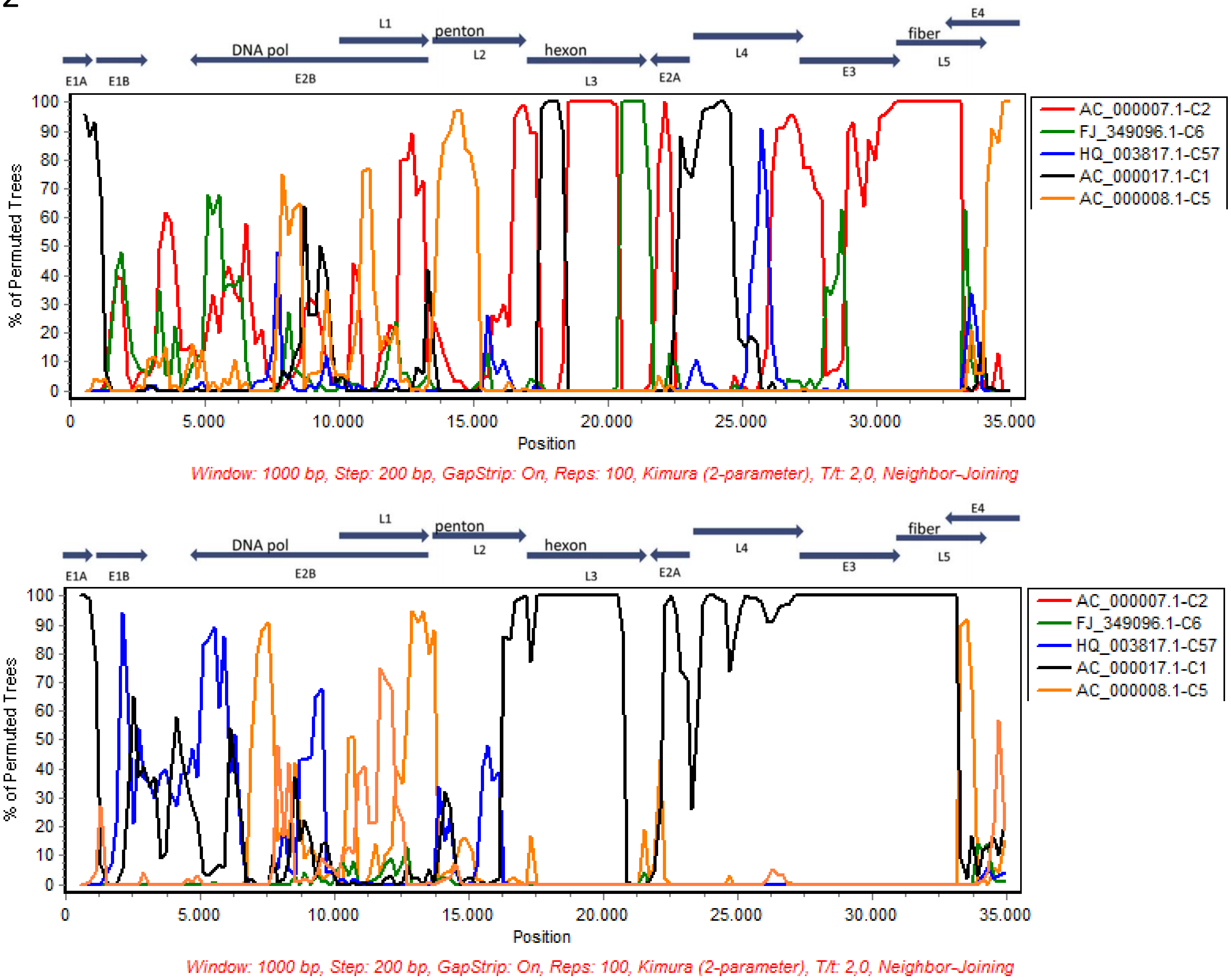

Figure S2

31C2

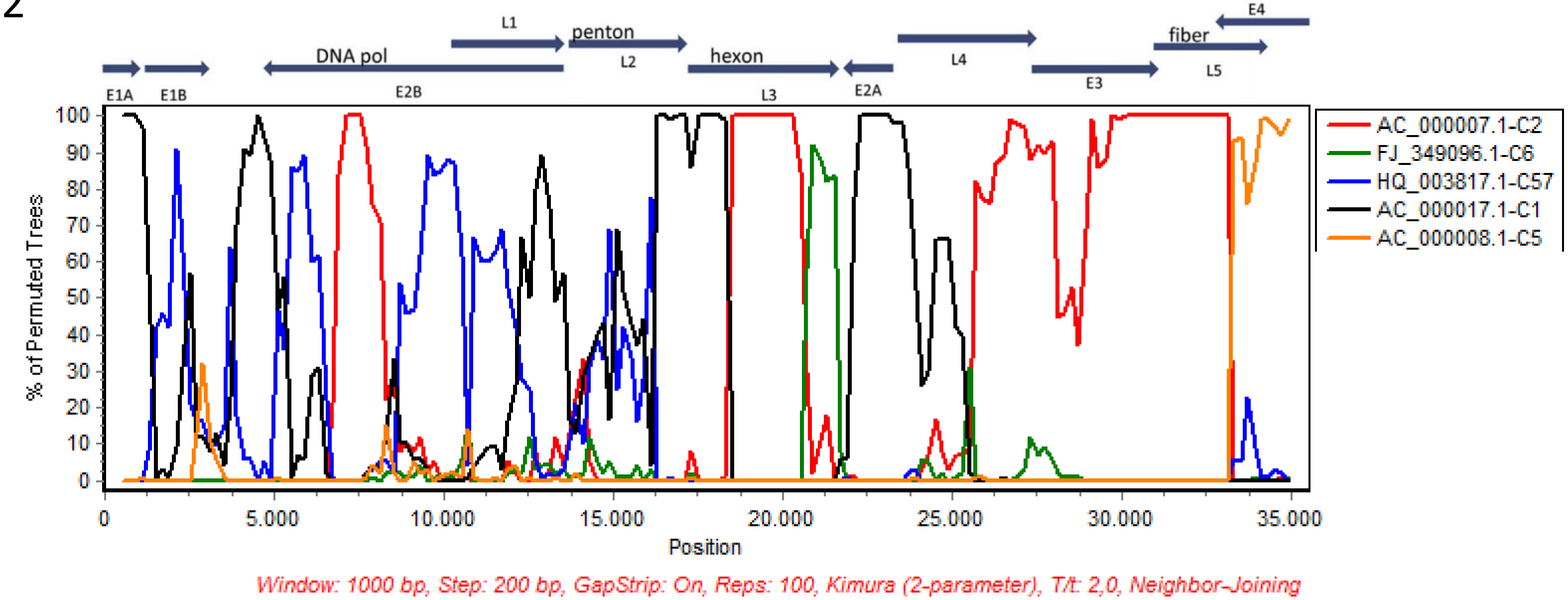

32C1

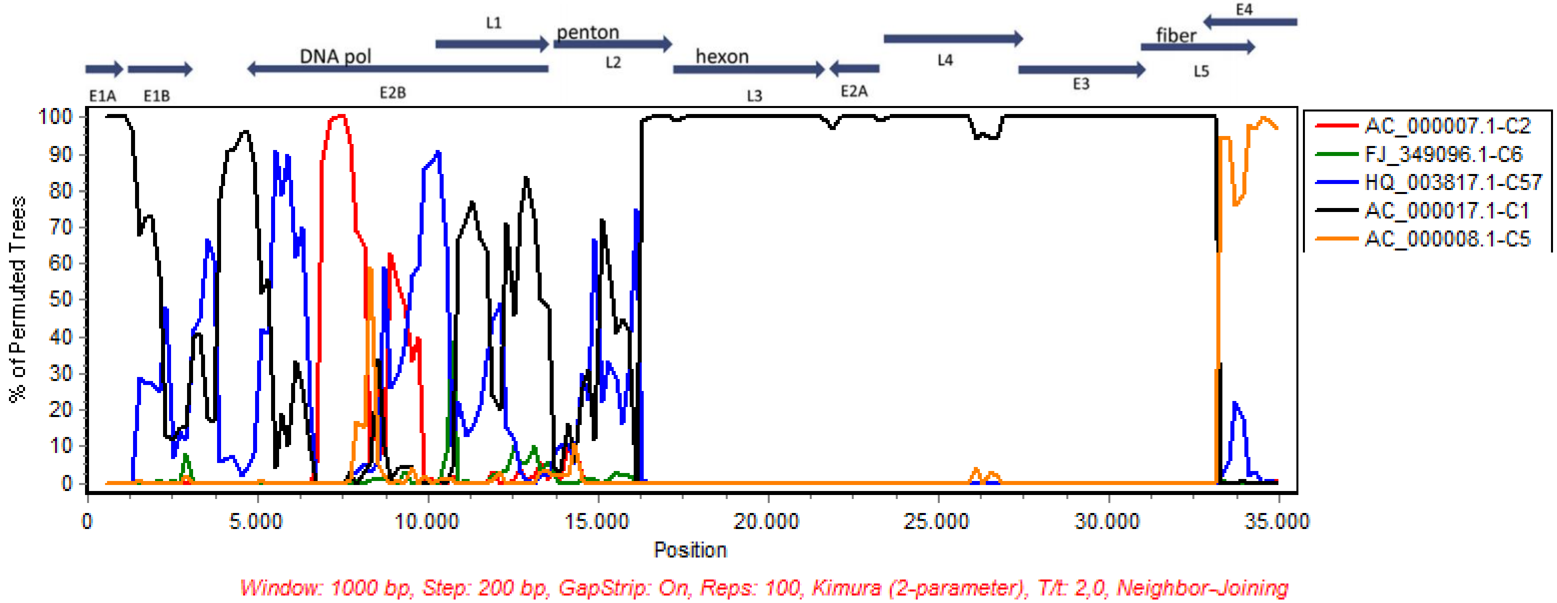

Figure S2

33C2

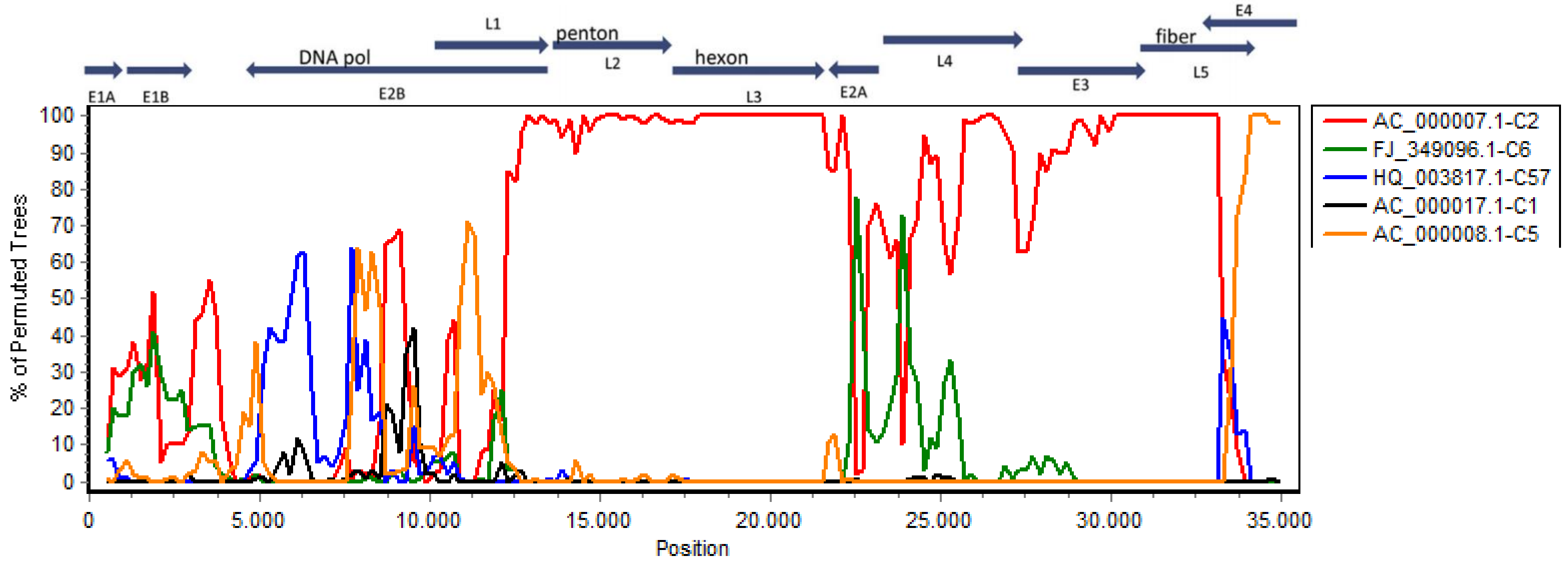

Window: 1000 bp, Step: 200 bp, GapStrip: On, Reps: 100, Kimura (2-parameter), T/t: 2,0, Neighbor-Joining

34C2

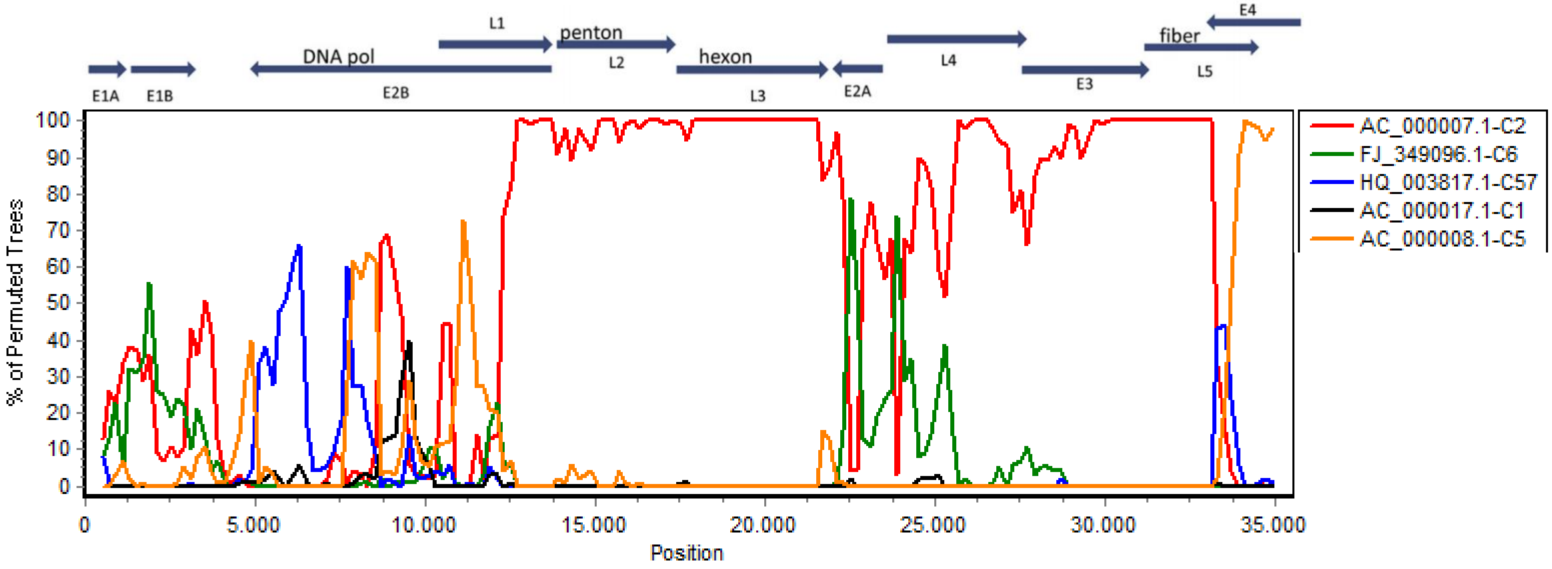

Window: 1000 bp, Step: 200 bp, GapStrip: On, Reps: 100, Kimura (2-parameter), T/t: 2,0, Neighbor-Joining

Figure S2

35C2

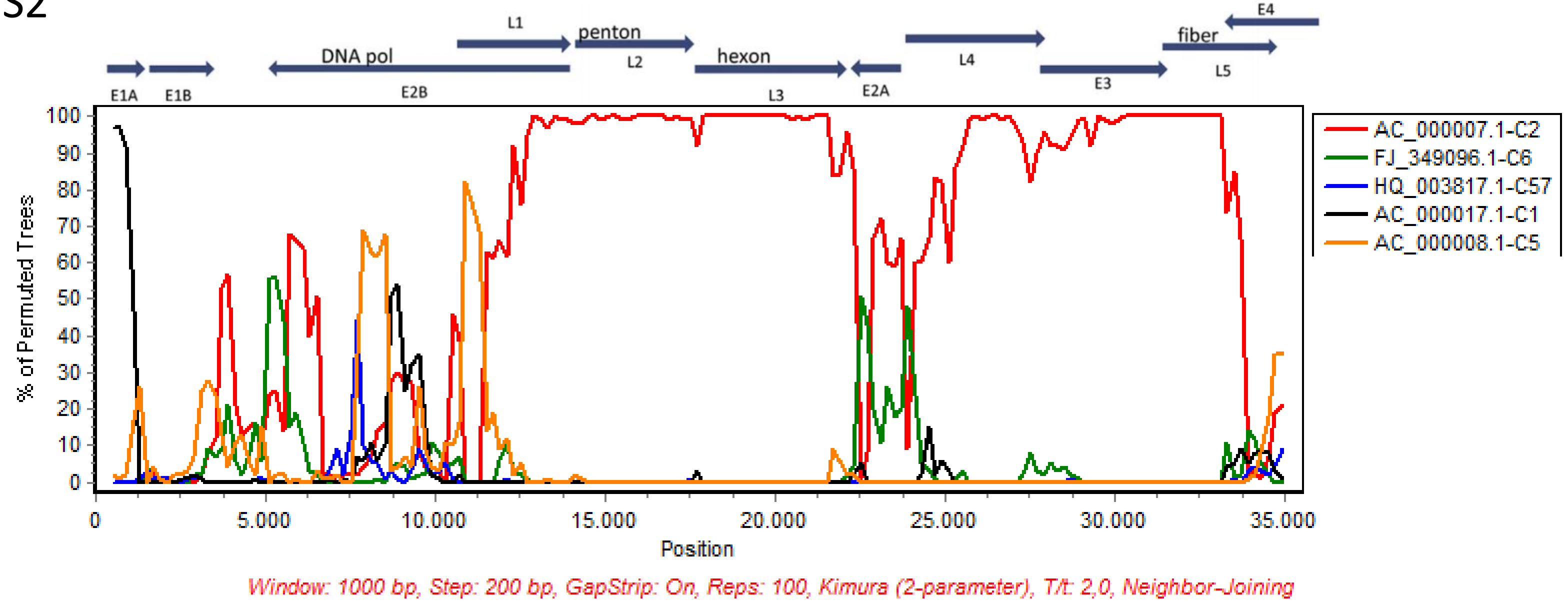

36C2

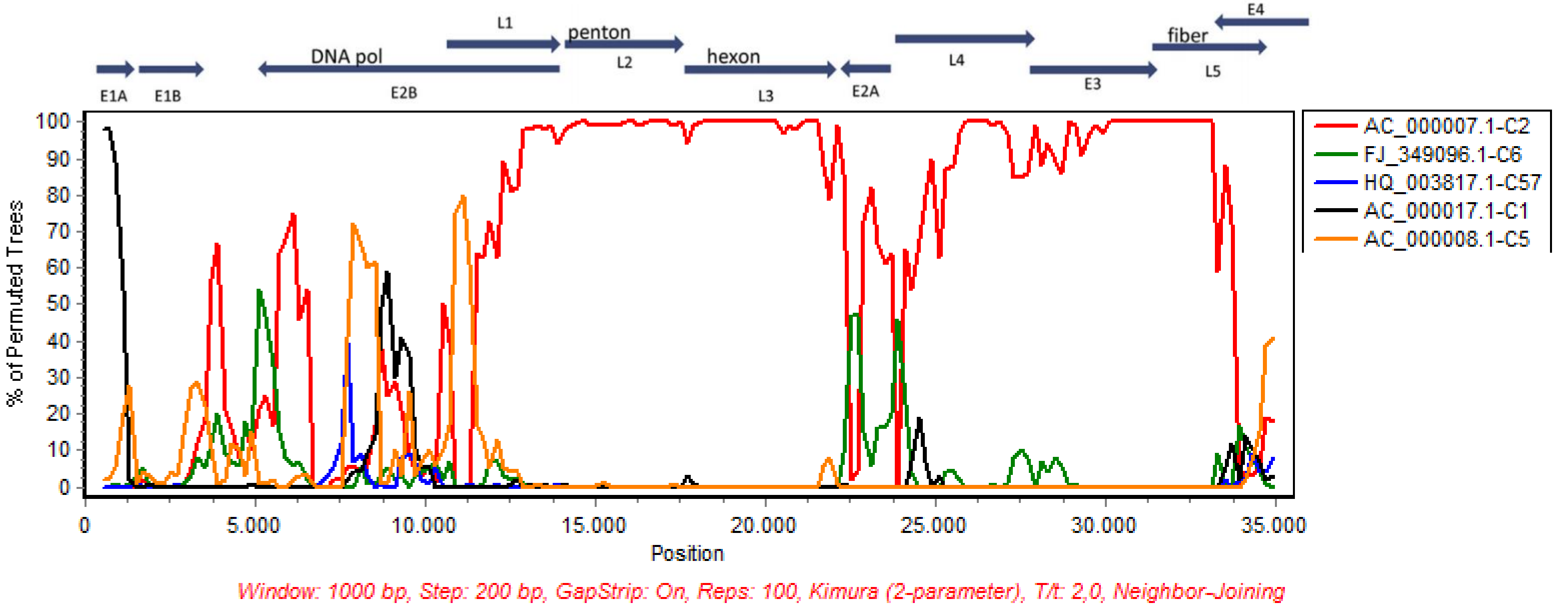

Figure S2

37C2

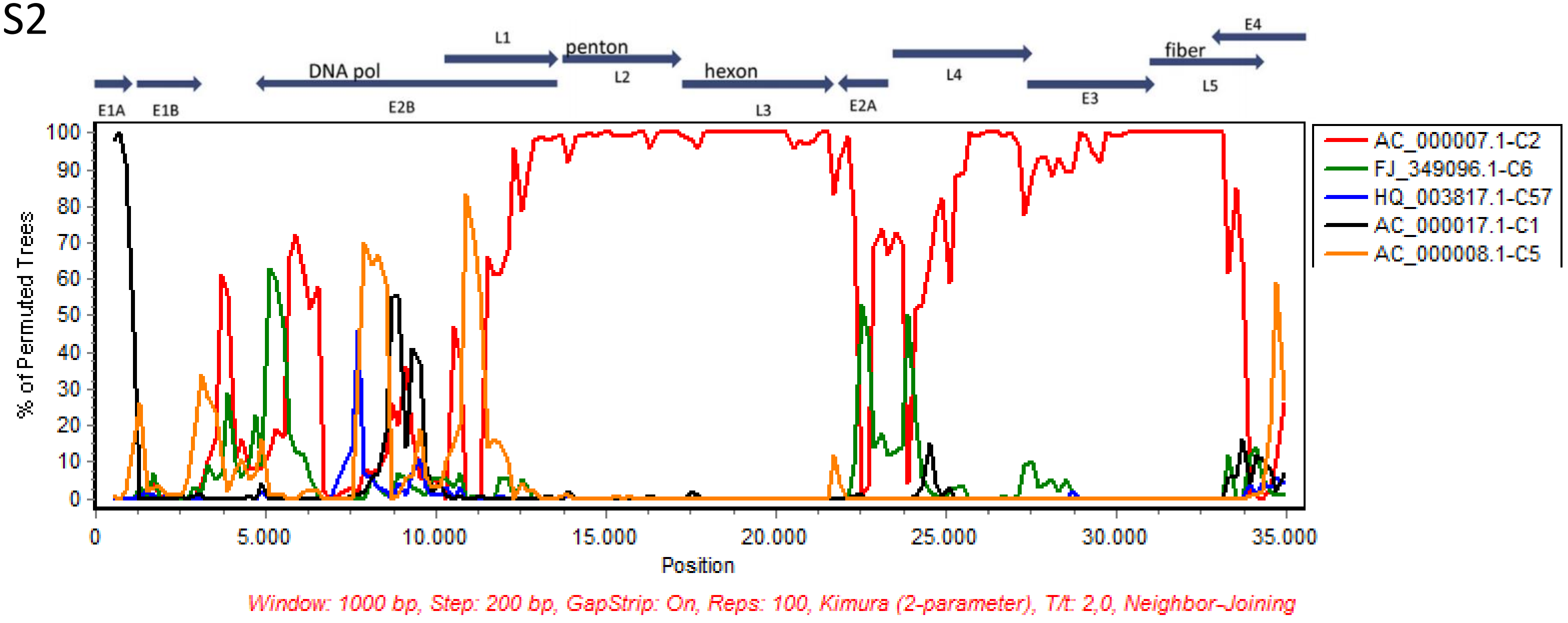

38C2

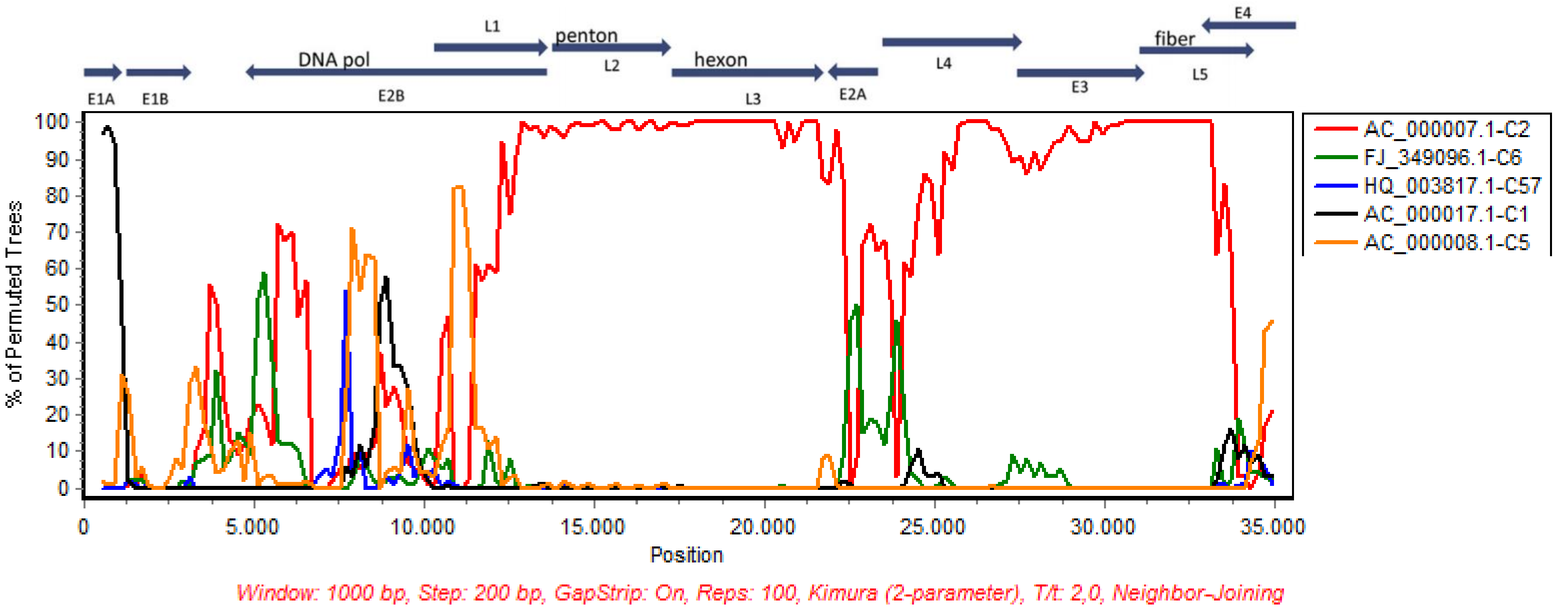

Figure S2

39C2

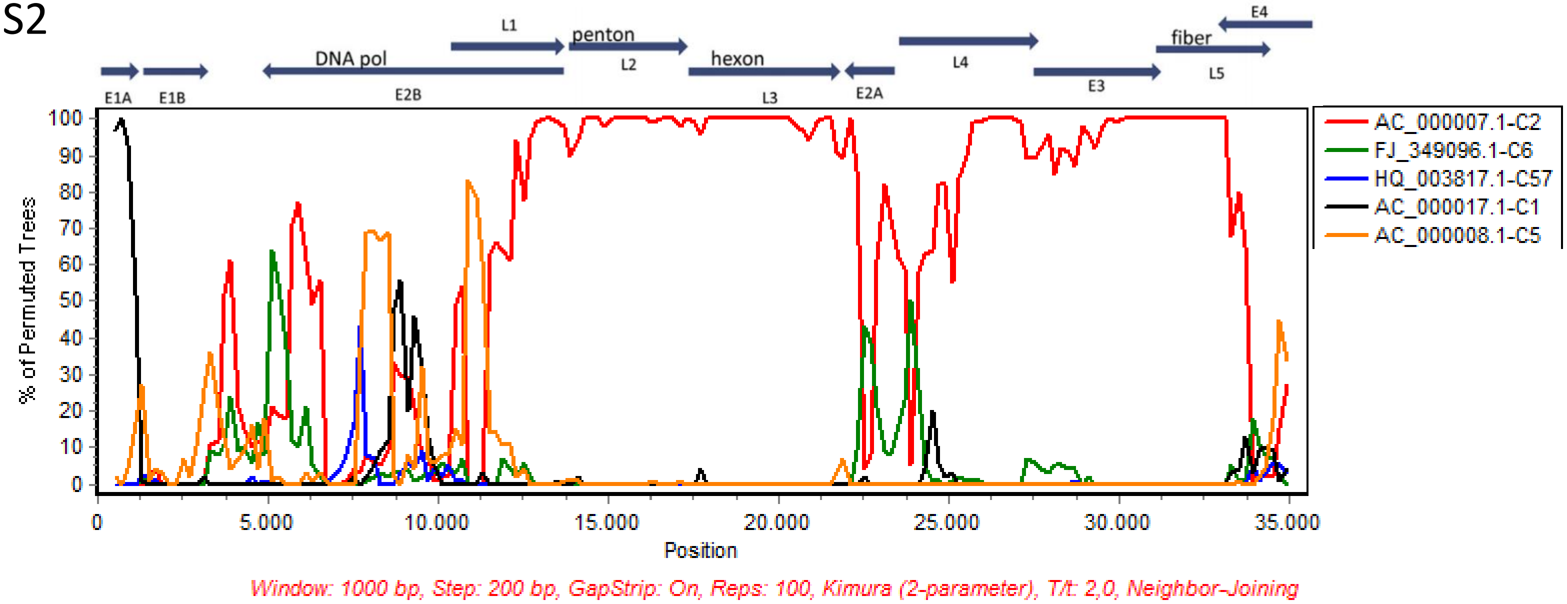

40C5

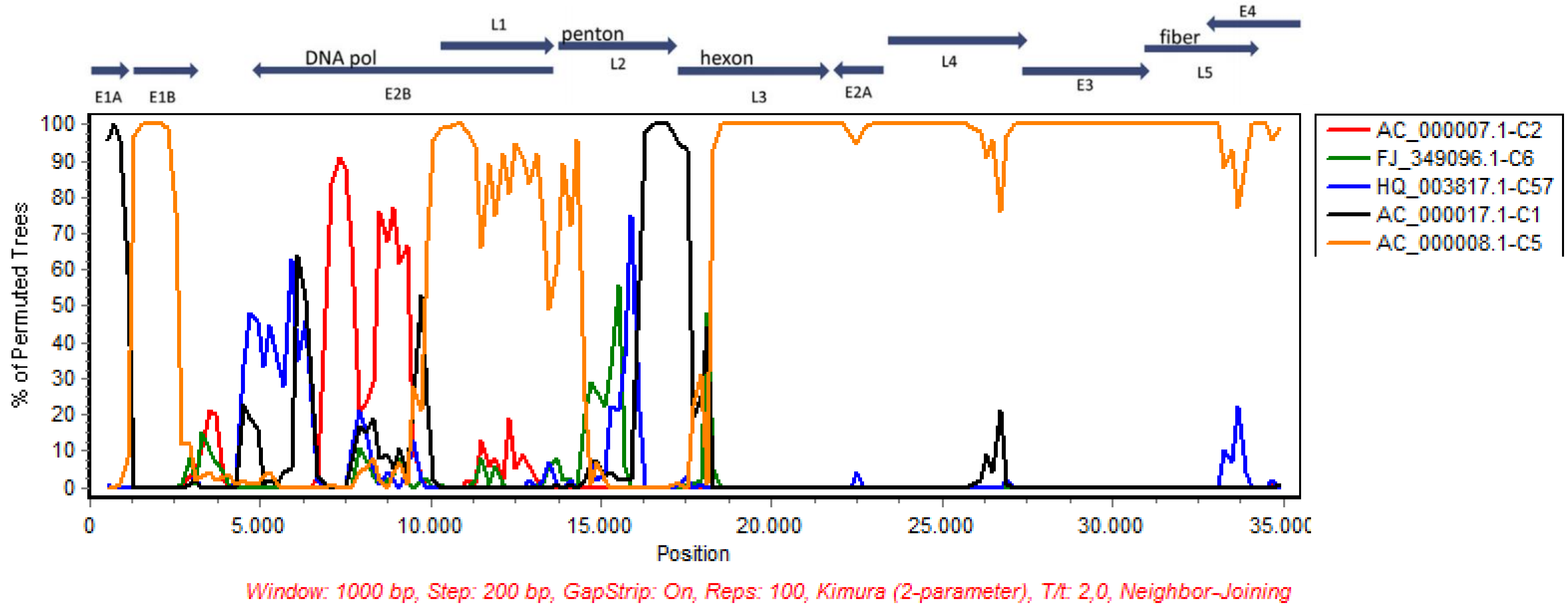

Figure S2

41C1

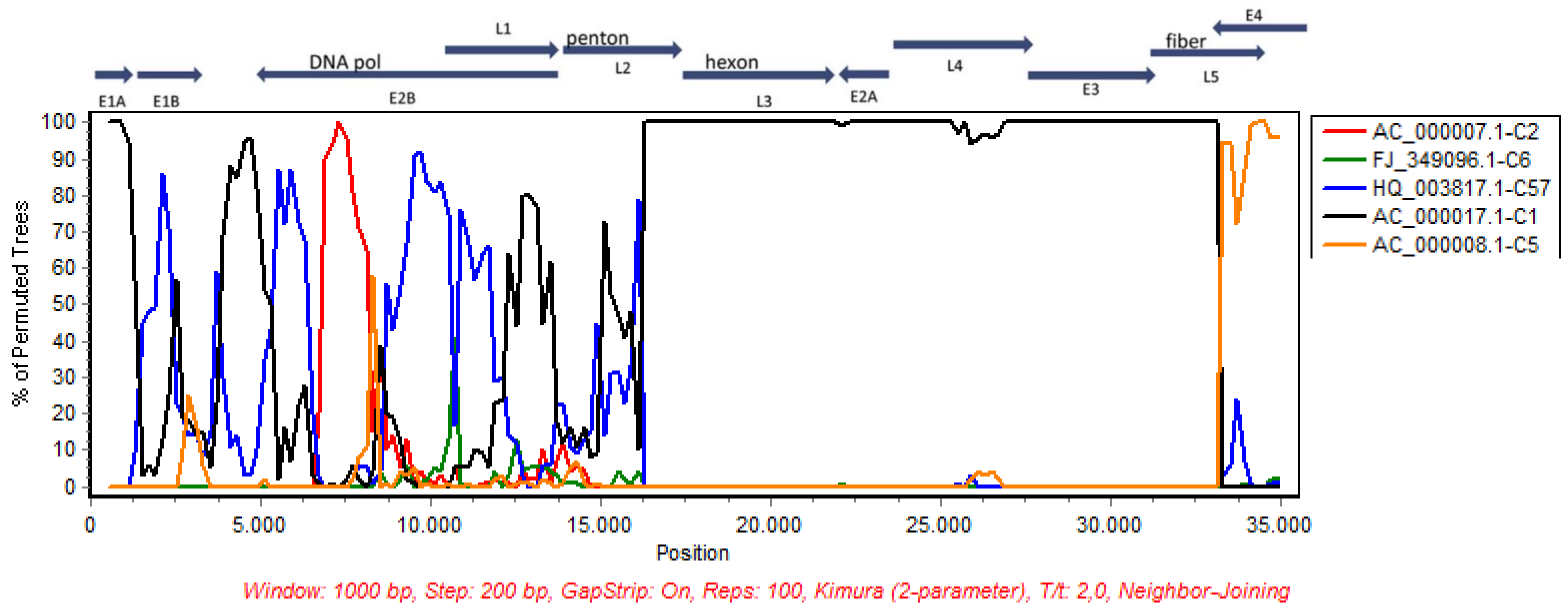

42C2

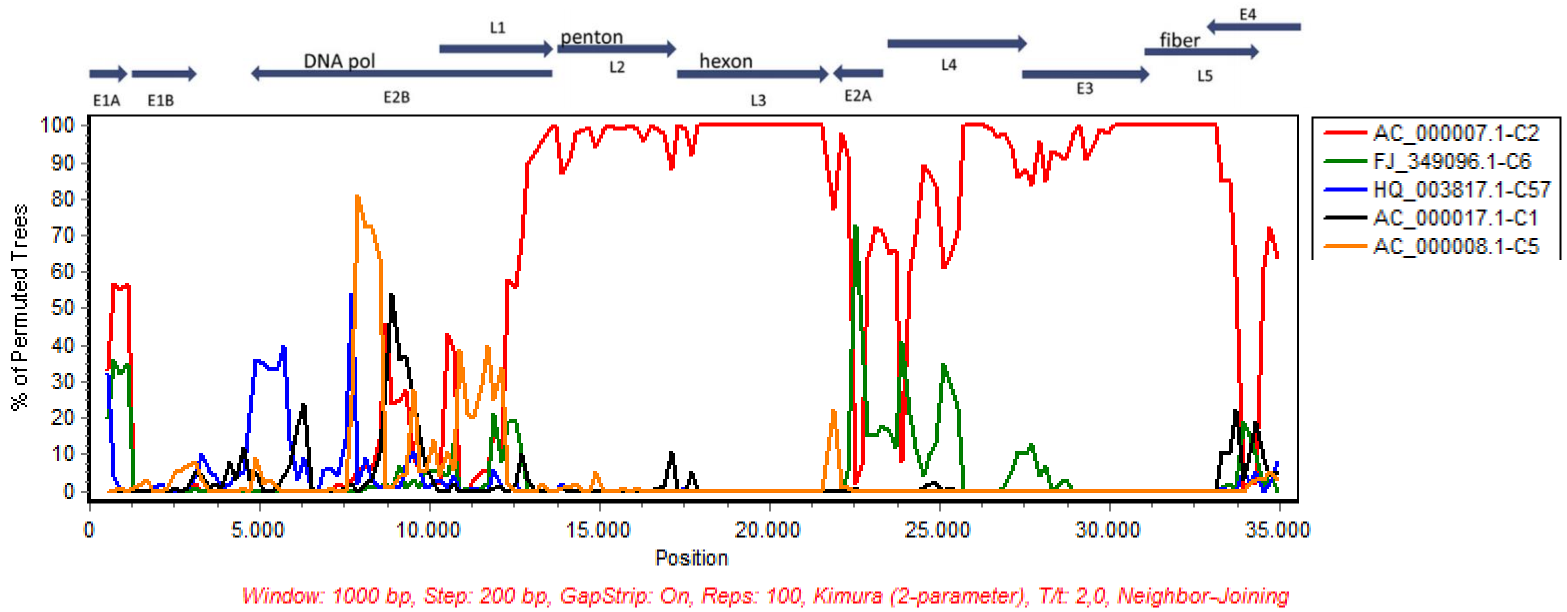

Figure S2

43C1

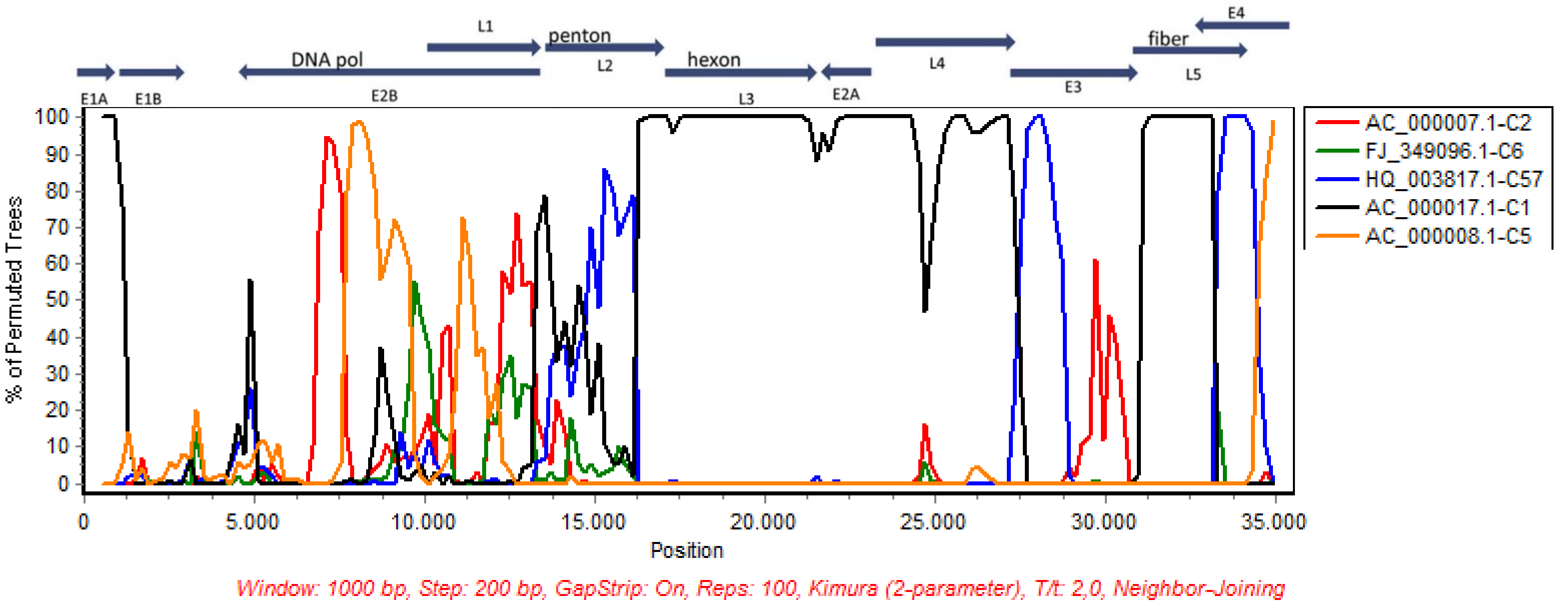

44C2

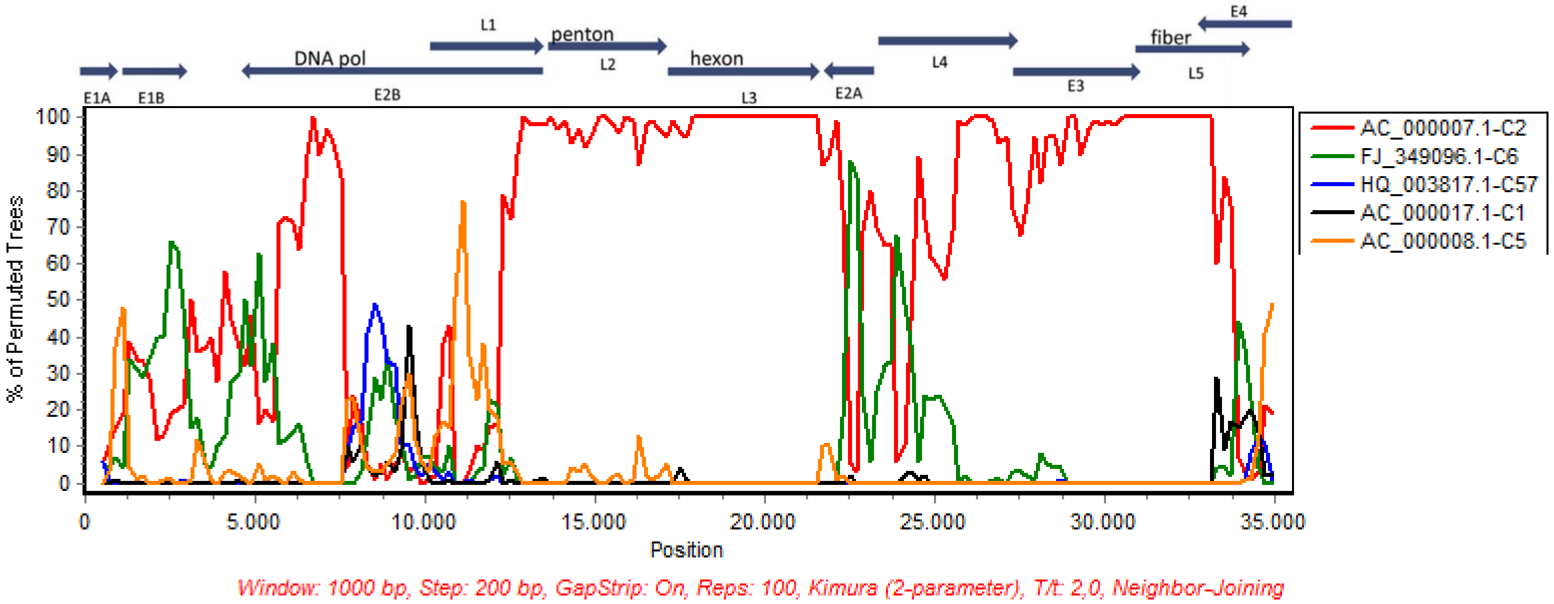

Figure S2

45C6

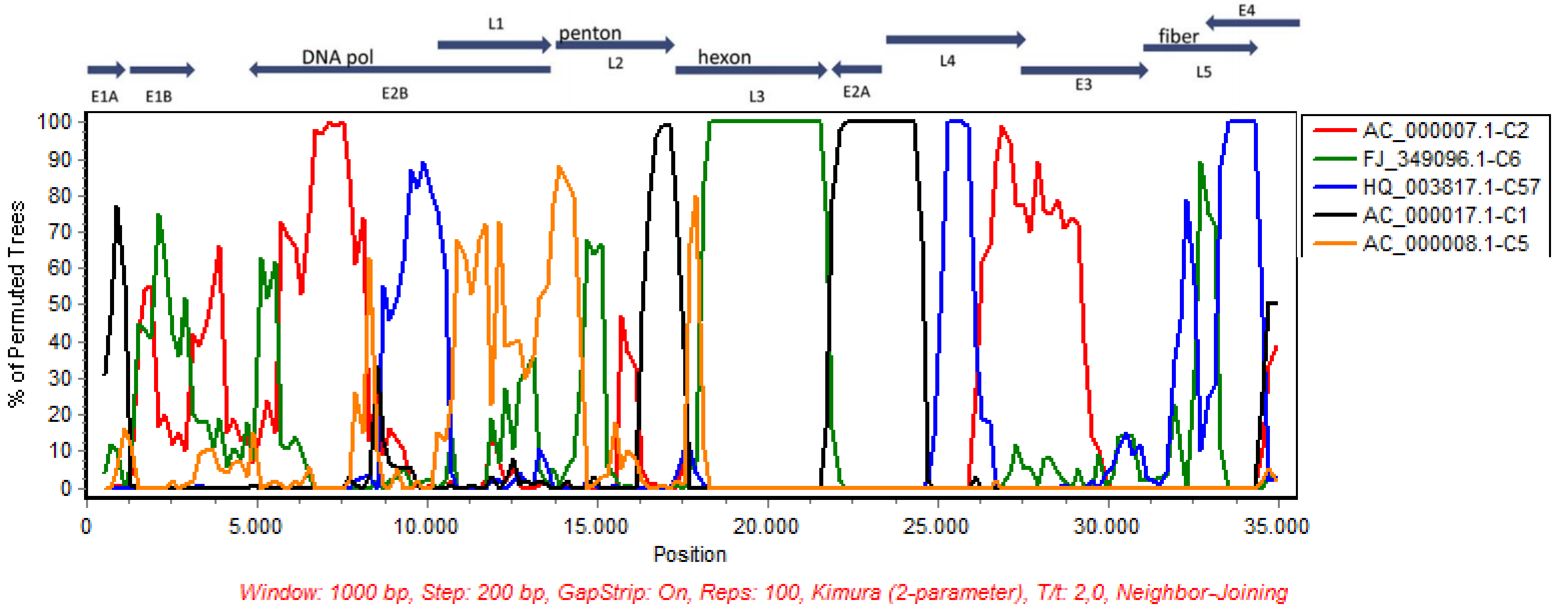

46C6

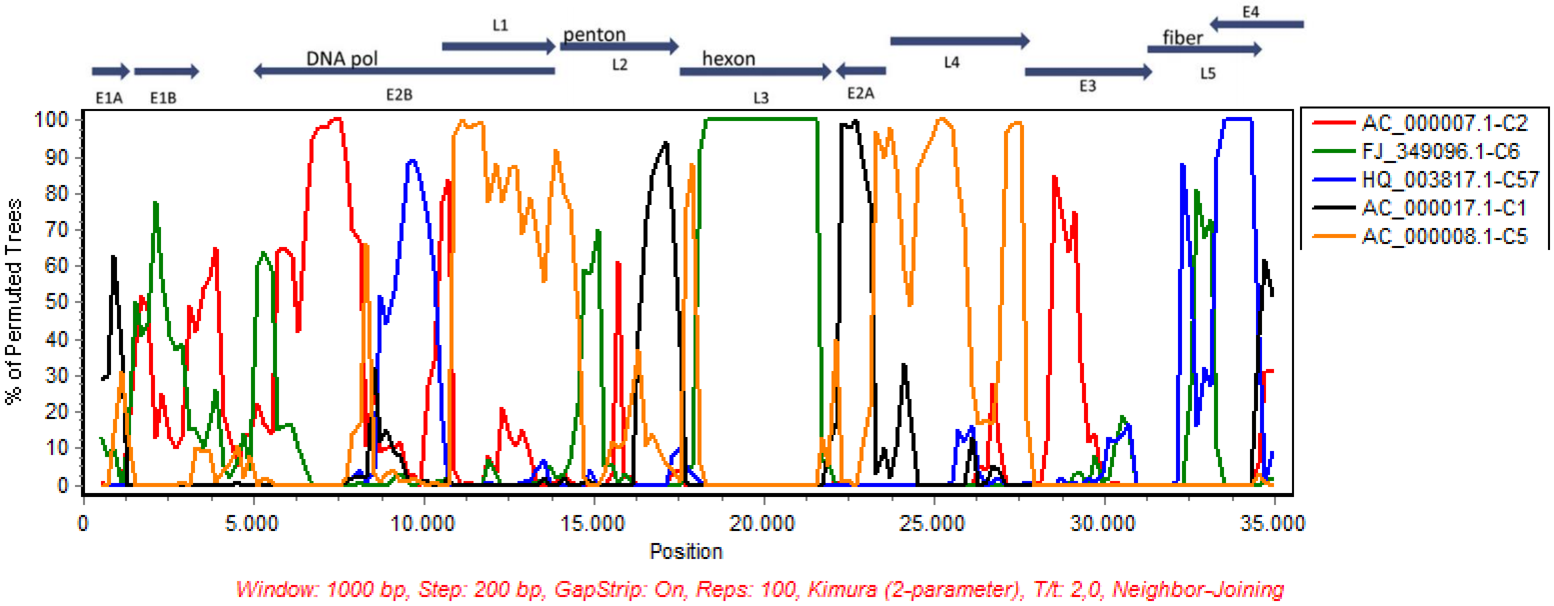

Figure S2

47C2

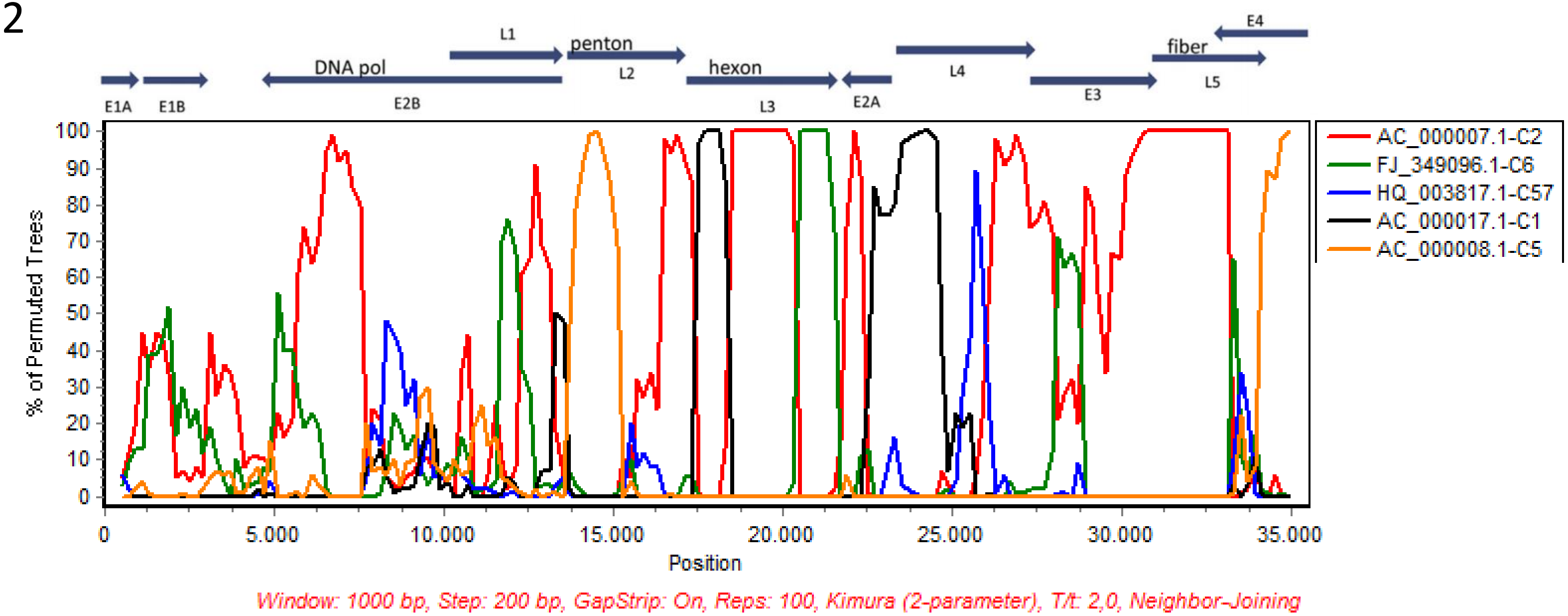

48C2

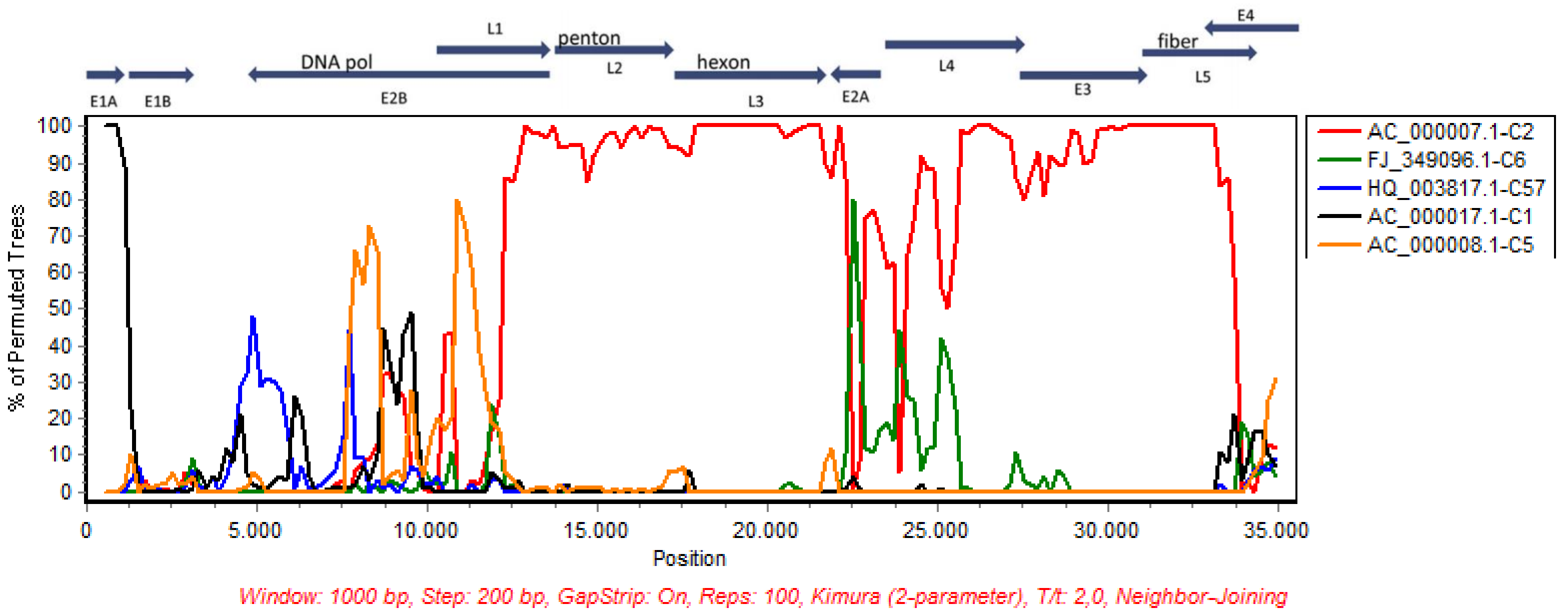

Figure S2

49C5

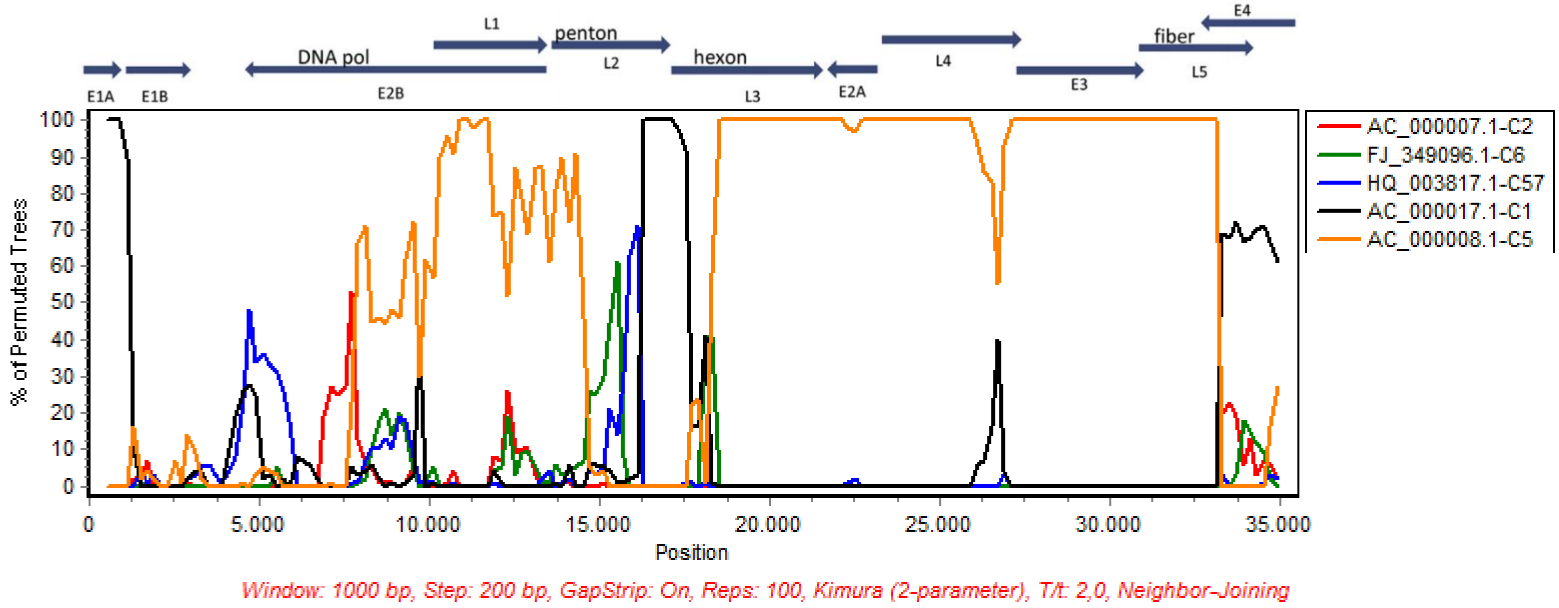

50C1

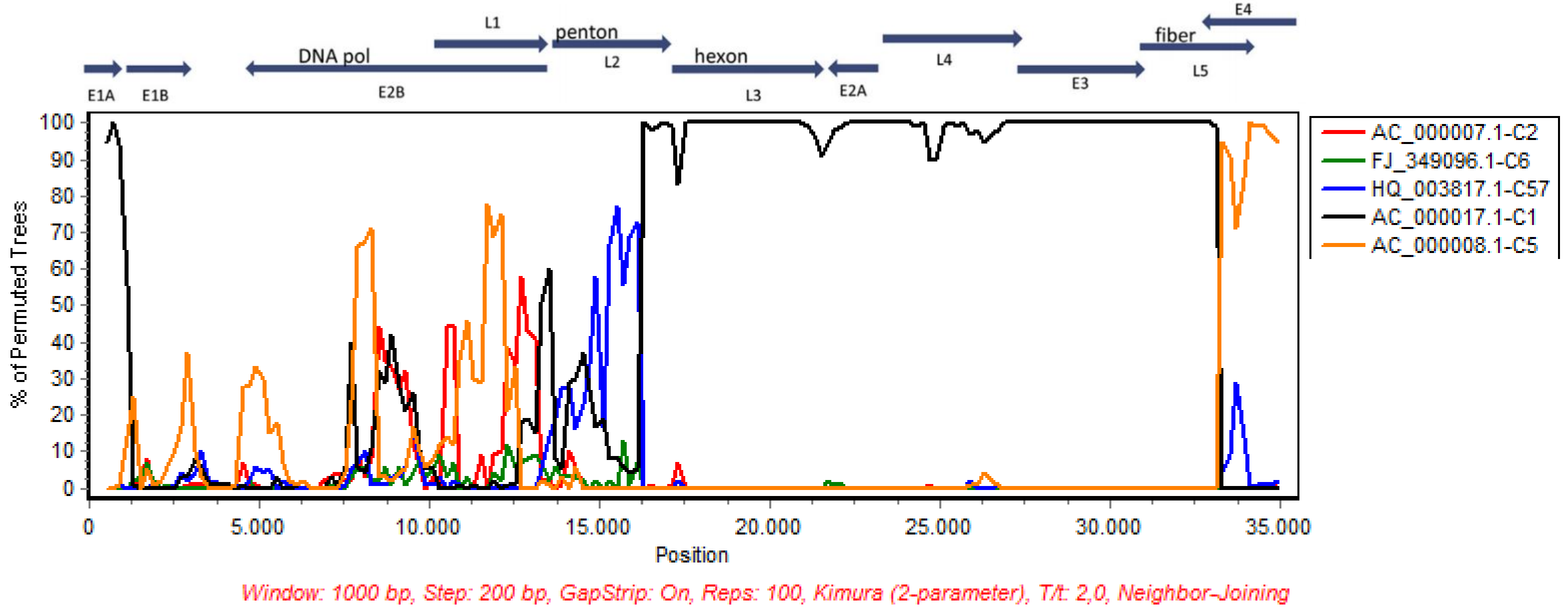

Figure S2

51C1

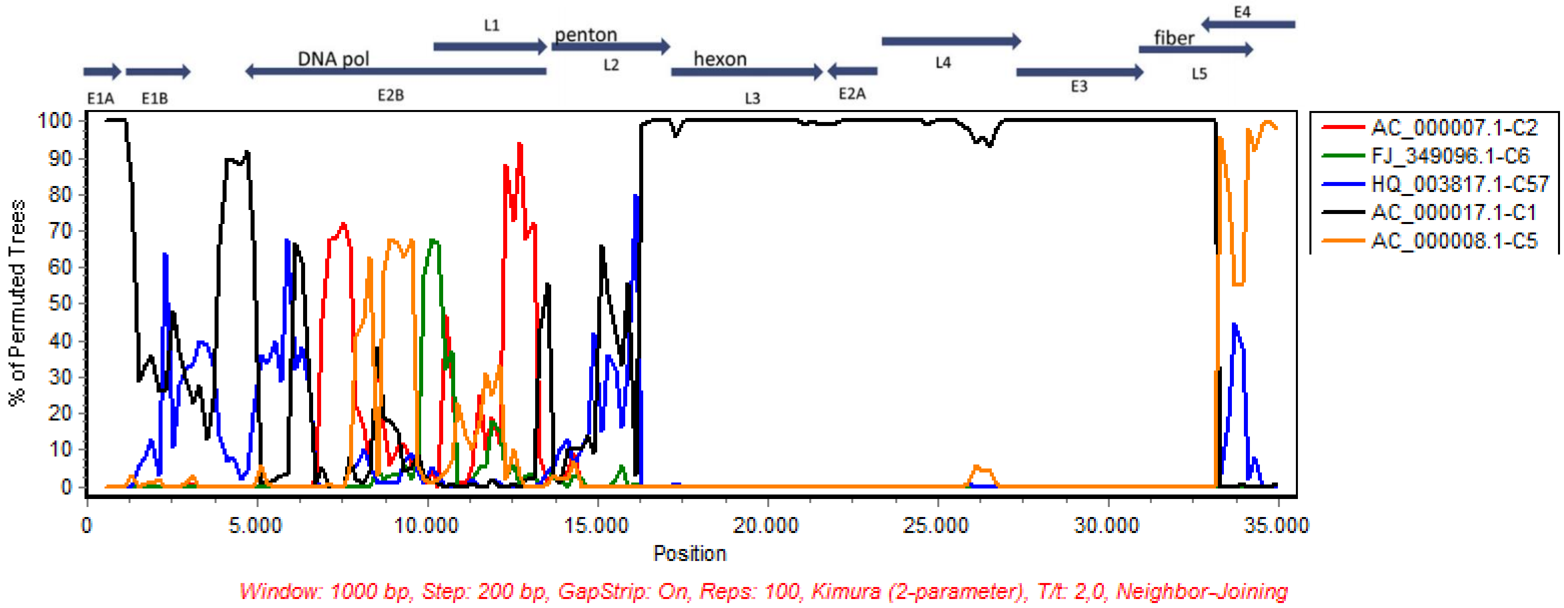

Figure S4: Phylogenetic analysis of the early gene regions E2A (A) and E2B (B). Clustering of circulating strains and prototype sequences (highlighted by a black dot, labelling indicates accession number-species and type). The neighbor-joining tree was generated based on the Kimura two-parameter model with MEGA7. Bootstrap value <80% are not robust and therefore not depicted. * Strains 29C2 and 47C2 were renamed as the novel type HAdV-C89.

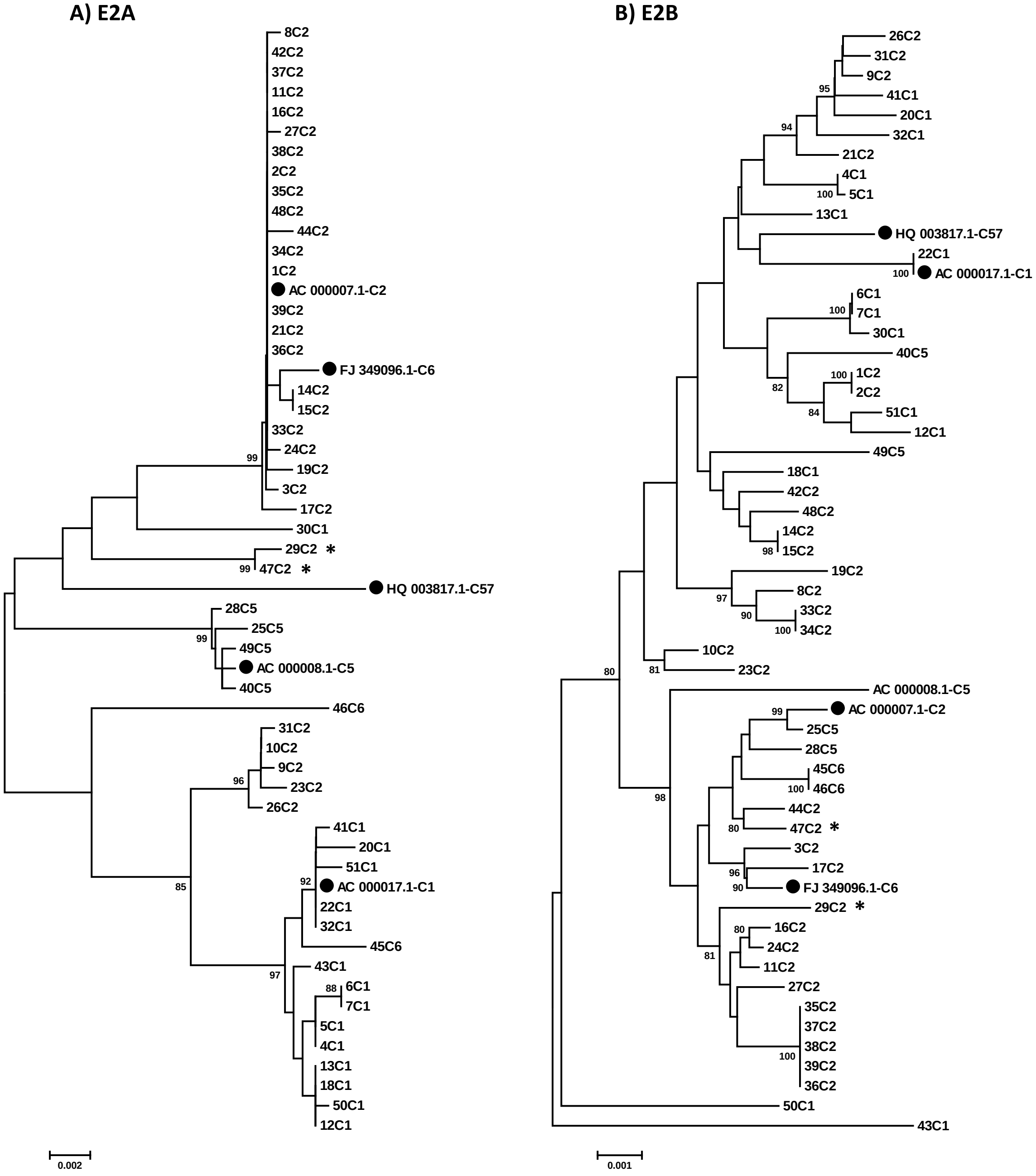

Supplement: Supplementary file 1 — Supplementary Dataset 1 [file 41598_2018_37249_MOESM1_ESM.pdf]
